# The Development of LAT1 Efflux Agonists as Mechanistic Probes of Cellular Amino Acid Stress

**DOI:** 10.3390/biom14030326

**Published:** 2024-03-09

**Authors:** Vandana Sekhar, Houssine Ikhlef, Alexandra Bunea, Viet S. Nguyen, Johan Joo, Mukund P. Tantak, Holly Moots, Otto Phanstiel

**Affiliations:** Department of Medical Education, College of Medicine, University of Central Florida, 12722 Research Parkway, Orlando, FL 32826, USA

**Keywords:** autophagy, methionine efflux, *S*-adenosylmethionine (SAM), lipid signaling

## Abstract

Amino acid restriction induces cellular stress and cells often respond via the induction of autophagy. Autophagy or ‘self-eating’ enables the recycling of proteins and provides the essential amino acids needed for cell survival. Of the naturally occurring amino acids, methionine restriction has pleiotropic effects on cells because methionine also contributes to the intracellular methyl pools required for epigenetic controls as well as polyamine biosynthesis. In this report, we describe the chemical synthesis of four diastereomers of a methionine depletion agent and demonstrate how controlled methionine efflux from cells significantly reduces intracellular methionine, *S*-adenosylmethionine (SAM), *S*-adenosyl homocysteine (SAH), and polyamine levels. We also demonstrate that human pancreatic cancer cells respond via a lipid signaling pathway to induce autophagy. The methionine depletion agent causes the large amino acid transporter 1 (LAT1) to preferentially work in reverse and export the cell’s methionine (and leucine) stores. The four diastereomers of the lead methionine/leucine depletion agent were synthesized and evaluated for their ability to (a) efflux ^3^H-leucine from cells, (b) dock to LAT1 in silico, (c) modulate intracellular SAM, SAH, and phosphatidylethanolamine (PE) pools, and (d) induce the formation of the autophagy-associated LC3-II marker. The ability to modulate the intracellular concentration of methionine regardless of exogenous methionine supply provides new molecular tools to better understand cancer response pathways. This information can then be used to design improved therapeutics that target downstream methionine-dependent processes like polyamines.

## 1. Introduction

This report describes the development of novel methionine depletion agents and explores the connection between intracellular methionine pools, *S*-adenosylmethionine (SAM) restriction, polyamines, and autophagy. Autophagy is a ‘self-eating’ process, where cells create specialized lipid vesicles (autophagosomes) to recycle biomolecules. The reclaimed building blocks are then used to build critical components (e.g., proteins) to meet the needs of the cell. Typically, this cell survival mechanism is induced by cell stressors such as nutrient restriction [1]. The benefits of autophagy include cell renewal and extended lifespan. For example, caloric restriction (CR) has been shown to extend lifespan in worms [2], flies [3,4], and rats [5,6,7]. Indeed, the Comprehensive Assessment of Long-term Effects of Reducing Intake of Energy (CALERIE) two-year study in humans recently demonstrated that CR modified risk factors for age-related diseases and influenced indicators associated with longer lifespan [8]. In summary, these intriguing studies suggest that one could extend lifespan by control of nutrient supply. Nutrient restriction (NR) can, of course, be accomplished by a restricted diet; however, dietary compliance will likely always be a major challenge in humans.

Another approach is to restrict the availability of a single amino acid without compromising the balance of other nutrients. Of all the amino acids, methionine restriction (MR) is unique. MR not only extends lifespan but also produces changes in one-carbon metabolism and increases autophagic capacity and mitophagy [9]. Indeed, a 2019 report by Plummer et al. demonstrated that the MR-mediated lifespan increases in yeast involved and required the autophagic recycling of mitochondria [9].

**Figure 1:**
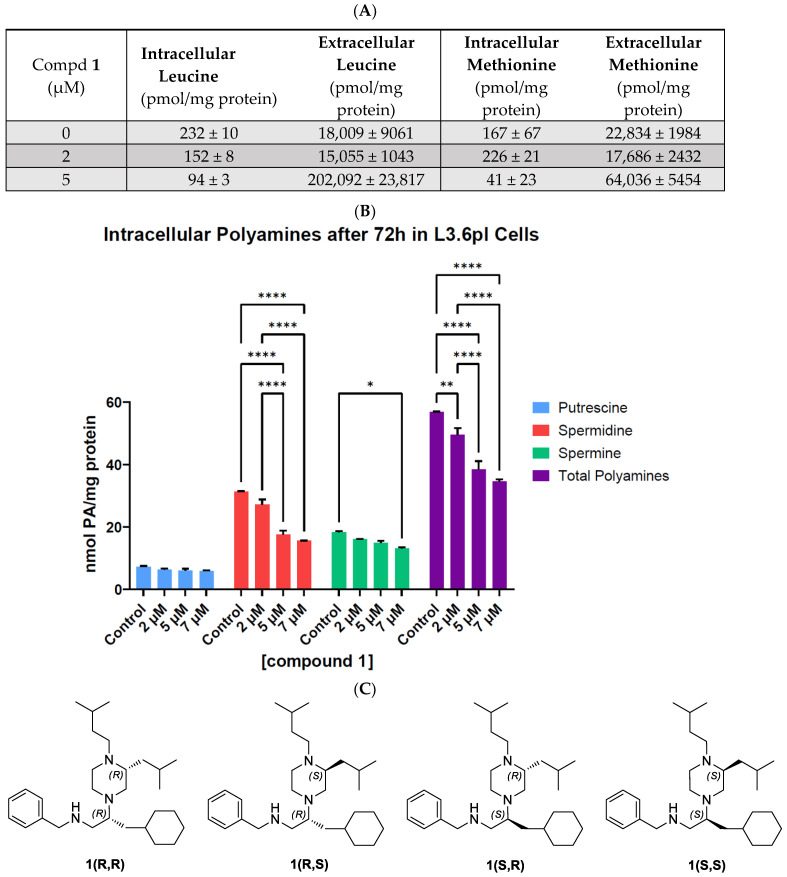
(**A**) Table showing the intra- and extra-cellular concentration of leucine and methionine in L3.6pl human pancreatic cancer cells after 72 h of incubation with increasing concentrations of compound **1** (RS isomer). LC-MS was used to measure each amino acid analyte. Extracellular levels were obtained from supernatant collection and intracellular levels were obtained after washing steps and lysis of the remaining cells. The data were normalized by dividing by the mg protein. Details of this method are described in Massaro et al. [10]. At least 3 independent experiments were performed, and samples were run in duplicate. Data are presented as mean ± SEM. (**B**) Dose-dependent polyamine depletion in the presence of **1** in L3.6pl pancreatic cancer cells as shown previously [10]. L3.6pl cells were dosed with increasing concentrations of compound **1** (RS isomer) and cells were incubated for 72 h at 37 °C. The untreated control was run in parallel and polyamine levels were determined in duplicate via *N*-dansylation and analysis by HPLC [17]. The data were averaged and reported as nmol polyamine (PA)/mg protein. * (*p* value ≤ 0.05), ** (*p* ≤ 0.01), **** (*p* ≤ 0.0001). (**C**) The structures of the four possible stereoisomers of compound **1**.

Recently, we described the discovery of a novel methionine efflux agonist **1** (RS isomer; Figure 1) [10]. The large amino acid transporter 1 (LAT1; aka SLC7A5) normally works in concert with the alanine–serine–cysteine transporter 2 (ASCT2; aka SLC1A5) to exchange a small intracellular neutral amino acid for a large extracellular essential amino acid like leucine or methionine. In this mode, LAT1 works as an importer of methionine and leucine. Working in reverse or leaky mode, LAT1 can export large amino acids from inside the cell. Indeed, a recent report by Bothwell et al. suggested that LAT1 is a ‘harmonizer’ and not a driver of amino acid accumulation [11]. Other investigators have demonstrated the role of LAT1 in regulating mTOR [12] and how LAT1 uses different conformations to complete its transport cycle [13]. Due to its reversible modes, the uptake and export of radiolabeled leucine are accepted assays used to assess LAT1 function [14]. Our prior work demonstrated that compound **1** stimulated the loss of LAT1 substrates (e.g., leucine, methionine, and phenylalanine) from L3.6pl pancreatic cancer cells. Using L3.6pl cells preloaded with radiolabeled leucine, we demonstrated that the efflux of labeled leucine was reduced in the presence of a LAT1 inhibitor (JPH203) [15,16], suggesting that LAT1 was the target of **1** [10].

We investigated how cells respond to the dose-dependent reduction in cellular methionine pools imparted by **1**. Since methionine is required for SAM biosynthesis and decarboxylated SAM (dc-SAM) is required for polyamine biosynthesis, we hypothesized that agents like **1** could be used to reduce intracellular polyamines by limiting SAM supply. SAM restriction should have a profound effect on polyamine homeostasis as it provides the essential aminopropyl unit needed to build the higher polyamines: spermidine and spermine. In this report, we demonstrated the anticipated reduced SAM supply consistent with this hypothesis. We also demonstrated for the first time how controlled methionine efflux from cells with **1** stimulates a lipid signaling pathway (previously identified in yeast) to induce the autophagy marker LC3-II in human pancreatic cancer cells. Moreover, to understand the role of stereochemistry in the design of compound **1**, the four diastereomers of **1** were independently synthesized and evaluated for their ability to (a) induce leucine (i.e., the gold standard LAT1 substrate) efflux from cells, (b) preferentially bind in silico to an allosteric pocket on the large amino acid transporter 1 (LAT1), (c) decrease intracellular *S*-adenosylmethionine (SAM), *S*-adenosylhomocysteine (SAH), and phosphatidylethanolamine (PE) pools, and (d) induce the formation of autophagosomes via the autophagy-associated lipid LC3-II. In summary, in this study, we demonstrate the ability of **1** to reduce SAM and SAH stores, decrease polyamine pools, and increase LC3-II, a marker consistent with autophagy induction.

## 2. Results and Discussion

We previously demonstrated that compound **1** causes a net efflux of radiolabeled leucine from cells and does not inhibit the uptake of leucine [10]. This LAT-1 efflux agonist lowered intracellular leucine and methionine levels (Figure 1A) and inhibited the growth of L3.6pl human pancreatic cancer cells [10]. As shown in Figure 1B, compound **1** (RS isomer) also generated a dose-dependent reduction in intracellular polyamines as anticipated [10]. Since **1** contained two stereocenters, we synthesized each of the four stereoisomers of **1** (RR, RS, SR, and SS stereoisomers, Figure 1C) to better understand how the compound’s orientation in three-dimensional space affected its performance. Molecule **1** essentially has four hydrophobic groups oriented away from a core piperazine platform. What is fascinating about the design is that two of the appended hydrophobic groups (cyclohexyl and phenyl) can be interchanged via rotation around a single C-N bond. Moreover, both the top and bottom halves of the molecule have five atoms between lipophilic branch points suggesting that the molecule could flip over and still present a similar chemical message to the LAT1 target. This ability to dynamically ‘interchange’ its substituents via bond rotations (and molecular flips) posed an interesting question: Does stereochemistry matter in this design? With this nuance in mind, we embarked on the synthesis of these targets to compare their in vitro performance and ability to modulate SAM pools.

### 2.1. Synthesis

**Scheme 1:**
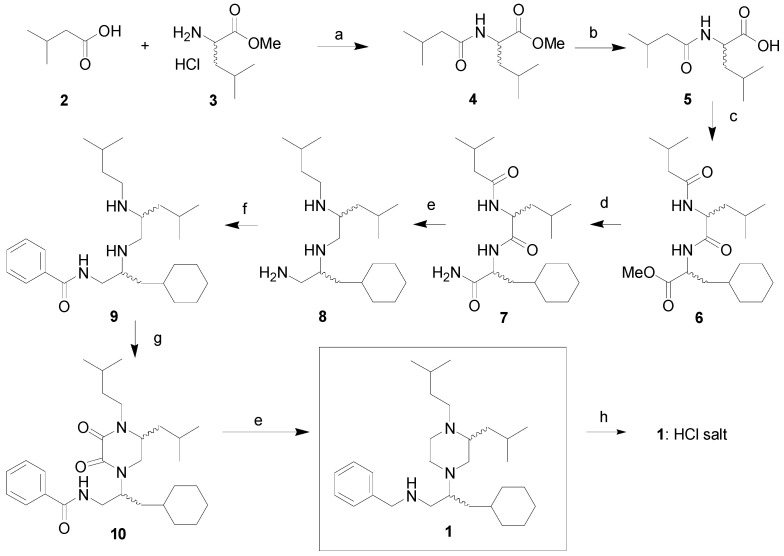
*Reagents*: (a) HATU, DIEA, and DCM (average 70% yield); (b) 1 M NaOH at 0 °C in MeOH, and then HCl (yields ranged from 64–73%); (c) D- or L-cyclohexylalanine methyl ester, HATU, DIEA, and DCM (yields ranged from 64–79%); (d) NH_4_OH and MeOH (89–93% yield); (e) BH_3_-THF and reflux (96–97% in forming **8** as crude oil and 43–63% yield in forming pure **1**); (f) 2,5-dioxopyrrolidin-1-yl benzoate, DCM, 53–66% yield; (g) oxalyl diimidazole (5 equiv), DCM, rt, 65–75% yield); (h) 4 M HCl in EtOH, 62–81% yield. Note: compound **8** was isolated after workup as a crude oil and immediately *N*-benzoylated to form **9**.

The initial hit compound **1** (RS)-isomer was originally obtained from the Torrey Pines Institute of Molecular Studies as part of the Florida Drug Discovery Acceleration program. The nomenclature (RS) denotes that the stereochemistry outside the piperazine ring is in the R configuration, whereas the chiral center inside the piperazine ring is in the S configuration. The opposite is true for the SR isomer, wherein the exocyclic stereochemical center is S and the endocyclic stereochemical center is R (Figure 1C). Compound **1** was initially generated utilizing solid phase synthesis methods, which produce a small amount of material that can be used for biological screening studies. We developed a modular synthetic scheme (Scheme 1) to access the four possible stereoisomers (RR, RS, SR, and SS) and evaluated their ability to inhibit L3.6pl cell growth and deplete intracellular *S*-adenosylmethionine (SAM) levels.

Retention of configuration was paramount and the HATU coupling agent was employed to minimize racemization during the key coupling steps. HATU is a coupling reagent that works with a non-nucleophilic base, Hünig’s base (diisopropylethylamine, DIEA) [18], to form a 1-oxy-7-azabenzotriazolate active ester [19]. The active ester formed is then reacted with the corresponding amine to form the amide bond with little to no racemization. Note: this retention of chiral integrity was later confirmed by the circular dichroism (CD) spectra of the final products (see Supplementary Materials).

As shown in Scheme 1, isovaleric acid **2** was coupled to either the D- or L-isomer of leucine methyl ester **3** to form the respective chiral amidoester **4** in 70% yield. ^1^H NMR (CDCl_3_) confirmed conversion via the appearance of a triplet of doublets at 4.66 ppm that integrated for one hydrogen, representative of the unique α-hydrogen on the alpha carbon of **4**.

Each stereoisomer of ester **4** was then respectively hydrolyzed using 1 M NaOH/MeOH to form the corresponding acid **5** after workup. ^1^H NMR (DMSO-d_6_) confirmed the conversion of **4** in good yield (79%) by the loss of the methyl ester peak at 3.73 ppm. Each acid was then coupled to either D- or L-cyclohexylalanine methyl ester using HATU to give the respective diamidoester **6**. Successful coupling was performed in good yields (~72%) and was monitored by ^1^H-NMR (CDCl_3_) for the formation of a multiplet at 4.57 ppm (integrating for two hydrogens) consistent with the α-hydrogens in **6**.

Aminolysis of **6** using NH_3_ gas (generated from a concentrated NH_4_OH solution warmed to ~35 °C) gave the respective triamide **7** (as a precipitated product) in high yields (90%). ^1^H NMR confirmed production of the triamide by loss of a methyl ester peak at 3.73 ppm. The respective triamide **7** was then reduced to triamine **8** using BH_3_-THF. The borane complexes that remained were removed with a 10% MeOH/HCl solution that formed volatile B(OMe)_3_, which was removed during evaporation of the solvent [20].

Regiospecific *N*-benzoylation of the primary amine of **8** was accomplished using 2,5-dioxopyrrolidin-1-yl benzoate in DCM to give the benzamide **9** (~66%). The regiochemistry of the N-benzylation was confirmed by ^1^H-NMR (CDCl_3_) in which the two diastereotopic hydrogens of the CH_2_NHCOPh group integrated for one hydrogen each at 3.59 and 3.27 ppm, respectively. In contrast, the other aliphatic amine ^1^H-NMR signals were all at chemical shifts less than 3 ppm. Note: if *N*-benzoylation had occurred on one of the secondary amine positions of **8**, one would have expected three downfield ^1^H NMR signals between 3 and 3.6 ppm.

The formation of the respective diketopiperazine **10** was accomplished in 75% yield by reacting the respective isomer of **9** with five equivalents of oxalyl diimidazole at rt [21]. The final reduction of **10** with BH_3_-THF afforded each final stereoisomer of **1** as a single compound in typically 43–63% yield. Alternatively, these compounds could be readily converted to their respective HCl salts for long-term storage. Conversion to the corresponding hydrochloride salt was performed in 4 M HCl/EtOH to give the final product **1**. In summary, the selection of either the R- or S-isomers of **3** or cyclohexylalanine methyl ester allowed for the generation of each isomer of **1** (RR, RS, SR, or SS).

As noted earlier, the CD spectra were obtained for the four isomers and were consistent with maintained chiral integrity throughout the synthesis (Supplementary Materials). Indeed, the HATU coupling agent was selected due to its known ability to inhibit racemization and maintain chiral integrity [22]. Moreover, inspection of the ^1^H NMR spectra of the four diastereomers of compounds **9**, **10**, and **1** all suggested that little to no racemization occurred during the synthesis of **1** (see Figure S3 in the Supplementary Materials).

### 2.2. Bioevaluation

Each stereoisomer of compound **1** was then screened for growth inhibition in L3.6pl cells, which are hyper-metastatic human pancreatic cancer cells that have undergone six cycles of metastatic training in mice [23]. Growth inhibition was measured by performing a 24 h experiment at two different cell densities, using the MTS colorimetric assay in which metabolic activity was detected by the ability of cells to convert the yellow tetrazolium MTS dye (3-(4,5-dimethylthiazol-2-yl)-5-(3-carboxymethoxyphenyl)-2-(4-sulfophenyl)-2H-tetrazolium) to the purple formazan reporter [24]. Interestingly, the four isomers of compound **1** had similar IC_50_ values, i.e., the concentration of the compound in which 50% of the relative cell growth was inhibited compared with an untreated control at both cell densities (Table 1). As expected, there was a slight increase observed in the IC_50_ values at the higher cell density. However, similar responses indicated that stereochemistry had little effect on the growth inhibition properties in these cells when measured over a 24 h period. We also noted that these compounds demonstrated increased potency in growth inhibition over longer incubation times (48 h and 72 h) as reflected by their lower IC_50_ values in L3.6pl cells (Table S2).

This was an interesting result as we anticipated that a specific diastereomer would be superior in terms of LAT-1 efflux agonism. We hypothesized that measurements at the longer incubation time of 24 h may miss the distinctions in efflux agonist performance. For example, it may be difficult to distinguish fast methionine efflux after 30 min of incubation vs a slower methionine efflux over 8 h, when one observes the system only after a 24 h incubation period. With this insight in mind, we turned to short kinetic experiments to better investigate the diastereomeric performance of **1**.

### 2.3. Amino Acid Transport Studies

Previous work with the RS isomer of **1** demonstrated that incubation with L3.6pl cells for 72 h resulted in both a significant increase in leucine, methionine, and phenylalanine in the supernatant and a concomitant decrease in these amino acids inside the cell. Since leucine, methionine, and phenylalanine all utilize LAT1 for cell entry and exit, we surmised that LAT1 was the likely target of **1**. Further studies with ^3^H-leucine and JPH203 (a known LAT1 inhibitor, Supplementary Materials) [15] demonstrated that compound **1** (RS) did not block the uptake of leucine, but rather facilitated efflux of leucine. Subsequent experiments with **1** (RS) and the specific LAT1 inhibitor JPH203 demonstrated that JPH203 inhibited the compound **1**-induced efflux of leucine from these cells [10]. Therefore, we concluded that LAT1 was the target of **1** [10].

**Figure 2:**
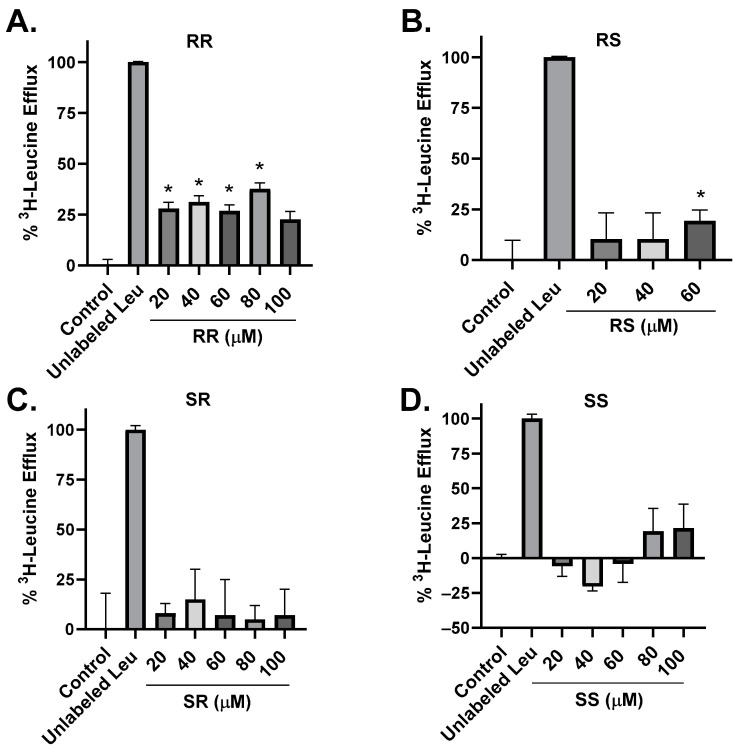
Dose-dependent efflux assay demonstrating the ability of each isomer of **1** (Panel **A**: RR, Panel **B**: RS, Panel **C**: SR and Panel **D**: SS) to directly stimulate efflux of ^3^H-leucine compared with 100 μM unlabeled leucine and the PBS control after 30 min. The unlabeled leucine control provided the benchmark and was always set to 100% for comparisons. Asterisks denote that the *p*-value is 0.05 or less.

The four isomers of **1** were evaluated in a leucine efflux assay, wherein L3.6pl cells were pre-loaded with ^3^H-leucine and then thoroughly washed. The efflux of ^3^H-leucine from inside the L3.6pl cells was then monitored in the supernatant after 30 min of incubation in the presence and absence of each compound. Unlabeled leucine (100 µM) provided a positive control for these experiments in Figure 2. This leucine control works by a trans-stimulation pathway wherein the exogenous unlabeled leucine is imported via LAT1 and exchanged with intracellular ^3^H-labeled leucine, which is exported. This efflux process provided a reproducible ^3^H-leucine signal in the supernatant to benchmark each experiment and the y-axis is shown as % ^3^H-leucine efflux relative to this control, which was always set to 100%.

As shown in Figure 2, we observed a statistically significant increase in radiolabeled leucine efflux for the RS and RR isomers of **1** during this short 30 min time period. Specifically, we observed ~20–25% leucine efflux for both the RS and RR isomers at 60 µM (Figure 2). In contrast, the SS and SR isomers had little efflux stimulation at this 60 μM dose in the time frame studied. The RR isomer was more potent and produced significant leucine efflux at 20 µM compared with the other three isomers. In summary, of the four isomers, these 30 min efflux kinetic studies suggested that the RR- and RS-isomers of **1** were superior efflux agonists in vitro. The growth inhibition data in Table 1, however, suggested that given enough time (24 h) even the lower-performing efflux agonists (SS and SR isomers) could significantly affect pancreatic cancer cell growth.

### 2.4. Molecular Modeling

**Figure 3:**
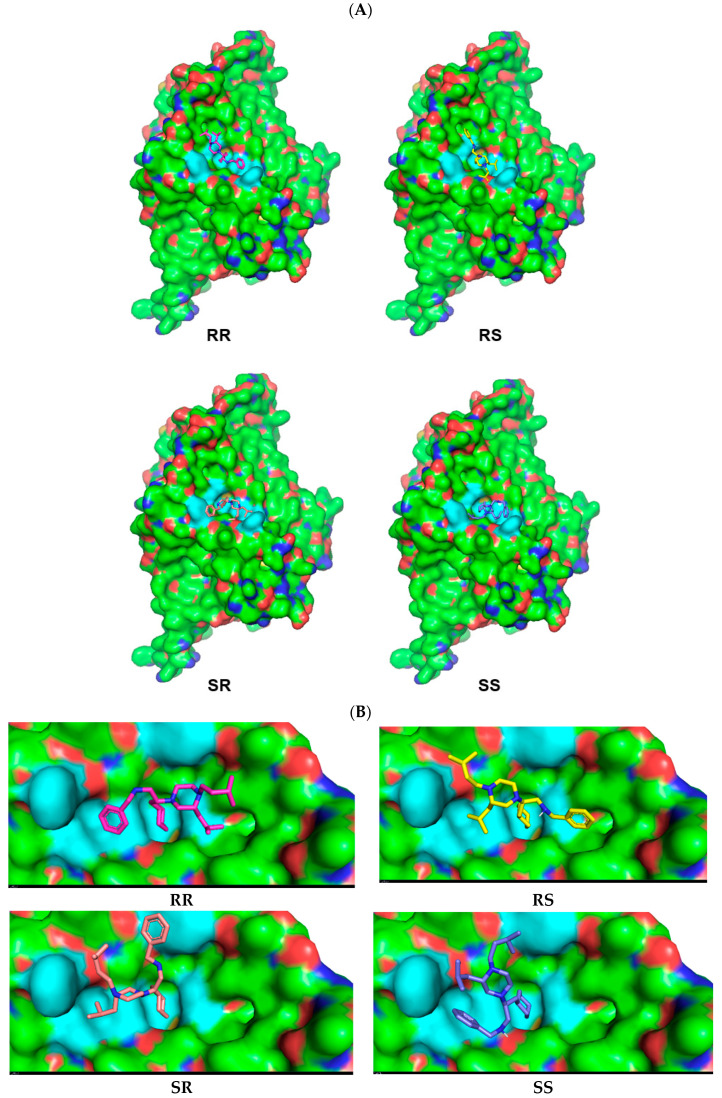

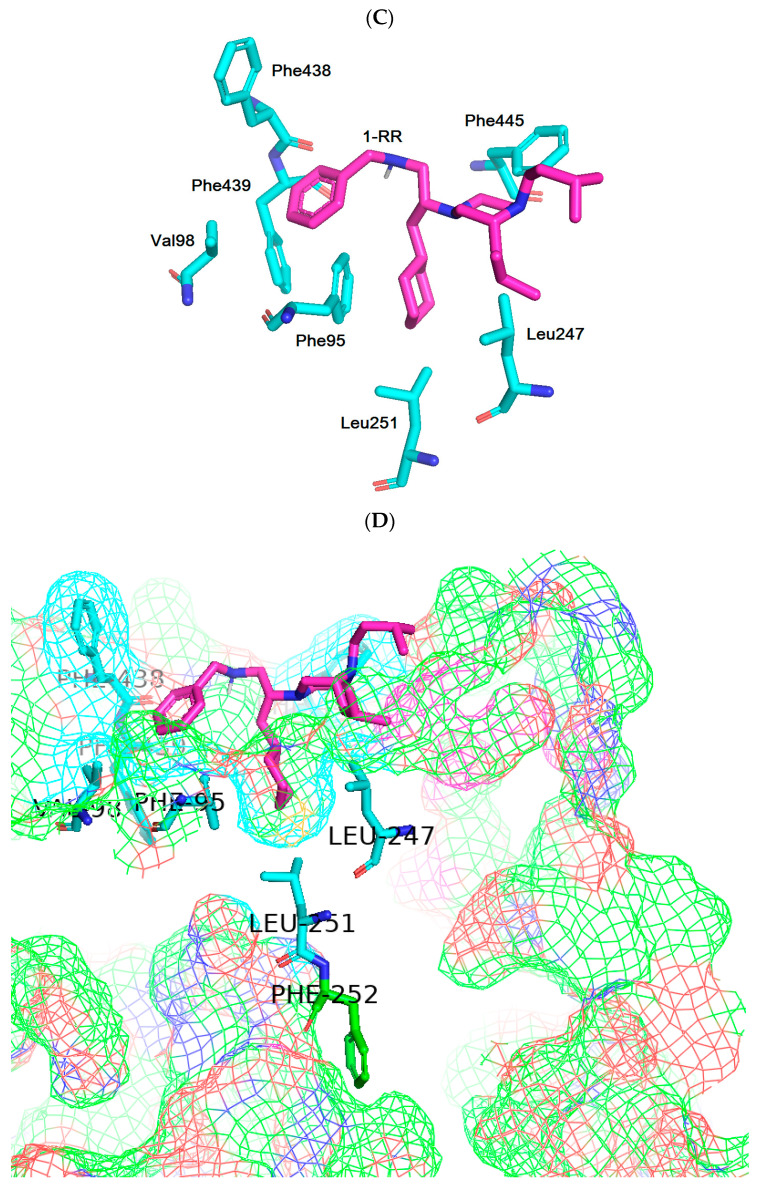
(**A**) LAT-1 bound to each of the four isomers of **1**. The relative binding affinities of these isomers for LAT1 were all similar in silico (RR: −6.9; RS: −7.0; SR: −7.2; and SS: −7.2 kcal/mol) but each isomer also made unique contacts with the LAT1 surface beyond the cyclohexyl/pore interaction. (**B**) (4 panels) Zoomed-in view of each isomer of **1** docked to the LAT1 pore. We note that the SR and SS isomers do not prefer to interact with the right-side pocket of this allosteric site as shown in (**B**). Relevant LAT1 residues interacting with **1** (RR) (**C**) and the proximity of this putative binding site to the gatekeeper Phe252 (**D**). The multi-colored wireframe model shows the relevant LAT1 protein scaffold with key nearby residues highlighted in teal color and the gatekeeper residue Phe-252 shown in bright green.

Next, we used the recently published 3D structure of LAT1 (pdb: 6irs) to dock each of the four isomers of **1** in silico. Each isomer was drawn in ChemDraw, imported and energy-minimized (via MM2) via Chem3D, and saved as a pdb file. Each isomer (pdb file) was then imported into the PyRx program and docked to LAT1. The final top-scoring docked structure (lowest binding affinity) was then visualized using PyMol and is shown in Figure 3 (A-wide view) and (B-zoom view). Interestingly, all four isomers docked to the same location on the LAT1 protein (Figure 3) and oriented their cyclohexyl substituent into the same pore motif (Figure 3). This LAT1 allosteric pore allowed all isomers of compound **1** to directly interact with key residues in the interior of the protein.

As shown in Figure 3C, the modeling suggests that the cyclohexyl substituent interacts with the LAT1 pore defined by Phe95, Leu247, and Leu251. Leu247 and the adjacent Ala246 residue are located in the transmembrane domain 6 (TM6) of the LAT1 protein and are important for the helical packing of TM1 and TM6 of LAT1 [25]. In addition, Leu251 is directly adjacent to the phenylalanine residue, Phe252, which is a decisive proximal gate residue in LAT1 [25,26]. In short, molecular modeling suggested that the efflux agonist may work by altering the local environment of key residues on LAT1, which alters TM1 and TM6 interactions and repositions Phe252 to facilitate efflux of LAT1 substrates.

While the preliminary modeling work has its limitations, it does nevertheless provide a visual explanation for why the RR and RS isomers performed better in the efflux assay at 60 µM. As shown in Figure 3C, these isomers provide a greater number of interactions with the key LAT1 pore region using their phenyl, cyclohexyl, and isobutyl motifs. Remarkably, the RS isomer of **1** is oriented approximately 180° in this same region as the RR isomer suggesting that the phenyl and isobutyl motifs are both suitable ligands for the pore-flanking pocket regions on the LAT1 surface. In contrast, due to their unique configurational constraints, the SR and SS isomers have fewer interactions with the flanking LAT1 pore pockets (see legend in Figure 3C), which may explain their lower potency at 60 µM in the kinetic experiments in Figure 2. In summary, while all four isomers dock their cyclohexyl substituent into the key LAT1 pore to access the gatekeeper Phe252, the RR and RS isomers appear to interact with both the pore and more of the LAT1 surface (Figure 3). Future experiments will seek to confirm these in silico predictions via co-crystallization experiments.

The premise of this investigation was to elucidate which of the four isomers of **1** was the most potent at targeting LAT1. We also recognized, however, that **1** has inherent ‘quasi symmetry’ imparted by the mobility of the cyclohexyl and phenyl rings as they can swing into position via a rotatable C-N single bond, regardless of the isomer used. Moreover, the branched alkyl chains can substitute for each other (or with the aryl or cyclohexyl group) to establish key hydrophobic interactions with the target. We likened this quasi-symmetry to a nano-helicopter with two interchangeable skids. Regardless of which way it lands, the propeller can rotate into the correct position to be strapped down for storage or, in this case, bind to its target.

Using this insight, the differences between the MTS and efflux assay results could be explained by the time of exposure to a particular isomer of **1**. For example, all four isomers inhibited L3.6pl cell growth after a 24 h incubation period. Therefore, given enough time they can all inhibit cell growth as measured by the MTS assay (Table 1). We speculate that due to the nature of the LAT-1 allosteric binding pocket and the unique ‘quasi-symmetry’ of the molecule itself, all four isomers of **1** are able to place a key group into the pocket controlling the gatekeeper residue and affect LAT1 function. Given enough time (24 h), each isomer is capable of depleting LAT1 substrates sufficiently to affect growth as seen in Table 1, even those isomers with lower measured 30 min LAT1 efflux agonism. In this regard, we may be observing the difference between molecules that induce a fast (RR) or slow ‘bleed rate’ of LAT1 substrates (SR). This bleed rate/efflux agonism distinction is best observed in short time frames. Indeed, the 30 min efflux experiments provided the following preferences for LAT1 efflux agonism (at 60 µM): RR > RS > SR > SS (Figure 2). Lastly, the modeling work suggested a potential explanation for this rank order as being derived from the number of interactions each isomer prefers to make with the LAT1 allosteric site (Figure 3).

### 2.5. Intracellular S-adenosylmethionine (SAM) Levels

**Figure 4:**
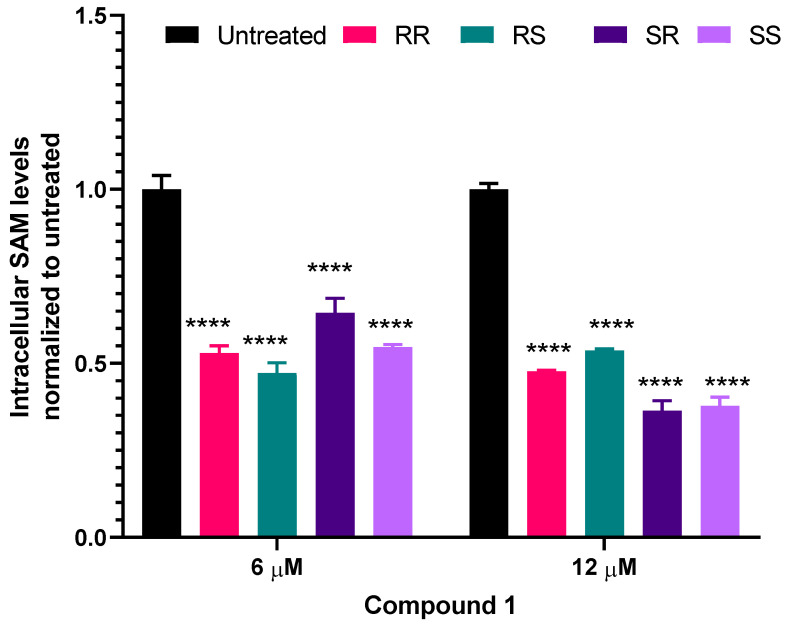
Graphical representation of the intracellular levels of SAM detected by HPLC in cells treated with either 6 µM or 12 µM of each diastereomer. The intracellular SAM levels are shown normalized to the respective untreated control from each experiment wherein each treatment was performed in triplicate. The data are representative of the mean +/− SD of three independent experiments (**** *p* ≤ 0.0001 vs. the untreated control).

Since **1** causes a dose-dependent loss of methionine from cells, we investigated how L3.6pl pancreatic cancer cells respond to decreased levels of intracellular methionine. Methionine plays many important roles in cells ranging from its direct role in protein synthesis to major metabolic and epigenetic contributions via its derivative, SAM. We examined the intracellular levels of SAM via HPLC analysis in L3.6pl cells following treatment with compound **1** for 24 h. We anticipated that since methionine is the biological precursor to SAM, compounds like **1,** which decrease intracellular methionine levels, would also reduce intracellular SAM pools as well as polyamine pools (Figure 1B). Indeed, treatment of L3.6pl cells with 6 μM or 12 μM of each isomer of **1** (24 h) resulted in a statistically significant reduction in intracellular SAM pools (Figure 4). While all four stereoisomers resulted in decreased SAM levels after 24 h incubation, we noted that the difference between them was not large (within 20% of each other). This again may stem from temporal effects, which result in similar observations for the different isomers at longer experiment times (24 h).

We were curious as to whether the methionine restriction imparted by compound **1** also affected other metabolites beyond polyamines that are downstream of methionine and SAM. Therefore, in a separate experiment, we also measured both SAM and *S*-adenosylhomocysteine (SAH) levels in L3.6pl cells treated with RR and RS isomers of **1** (6 µM, 24 h) by LC-MS and showed the expected decrease in both SAM and SAH (see Table S1 in the Supplementary Materials). Additional rescue experiments demonstrated that exogenous SAM (100 µM) was unable to rescue L3.6pl cells treated with **1** (RS, 6 µM) after 24 h of incubation at 37 °C (Figure S4). This observation may in part be explained by the poor cellular import of SAM by mammalian cells [27]. Nevertheless, the observed reduced SAM and SAH pools are consistent with the methionine decrease induced by **1**, which is a cell stress that should also stimulate autophagy. To investigate this possibility, we next looked at a lipid pathway involving the classic autophagy marker LC3-II.

### 2.6. Autophagy Studies

Methionine restriction (MR) is a powerful way to alter cell metabolism and we envisioned that compound **1** may provide a unique way to pharmacologically control MR in a dose-dependent fashion. For example, polyamine biosynthesis relies on the SAM pools derived from methionine for the synthesis of spermidine and spermine. We previously demonstrated that polyamine metabolism was, indeed, directly affected by **1**, where **1** (RS) resulted in decreased intracellular spermidine and spermine pools and decreased growth of L3.6pl cells in a dose-dependent fashion [10]. While high levels of **1** were shown to dramatically deplete intracellular methionine stores and cause L3.6pl growth inhibition [10], we were interested in how cells would respond to lower doses of **1**. Could mild MR induced by **1** shunt cells to autophagy as a cell survival mechanism? Such a mode of action would be exciting because MR and autophagy have been linked to increased longevity in numerous organisms [3,4,5,6,7,9,28].

Intrigued by this opportunity to control intracellular methionine levels with a small molecule, we explored whether modest methionine restriction imparted by compound **1** could induce autophagy. This is theoretically possible because an autophagy pathway exists that is mediated by SAM levels and phosphatidylethanolamine (PE). Indeed, a recent study by Ye et al. demonstrated that PE is a major consumer of SAM in yeast [29]. The conversion of PE to phosphatidylcholine (PC) requires the donation of three methyl groups from three molecules of SAM [29]. When SAM levels are low, there are fewer SAM molecules available to convert PE to PC, and PE levels should theoretically increase. However, the formation of the autophagosome lipoprotein LC3-II requires the conjugation of PE to the LC3-I protein, a process required for autophagy. Therefore, the question remained what would happen to PE levels in the presence of a methionine restriction agent like **1**? Would PE levels increase or be shunted by the formation of the PE-LC3-I conjugate (i.e., LC3-II) to facilitate autophagy?

Using lipidomic methods and LC-MS, we determined the levels of phosphatidylethanolamine (PE), mono-methyl PE (MMPE), dimethyl PE (DMPE), and phosphatidylcholine (PC) in L3.6pl cells treated with 6 µM of each isomer of **1** for 24 h and compared these with untreated cells (or cells treated with JPH203, a known LAT1 inhibitor). As shown in Table 2, all isomers of **1** showed a modest decrease in PE pools ranging from 12.5–29.2% with little change in PC levels. Interestingly, the RR isomer was the most effective isomer and resulted in the most significant decrease in PE (−29.2%) and DMPE (−30.6%) pools. In contrast, JPH203 provided a control compound that inhibited methionine import but as shown in Table 2 did not significantly perturb intracellular levels of these lipids.

Having confirmed that PE pools were reduced, we next investigated whether they were shunted to the autophagy pathway and looked specifically for induced LC3-II expression, a gold standard for autophagy induction, following treatment with two different concentrations of compound **1**.

**Figure 5:**
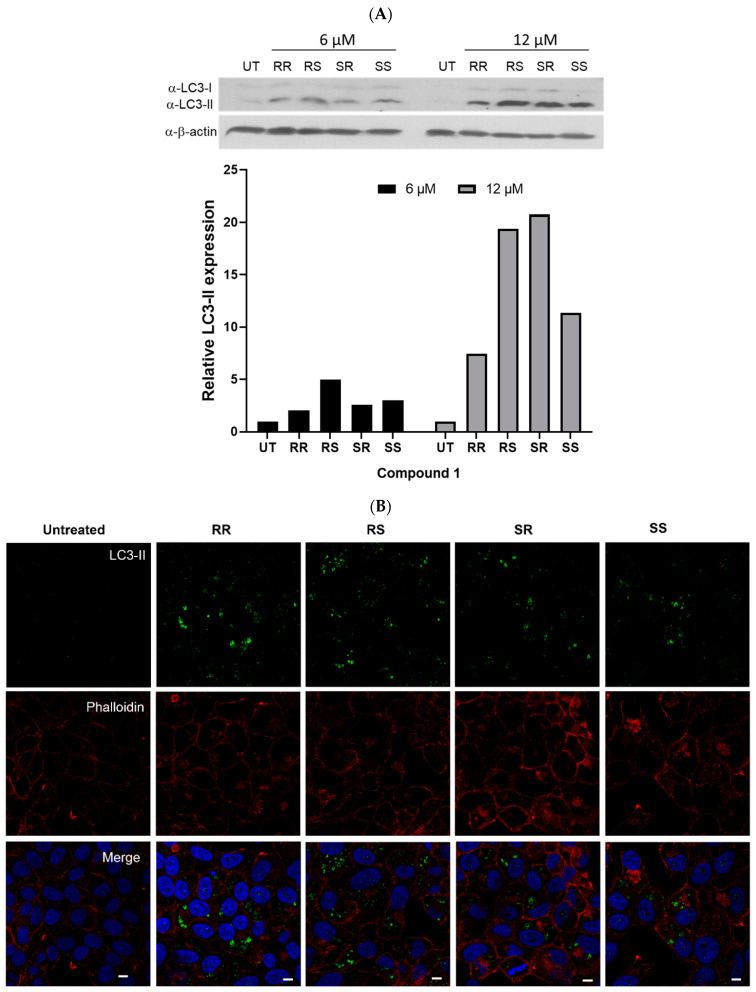
(**A**) Western blot showing induction and quantification of LC3-II protein in L3.6pl cells treated with each of the four isomers at either 6 µM or 12 µM of compound **1**. β-actin was used as a loading control. The quantification graph shows the relative expression of the LC3-II protein normalized to untreated in each group. Experiments were performed five times as single experiments and a representative image is shown in (**A**). (**B**) Representative confocal images showing LC3-II-positive puncta in cells treated with each of the four isomers at 6 µM. LC3-II protein is shown in green, phalloidin (actin stain) in red, and DAPI-stained nuclei are shown in blue. Scale bars shown in white represent 5 µm and all panels were taken at the same magnification. Original Blot Images can be found in the Supplementary Materials.

As shown in Figure 5A, LC3-II protein levels were increased following a 24 h treatment with either 6 µM or 12 µM of each isomer of **1**. We observed a robust dose-dependent induction of LC3-II protein with 12 µM treatment of each isomer compared with the untreated control. In addition, we also observed an increased number of LC3-II-positive puncta in cells treated with each of the four isomers by confocal microscopy at 6 µM (Figure 5B).

Having shown that LC3-II was induced by **1**, we investigated whether this occurred via lysosomal inhibition. This is important because tertiary amines are known to enter and inhibit lysosomal fusion/function resulting in decreased protein degradation. Therefore, our compounds may act by entering the lysosome, disrupting its function and causing a build-up of basal LC3-II lipoprotein, not via autophagy induction, but via inhibited LC3-II degradation [30,31]. To test this possibility, we used a published chloroquine (CQ) assay [32,33] to assess autophagic flux in the presence and absence of compound **1**.

**Figure 6:**
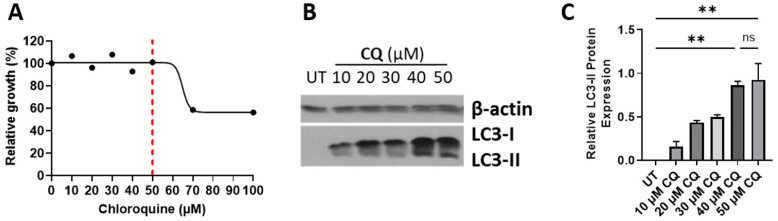
Chloroquine (CQ) mediated growth inhibition and protein studies showing a selection of the 50 μM dose. (**A**) Chloroquine induced growth inhibition in L3.6pl cells after 24 h of incubation. (**B**,**C**) Representative Western blot image showing induction and quantification of LC3-II protein in L3.6pl cells treated with different CQ doses. β-actin was used as a protein loading control. At least 3 independent experiments were performed. Data are presented as mean ± SEM. ** *p* ≤ 0.01. ns = not significant. Original Blot Images can be found in the Supplementary Materials.

CQ prevents the autophagosome and lysosome fusion step [32] and allows one to assess the lysosomal contribution to intracellular LC3-II levels [34]. LC3-II levels are expected to rise in the presence of increasing CQ concentration [32]. To select the optimal concentration for this assay, we first determined the growth inhibition curve of CQ in L3.6pl cells after a 24 h incubation (Figure 6A). This information allowed us to determine the maximum tolerated concentration (MTC) of CQ (50 μM), which provides >90% relative growth compared with the untreated control. We note that other groups have found that concentrations of CQ above 50 μM were growth inhibitory to other cell lines [34]. Western blots were then performed to investigate LC3-II expression at non-toxic doses of CQ ranging from 10 μM to 50 μM (Figure 6B). Figure 6C demonstrated that LC3-II levels are increased by CQ in a dose-dependent manner as expected. This effect, however, plateaued in the 40–50 µM CQ range (Figure 6C).

**Figure 7:**
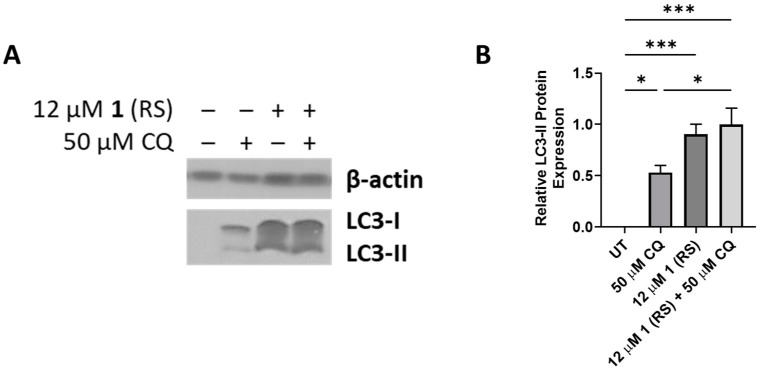
Protein studies consistent with increased induction of LC3-II with lysosomal blocking by CQ. (**A**,**B**) Representative Western blot image showing induction and quantification of LC3-II protein in L3.6pl cells treated with compound **1** (RS) at 12 μM and CQ at 50 μM, and the combined treatment over 24 h. β-actin was used as a protein loading control. At least 3 independent experiments were performed. Data are presented as mean ± SEM. * *p* ≤ 0.05, *** *p* ≤ 0.001. Original Blot Images can be found in the Supplementary Materials.

Next, we investigated how LC3-II expression changes in the presence and absence of CQ (50 µM) and **1** (Figure 7). The (RS) isomer of **1** at 12 μM was selected for this assay because it resulted in significant induction of LC3-II in preliminary experiments. As shown in Figure 7, both chloroquine and **1** induced LC3-II expression as expected. However, the combination of chloroquine and **1** gave a statistically significant higher expression of LC3-II when compared with CQ alone (*p*-value ≤ 0.05) and untreated (*p*-value ≤ 0.001). This result demonstrated that **1** can induce LC3-II production even when the lysosomal processing of LC3-II is disrupted by CQ. In summary, the CQ assay results are consistent with compound **1** inducing LC3-II expression/autophagy by a non-lysosomotropic mechanism.

In terms of the effect of the **1** + CQ combination on cell growth, we observed the expected synergism in growth inhibition resulting from the dual stress of lysosomal blockade (CQ: 25 µM) and LAT1 efflux agonism by **1** (RS) (6 µM; Figure S5). This effect became less apparent, however, at higher concentrations of **1** (12 µM) and CQ (50 µM) as the higher concentration of **1** (RS) led to significant growth inhibition further limiting the ‘response window’ of the assay (Figure S5).

In summary, all four isomers of **1** were shown to inhibit the growth of L3.6pl cells with similar IC_50_ values after a 24 h incubation period and were able to induce autophagy in cells via a non-lysosomotropic pathway as indicated by increased LC3-II expression in the presence of CQ.

## 3. Conclusions

**Figure 8:**
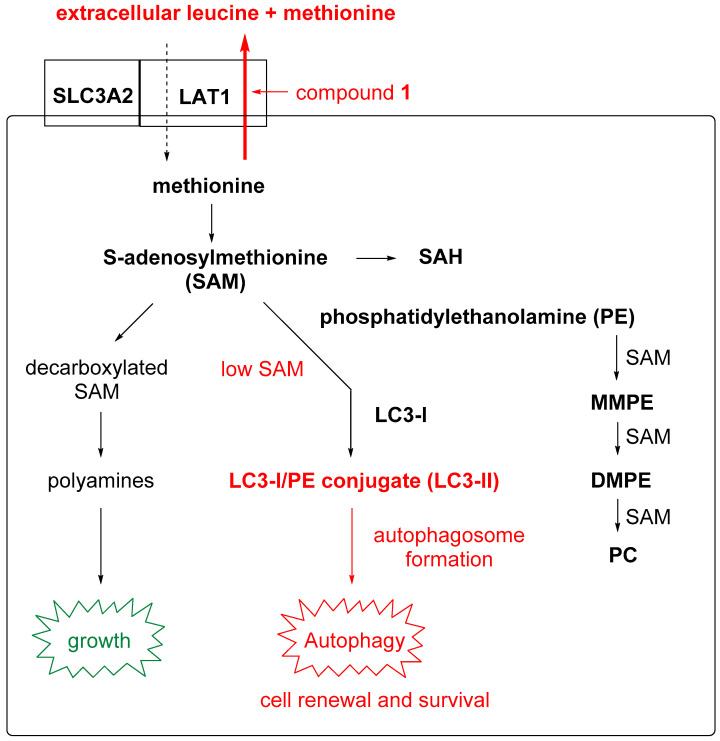
The interplay of methionine transport, polyamine metabolism, and lipid signaling for autophagosome formation via LC3-II and autophagy induction. Abbreviations: DMPE: dimethyl-phosphatidylethanolamine, LAT1: large amino acid transporter 1, MMPE: mono-methyl-phosphatidylethanolamine, PE: phosphatidylethanolamine, PC: phosphatidylcholine, SAH: *S*-adenosylhomocysteine, SAM: *S*-adenosylmethionine, and SLC3A2: membrane anchoring protein. LC3-I is a protein and LC3-II is a lipoprotein, wherein PE has been covalently attached to LC3-I. We note that the autophagic recycling of proteins can provide alternative sources of methionine for the cell.

These experiments allow us now to propose a mechanism of action for **1**. Our data support the mechanism where the methionine efflux induced by **1** lowers intracellular methionine, SAM, and polyamine levels and induces autophagy via the SAM-driven lipid pathway suggested by Ye et al. (Figure 8) [29]. Our work suggests that as methionine and SAM pools become limited, the scarcity of available methyl sources shunts PE pools toward lipidation of cytosolic LC3-I to form the autophagosome lipoprotein LC3-II (a PE/LC3-I conjugate). LC3-II is then recruited to the autophagosomal membranes, which, in turn, enables the autophagic recycling of proteins. This reshuffling of methylated and non-methylated lipids presumably occurs to ultimately reclaim methionine (Figure 8) and facilitate cell survival under conditions of MR.

While this report provides strong support for a lipid pathway for autophagy induction by **1**, there are many questions left to be answered and several future directions to pursue. For example, are all markers of autophagy upregulated or is there a specific autophagy marker profile associated with **1**? We have begun to investigate the list of known autophagy markers. For example, we found no significant changes occurred in ATG-7 and a modest reduction in ATG-5 expression was observed in the presence of **1** (see Figure S2 in the Supplementary Materials). While, a priori, one may have expected these ATG markers to increase, other work suggests that different markers of autophagy (mTOR, ULK 1/2, ATG13, and FIP200) may be more relevant to starvation-induced autophagy processes in human cells [35]. These markers will be the subject of future work along with an investigation of the expected concomitant effect of leucine restriction on mTOR signaling.

In closing, targeting LAT1 provides another method to reduce intracellular polyamines beyond the known biosynthetic inhibitors like difluoromethylornithine (DFMO) and polyamine depletion strategies, which in general have provided beneficial outcomes in several cancer models [36,37,38,39,40]. LAT1 efflux agonists provide a new way to regulate cancer cell growth via reduced SAM pools.

## 4. Experimental

Chemical reagents were purchased from commercial sources and used without further purification. A known LAT1 inhibitor, JPH203, was purchased from (MedKoo Biosciences, Morrisville, NC, USA). All solvents were of analytical grade and used without further purification. All reactions were performed under atmospheric pressure unless otherwise noted. ^1^H NMR spectra were recorded at either 500 or 400 MHz, while ^13^C NMR spectra were recorded at either 125 or 100 MHz, respectively. TLC solvent systems are represented as volume percentages. All compounds that were utilized for in vitro studies passed elemental analyses and were ≥95% pure.

### 4.1. Molecular Modeling

The four isomers of **1** (RR, RS, SS, and SR) were separately drawn in ChemDraw Prime 16.0 (PerkinElmer Informatics, Waltham, MA, USA; ver. 21.0.0.28) with ACS1996 document settings applied. Each structure was imported into Chem 3D Pro 12.0 (PerkinElmer Informatics) and underwent MM2 to minimize steric interactions and saved as pdb files. These were then imported into PyRx (Version 0.8; https://pyrx.sourceforge.io/, accessed 28 February 2024). The LAT1 protein (PDB ID: 6irs, accessed 31 May 2019) was downloaded from the Protein Databank (https://www.rcsb.org/, accessed 28 February 2024) and the four isomers were docked to this protein using the AutoDock Vina tool within PyRx [41,42]. The binding affinity of each isomer to LAT1 was determined in the units of kcal/mol. The docking process allows the ligand to flex whereas the protein target remains fixed. This allows the molecule to search the surface of the target protein and form binding interactions, which are ranked according to their relative binding affinity. The lowest energy binding event for each isomer of **1** was then rendered in Figure 3 using PyMol (version 1.3, Schrödinger, New York, NY, USA, 2010).

### 4.2. Biological Studies

L3.6pl cells were grown in RPMI 1640 medium with the addition of 10% fetal bovine serum and 1% penicillin/streptomycin and grown at 37 °C under a humidified 5% CO_2_ atmosphere. All cells were seeded at 1000 cells/mL for cytotoxicity studies, while L3.6pl cells were seeded at 100,000 cells/mL for studies involving L-leucine [4,5-^3^H] efflux assays in 24 well plates.

### 4.3. IC_50_ Determination

Cell viability was assessed in sterile 96-well plates (Costar 3599, Corning, Glendale, AZ, USA) using L3.6pl cells. Drug solutions (10 μL/well) of the appropriate concentrations were added after overnight incubation of the cell suspension (90 μL/well suspension in media). After incubation with the drug for 24 h, viability was measured through a metabolic assay measuring formazan formation from 3-(4,5-dimethylthiazol-2-yl)-5-(3-carboxymethoxyphenyl)-2-(4-sulphenyl)-2H-tetrazolium (MTS) by absorbance (490 nm) measurements using a Biotek SynergyMx microplate reader. The IC_50_ value was determined as the concentration of compound needed to inhibit 50% of the relative new growth.

### 4.4. Polyamine Level Determination

Intracellular polyamine levels were determined using a published *N*-dansylation HPLC protocol [10].

### 4.5. Lipidomics Procedure

Each suspension of L3.6pl cells (1 mL) was seeded into a 10 cm dish (at a density of 1.5 million cells/dish) in 9 mL of complete media (RPMI-1640 media with 10% fetal bovine serum and 1% penicillin/streptomycin antibiotic cocktail). The media contained 10 µL of aminoguanidine (250 mM, 250 µM final concentration). The cells were then incubated for 24 h at 37 °C in a 5% CO_2_ incubator. Cells were treated with either 100 µL of each isomer of compound **1** (600 µM stock in phosphate-buffered saline (PBS), 6 µM final concentration) or JPH203 (10 mM stock, 100 µM final concentration) or PBS (1×) and incubated for 24 h at 37 °C in a 5% CO_2_ incubator. The respective supernatant was aspirated off and the cells were washed twice with cold PBS (5 mL). The PBS was then removed and cells were trypsinized and the cell suspension was spun in a centrifuge (1000 RPM, 4 min, 25 °C). The supernatant was removed from the resulting cell pellet and the pellet was washed by re-suspending in PBS (5 mL) and spun down again to re-form the pellet. The supernatant was removed and the remaining pellet was collected and stored in a −80 °C freezer until quantification of phospholipid populations by mass spectrometry and shotgun lipidomics.

Multidimensional mass spectrometry-based shotgun lipidomic analysis of the phospholipids in the L3.6pl samples was performed as described [43,44]. In brief, remixed internal standard (IS) was added to each cell homogenate for quantitation of each lipid species and then normalized with the respective protein content (per mg protein). Lipids were extracted using a modified Bligh and Dyer procedure [45], and then each lipid extract was reconstituted in 1:1 (*v*/*v*) chloroform/methanol at a volume of 400 µL/mg of protein. The phospholipids were derivatized and quantified using a previously reported method [46]. Briefly described, the respective lipid extracts (equivalent to 0.05–0.1 mg of protein content) were each transferred into a borosilicate glass vial. Then, CD_3_I and saturated NH_4_OH were added to derivatize the lipids of interest. Followed by a purification process, the lipid mixture was resuspended in CHCl_3_/MeOH (1:1, *v*/*v*) for MS analysis. For shotgun lipidomics, the lipid extracts were diluted to a final concentration of ~500 fmol total lipids per µL. Mass spectrometric analysis was performed on a triple quadrupole mass spectrometer (TSQ Altis, Thermo Fisher Scientific, San Jose, CA, USA) equipped with an automated nanospray device (TriVersa NanoMate, Advion Bioscience Ltd., Ithaca, NY, USA) as described in [47]. The identification and quantification of lipid species were performed using an automated software program as previously described [48,49]. Data processing (ion peak selection, baseline correction, data transfer, peak intensity comparison, and quantitation) was performed as described in [49].

### 4.6. Determination of Intracellular SAM Levels

L3.6pl cells were seeded at a concentration of 3 × 10^6^ cells per 10 cm dish. Cells were treated with each isomer of **1** (6 or 12 μM) for 24 h at 37 °C in a 5% CO_2_ incubator. Respective cell pellets were prepared as described above. The resulting pellets were resuspended in 150 µL of NaOAc (18 mM) and 100 µL of perchloric acid (PCA) buffer (0.2 M HClO_4_/1 M NaCl). Cells were homogenized by sonication and the cell suspension was centrifuged at 4000 rpm for 10 min. The resulting supernatants (lysates) were collected and the respective pellets were stored for protein analysis using the bicinchoninic acid (BCA) kit. Note: a portion of the supernatant (20 µL) obtained from the 6 µM experiments with RR and RS isomers of **1** were used for LC-MS analysis of SAM and SAH levels (see Supplementary Materials). The remaining supernatant (~250 µL) was transferred into a 4 mL glass vial for further SAM derivatization via a modified protocol of Birsan et al. [50]. To each supernatant, 100 µL of NaOAc (18 mM) and 50 µL of choloroacetaldehyde (45% *w*/*v*) were added and the resulting mixture was incubated at 60 °C for 2 h to obtain the corresponding fluorescent SAM, N^6^-ethano derivatives. At the end of incubation, water (600 µL) and methanol (500 µL) were added and vortexed for 15 s to mix. The samples were filtered using 0.45 µm nylon syringe filters and prepared for HPLC analysis. Purified SAM from Sigma Aldrich was derivatized using the above-described process and used to generate a standard curve from which intracellular SAM content was determined for each treatment. Derivative samples (100 µL) were immediately loaded onto a Shimadzu High-Performance Liquid Chromatograph (HPLC). A mobile phase consisting of KH_2_PO_4_ (50 mM) in 1-heptanesulphonic acid (10 mM) buffer (pH 4.3) in pump A and methanol in pump B was used. The SAM N^6^-ethano derivatives were eluted using isocratic elution with a flow rate of 1 mL/min (buffer ratio of 0.9 pump A: 0.1 pump B) through a C_18_ reverse-phase column (5 µm, 250 × 4.6 mm). The SAM N^6^-ethano derivative reproducibly eluted ~11.8 min and the area under the curve was recorded and compared with the calibration curve generated with authentic SAM. The data were normalized over protein (mg) and reported as a relative % SAM compared with an untreated control. All the experimental conditions were performed in triplicate.

### 4.7. Immunoblotting

Cell extracts were prepared in modified radioimmunoprecipitation assay (RIPA) buffer (20 mM HEPES, pH 7.0, 150 mM NaCl, 1 mM EDTA, 1% NP-40, 1% deoxycholate, and 0.1% sodium dodecyl sulfate [SDS]) containing complete protease inhibitor cocktail (Roche, Mannheim, Germany) and PhosSTOP phosphatase inhibitor cocktail (Roche). Protein concentrations were determined using the BCA protein assay kit (Pierce, Rockford, IL, USA) according to the manufacturer’s instructions. For each sample, 50 μg of protein was separated by SDS–polyacrylamide gel electrophoresis (PAGE) and electro-transferred onto nitrocellulose membranes (BioRad, Hercules, CA, USA). Proteins were detected with different primary antibodies followed by horseradish peroxidase-conjugated secondary antibodies. Primary antibodies used were anti-ATG7 (B-9) and anti-ATG-5 (C-1) from Santa Cruz Biotechnology, anti-LC3-I/II from Millipore, and anti-β-actin from Abcam (Waltham, MA, USA). Proteins were detected on the membrane with the chemiluminescent reagent SuperSignal West Dura (Life Technologies, Carlsbad, CA, USA).

Cells were seeded on coverslips in 24-well plates at a concentration of 40,000–50,000 cells with 250 μM of aminoguanidine in 500 μL of media per well. Before fixation, the media was removed followed by three 1× PBS washes (5 min). Cells were then fixed with 4% PFA for 20 min at room temperature. Following washes with PBS, the cells were permeabilized with 0.1% Triton X-100 in PBS for 15 min at room temperature and then blocked with 10% goat serum in PBS-T (0.1% Tween-20 in PBS) for 30 min at room temperature. Cells were then stained with primary antibody in blocking solution at 4 °C overnight in a humidified chamber followed by secondary antibody for 1 h at room temperature. Coverslips were mounted onto slides using a drop of mounting media with DAPI (Southern Biotech, Birmingham, AL, USA).

### 4.8. Radiolabeled Leucine Efflux Assay

^3^H-leucine efflux experiments were performed according to the protocol developed by Hafliger et al. with the following changes [14]. Briefly, L3.6pl cells (100,000 cells in 270 μL) were preloaded with 30 μL of 12 µM stock of L-[^3^H]leucine (79 Ci/mmol) to give a final concentration of 1.2 µM ^3^H-leucine in each well. Cells were preloaded for 5 min at 37 °C, then washed three times with cold Na^+^-free HBSS. JPH-203 stock solutions were made in 3% DMSO and compound **1** was dissolved in PBS. For experiments with one additive (e.g., **1** alone), 270 µL of Na^+^-free HBSS at rt was added followed by the addition of the single agent (e.g., compound **1** only) as a 10× stock solution (30 µL). For experiments with two additives (e.g., JPH-203 + unlabeled leucine), 240 µL of Na^+^-free HBSS at rt was added followed by the addition of each agent (e.g., JPH-203 or unlabeled Leu) as a 10× stock solution (30 µL). The total volume in these assays was always 300 µL and either DMSO (30 µL) or PBS (30 µL) was added to the control wells so that the solvent composition was identical in all wells. The compounds and cells were incubated for 30 min at 37 °C. The supernatant was then collected (300 µL) and mixed with scintillation fluid (Scintiverse™ BD Cocktail, 2 mL, Fisher Scientific, Fairlawn, NJ, USA) and radioactivity was measured (Beckman Coulter LS 6500 Multi-Purpose Scintillation Counter, Brea, CA, USA). The cells were then washed three times with cold Na^+^-free HBSS (4 °C) and lysed to give ~300 µL of cell lysate. The lysate (300 µL) was mixed with scintillation fluid (2 mL) and radioactivity was counted. Relative efflux was expressed as percentage radioactivity = 100% × (radioactivity of medium)/(radioactivity of the medium + radioactivity of the cells).

### 4.9. Chloroquine (CQ) Growth Inhibition Assay

Relative cell growth was assessed in sterile 96-well plates (Costar 3599, Corning) using L3.6pl cells. Chloroquine (CQ) solutions (10 μL/well) of the appropriate concentrations were added after overnight incubation of the cell suspension (90 μL/well suspension in media). After incubation with CQ for 24 h, viability was measured through the MTS assay measuring formazan formation from 3-(4,5-dimethylthiazol-2-yl)-5-(3-carboxymethoxyphenyl)-2-(4-sulphenyl)-2H-tetrazolium (MTS) by absorbance (490 nm) measurements using a Biotek SynergyMx microplate reader. The MTC value was determined as the highest concentration of compound that allows for greater than 90% of the relative growth compared with the untreated control.

### 4.10. Western Blot

L3.6pl cells were treated with either the PBS vehicle (untreated), CQ, and/or **1** (RS) for the stated time period. The respective cells were then washed with PBS and the cell extracts were prepared after 24 h of incubation in modified radioimmunoprecipitation assay (RIPA) buffer (20 mM HEPES, pH 7.0, 150 mM NaCl, 1 mM EDTA, 1% NP-40, 1% deoxycholate, and 0.1% sodium dodecyl sulfate [SDS]) containing complete protease inhibitor cocktail (Roche) and PhosSTOP phosphatase inhibitor cocktail (Roche). Protein concentrations were determined using the bicinchoninic acid (BCA) protein assay kit (Pierce) according to the manufacturer’s instructions. For each sample, 30 μg of protein was separated by SDS–polyacrylamide gel electrophoresis (PAGE) and electro-transferred via the Trans-Blot Turbo Transfer System (BioRad) onto polyvinylidene difluoride (PVDF) membranes (BioRad). Proteins were detected with their respective primary antibodies followed by horseradish peroxidase-conjugated secondary antibodies. Primary antibodies used were anti-LC3-I/II from Millipore and anti-β-actin from Abcam at 1:250 and 1:10,000 dilutions, respectively. Proteins were detected on the membrane via the chemiluminescent reagent SuperSignal West Pico PLUS (ThermoScientific, Waltham, MA, USA). Western blot densitometry in LI-COR Image Studio Lite version 4.0 was used to quantify LC3-II protein levels. Western blots were run in triplicate and the data were presented as the mean of the triplicate experiment. Raw data are provided in the Supplementary Materials.

### 4.11. Statistical Analysis

Experimental data were managed in Excel 2019 and GraphPad Prism version 9.0.0 was used to perform data analysis. One-way ANOVA was used to evaluate LC3-II protein quantification from the Western blot data. The *p*-value was set to <0.05 to show the statistical significance of the data.

## Figures and Tables

**Table 1:** Growth inhibition results of all four stereoisomers of **1** in L3.6pl cells. ^a^

Compound	24 h IC50 (µM)—1000 Cells	24 h IC50 (µM)—3000 Cells
**1** (RR)	3.99 ± 0.04	5.14 ± 0.05
**1** (RS)	5.43 ± 0.16	7.35 ± 0.18
**1** (SR)	3.64 ± 0.20	4.13 ± 0.05
**1** (SS)	3.32 ± 0.07	4.76 ± 0.14

^a^ The stereochemistry of the exocyclic chiral center is listed first and the stereochemistry of the piperazine ring-associated chiral center is listed second. For example, the RS isomer is built from *R*-cyclohexylalanine methyl ester and *S*-leucine methyl ester. Note: similar growth inhibition was seen at 2000 cells per well loadings as shown in Table S2.

**Table 2:** LC-MS phospholipid analysis of treated and untreated L3.6pl cells ^a,b^.

Phospholipid	Control	JPH203	RR	RS	SR	SS
**Phosphatidyl ethanolamine** (PE)	185.18 ± 5.32	177.36 ± 1.96	131.04 ± 8.89	162.08 ± 17.84	149.89 ± 10.21	150.7 ± 8.98
**% change vs. control**	0%	−4.2%	−29.2%	−12.5%	−19.1%	−18.6%
**Mono-methylated PE** (MMPE)	0.86 ± 0.15	0.85 ± 0.01	0.85 ± 0.06	0.91 ± 0.09	0.84 ± 0.21	0.94 ± 0.08
**% change vs. control**	0%	−1.1%	−1.1%	+5.8%	−2.3%	+9.3%
**Di-methylated PE** (DMPE)	1.57 ± 0.17	1.39 ± 0.19	1.09 ± 0.19	1.39 ± 0.12	1.43 ± 0.12	1.36 ± 0.14
**% change vs. control**	0%	−11.4%	−30.6%	−11.4%	−8.9%	−13.3%
**Phosphatidylcholine** (PC)	137.39 ± 3.18	133.26 ± 13.51	126.45 ± 10.48	141.53 ± 1.53	135.20 ± 5.47	145.59 ± 4.68
**% change vs. control**	0%	−3.0%	−8.0%	+3.0%	−1.6%	+6.0%
**Total phospholipids measured**	325.0	312.9	259.4	305.9	287.4	298.7
**% change vs. control**	0%	−3.7%	−20.1%	−5.9%	−11.5%	−8.1%

^a^ L3.6pl cells were plated and treated for 24 h at 37 °C in the presence and absence of either JPH203 (100 µM) or the respective isomer of compound **1** (6 µM) or PBS. Experiments were performed in triplicate and the data are listed in nmol phospholipid per mg protein ± standard deviation; ^b^ the % change is relative to the untreated control.

## Data Availability

The data is available in the extensive Supplementary Materials provided or via email contact with the communicating author (O.P.IV).

## Supplementary Materials

The following supporting information can be downloaded at: https://www.mdpi.com/article/10.3390/biom14030326/s1, Figure S1: Structures of the LAT1 inhibitor JPH203, BCH, and D-phenylalanine; Table S1: S-adenosylhomocysteine (SAH) and S-adenosylmethionine (SAM) levels as measured by LC-MS in L3.6pl cells treated with different isomers of **1** at 6 µM for 24h; LC-MS/MS analysis for SAM and SAH quantification; Figure S2. Western blot image and quantification of ATG-7 and ATG-5; Synthesis and characterization of the four diastereomers of **1**; Circular Dichroism spectra of the four diastereomers of **1**; Figure S3. Assessment of racemization in compounds **9**, **10**, and **1**; ^1^H and ^13^C NMR (CDCl_3_) spectra for the RR, SS, and SR isomers of **1**; ^1^H and ^13^C NMR (CDCl_3_) spectra for **4** (R-isomer) and ^1^H NMR of **4** (S); ^1^H and ^13^C NMR (DMSO-d_6_) spectra for the R and S isomers of **5**; ^1^H and ^13^C NMR (CDCl_3_) spectra for the RR, SS, and SR isomers of **6**–**10**; Table S2. Compound **1** (RS) and Compound **1** (RR) studies showing an increase in potency at longer incubation times; Figure S4. Investigation showing the inability of exogenous *S*-Adenosylmethionine (SAM) to rescue L3.6pl cells treated with Compound **1** (RS); Figure S5. Investigation of Chloroquine (CQ) in combination with Compound **1** (RS) in L3.6pl pancreatic cancer cells; Raw data for Western blots associated with experiments.
