# Supplementary material for: The Development of LAT1 Efflux Agonists as Mechanistic Probes of Cellular Amino Acid Stress
Source: Biomolecules. 2024 Mar 9;14(3):326. doi: 10.3390/biom14030326 (PMC10968016; doi:10.3390/biom14030326)

Article

The Development of LAT1 Efflux Agonists as Mechanistic Probes of Cellular Amino Acid Stress

Vandana Sekhar †, Houssine Ikhlef †, Alexandra Bunea, Viet S. Nguyen, Johan Joo, Mukund P. Tantak, Holly Moots and Otto Phanstiel IV *

Department of Medical Education, College of Medicine, University of Central Florida, 12722 Research Parkway, Orlando, FL 32826, USA

* Correspondence: otto.phanstiel@ucf.edu; Tel.: +1-407-823-6545; Fax: +1-407-384-2062

† These authors contributed equally to this work.

Table of Contents: **Page(s)**

Figure S1. Structures of the LAT1 inhibitor JPH203, BCH, and D-phenylalanine S3

Table S1 and experimental for SAH and SAM levels in L3.6pl cells as measured by LCMS S4

Figure S2. Western blot image and quantification of ATG-7 and ATG-5 S6

Synthesis and characterization of the four diastereomers of **1** S7-S24

Circular Dichroism spectra of the four diastereomers of **1** S25-26

Figure S3. Assessment of racemization in compounds **9, 10**, and **1** S27-29

^{1}H and ^{13}C NMR ($CDCl_3$) spectra for the RR, SS, and SR isomers of **1** S30-35

^{1}H and ^{13}C NMR ($CDCl_3$) spectra for **4** (R-isomer) and ^{1}H NMR of **4** (S) S36-38

^{1}H and ^{13}C NMR (DMSO-d_6) spectra for the R and S isomers of **5** S39-42

^{1}H and ^{13}C NMR ($CDCl_3$) spectra for the RR, SS, and SR isomers of **6** S43-48

^{1}H and ^{13}C NMR (DMSO-d_6) spectra for the RR, SS, and SR isomers of **7** S49-54

^{1}H and ^{13}C NMR ($CDCl_3$) spectra for the RR, SS, and SR isomers of **8** S55-60

^{1}H and ^{13}C NMR ($CDCl_3$) spectra for the RR, SS, and SR isomers of **9** S61-66

^{1}H and ^{13}C NMR ($CDCl_3$) spectra for the RR, SS, and SR isomers of **10** S67-72

Table S2. Compound **1** (RS) and Compound **1** (RR) studies showing an increase in potency at longer incubation times S73

Figure S4. Investigation showing the inability of exogenous *S*-Adenosyl methionine (SAM) to rescue L3.6pl cells treated with Compound **1** (RS) S74

Figure S5. Investigation of Chloroquine (**CQ**) in combination with Compound **1** (RS) in L3.6pl pancreatic cancer cells S75

Raw data for Western blots associated with experiments S76-79

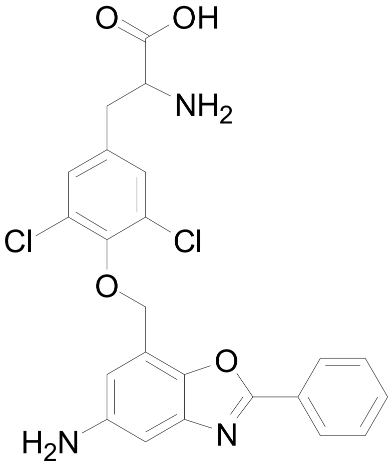

JPH203

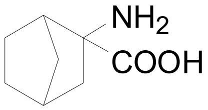

BCH

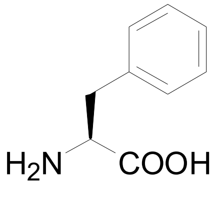

D-Phe

Figure S1. Structures of the LAT1 inhibitor **JPH203**, **BCH**, and **D-phenylalanine (D-Phe)**

Table S1. S-adenosylhomocysteine (SAH) and S-adenosylmethionine (SAM) levels as measured by LC-MS in L3.6pl cells treated with different isomers of **1** at 6 µM for 24h

Treatment	pmole of SAH in 20 uL sample	pmole SAM in 20 uL sample	mg protein in 20 uL sample	pmole SAH/mg protein	pmole SAM/mg protein
untreated	1.46 ± 0.95	119.5 ± 14.7	0.204	7.2	586.2
RR isomer of **1**	0.40 ± 0.30	45.7 ± 2.5	0.161	2.5	283.2
RS isomer of **1**	0.24 ± 0.06	63.5± 6.0	0.214	1.1	296.8

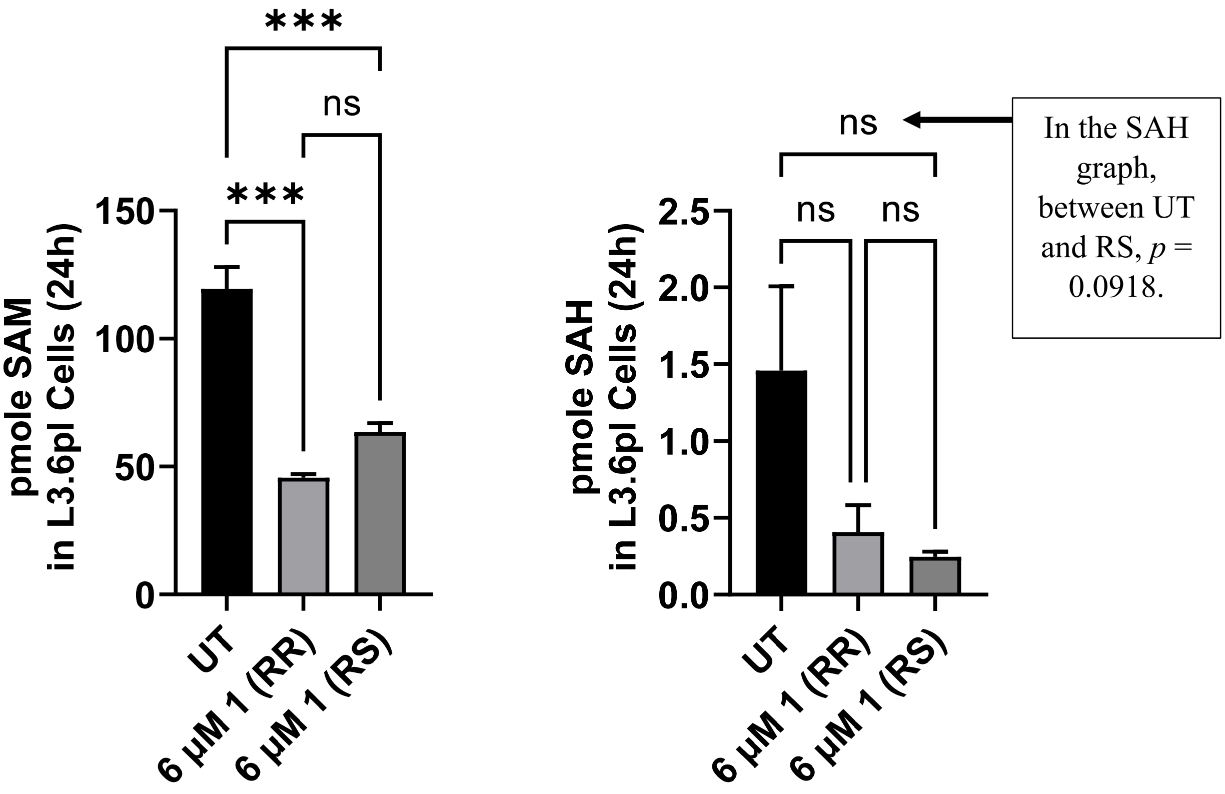

Experiments were performed in triplicate. Data are presented as mean ± SEM. *** $p < 0.001$.

LC-MS/MS analysis for SAM and SAH quantification. L3.6pl cells were seeded at a concentration of 3×10^6 cells per 10 cm dish. Cells were treated with the RR or RS isomer of **1** (6 µM) for 24 h at 37 °C in a 5% CO_2 incubator. The respective supernatant was aspirated off and the cells washed twice with ice cold PBS (5 mL). The PBS was then removed and cells were trypsinized and the cell suspension was spun in a centrifuge (1000 RPM, 4 minutes, 25 °C). The supernatant was removed from the resulting cell pellet and the pellet was washed by re-suspending in PBS (5 mL) and spun down again to re-form the pellet. The supernatant was removed, and the remaining pellet was collected and stored in a -80 °C freezer until quantification. As described in the Experimental

section for SAM level determination by HPLC, the resulting pellets were suspended in 150 µL of NaOAc (18 mM) and 100 µL of perchloric acid (PCA) buffer (0.2M $HClO_4$/1M NaCl). Cells were homogenized by sonication and the cell suspension was centrifuged at 4000 rpm for 10 minutes. The resulting supernatant was collected (~270 µL) and used for SAM analysis by HPLC or in this case 20 µL used for SAH and SAM levels by LCMS. The remaining pellets were used for protein analysis using the bicinchoninic acid (BCA) kit from Pierce as per manufacturer instructions to provide the mg of protein for each sample. A portion of the supernatant (20 µL) was used for LCMS analysis of SAM and SAH levels below.

SAM and SAH were quantified using mass spectrometry. A deuterated standard was used as an internal standard, where 1 pmol SAH-d_4 was added to each sample, followed by 100 µL of methanol for extraction. The mixture was vortexed and centrifuged at 10,000g for 5 min. The extracts were analyzed on an Acquity UPLC system (Waters, Milford, MA, United States) coupled to TSQ Quantum Access triple-quadrupole tandem mass spectrometer (Thermo Fisher Scientific, San Jose, CA, United States) with a heated electrospray (H-ESI) source in positive ion polarity. The mobile phase was a blend of solvent A (0.1% v/v acetic acid in water) and solvent B (0.1% v/v acetic acid in acetonitrile) on an Acquity UPLC BEH Amide column (2.1 × 150 mm, 1.7 µm) and an Acquity UPLC BEH Amide VanGuard column. Chromatographic separation was performed with following gradient program: 0 min (5% A, 95% B), 1 min (5% A, 95% B), 4 min (30% A, 70% B), 5 min (95% A, 5% B), 6 min (95% A, 5% B), 6.5 min (5% A, 95% B), 10 min (5% A, 95% B). The flow rate and column temperature were 0.3 mL/min and 60 °C, respectively. A sample injection volume of 10 µL was used for all injections and the overall run time was 10 min. The SAM, SAH, and SAH-d_4 were run in selected reaction monitoring (SRM) mode and precursor-to-product ion transitions m/z 399.1 → m/z 250.0, m/z 385.1 → m/z 136.1 and m/z 389.1 → m/z 136.1 were used for detection. The ESI source was operated with spray voltage = 3500 V, vaporizer temperature = 350 ◦C, sheath gas = 30 psi, auxiliary gas = 15 psi, and capillary temperature = 400 ◦C. Scan time was 0.2 s per SRM, and the scan width was m/z 0.01. Optimum collision energy and S-lenses conditions were determined for SAM, SAH and SAH-d_4 by using the auto-tune software for each analyte by post-column infusion of the individual compounds into a 50% A/50% B blend of the mobile phase being pumped at a flow rate of 0.3 mL/min. Data acquisition and processing were performed using Xcalibur software.

The LCMS analysis provided pmol of each analyte in the 20 uL of sample tested. We then normalized these values by dividing by the number of mg of protein in each 20 uL sample (i.e., the pmoles of SAM or SAH in 20 µL sample divided by the mg of protein in the 20 uL of sample). In this manner we could normalize the results in the above **Table S1**.

*Consistent with our HPLC analysis both the RR and RS isomers of **1** resulted in lower SAM levels. As shown in Table S1, a trend to lower SAH levels was also observed in the presence of **1**. We note that the low picomolar levels of the SAH analyte introduced significant error in the SAH measurements.*

A.

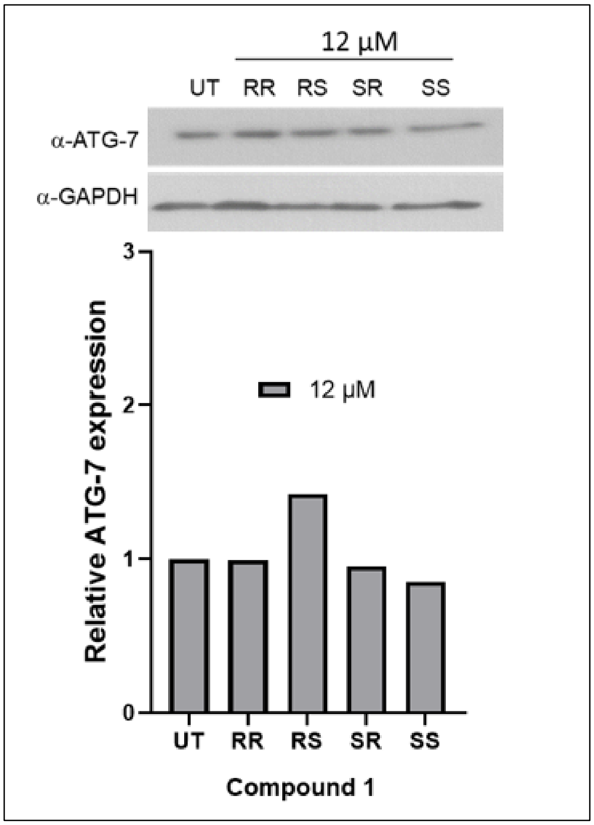

B.

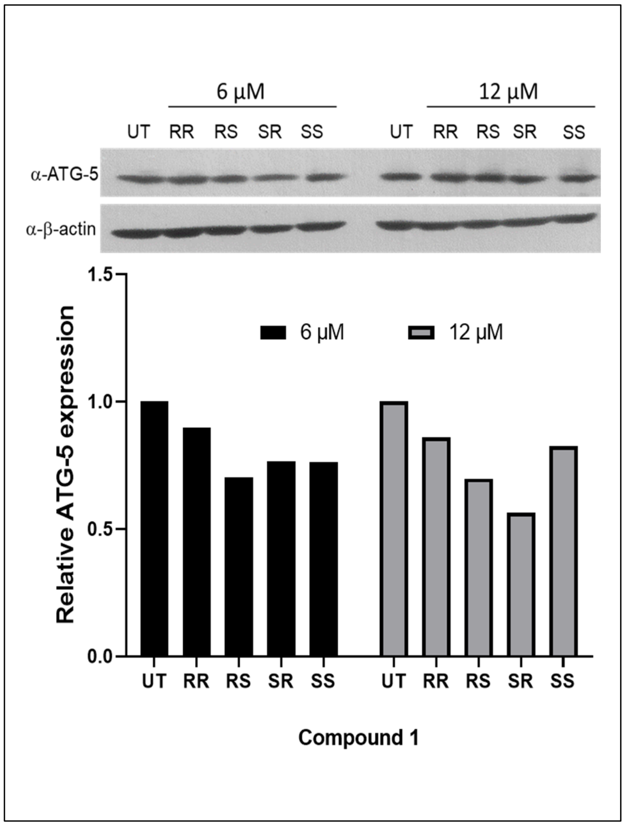

Figure S2. Representative Western blot showing expression and quantification of ATG-7 protein **(A)** and ATG-5 protein **(B)** respectively**,** in L3.6pl cells treated with each of the four isomers of compound **1** at the concentration indicated. β-actin was used as a loading control. The quantification shows the relative expression of the respective proteins normalized to untreated in each group. ATG-5 and ATG-7 Western blot experiments were run as singlets four times and three times, respectively. Representative images are shown. While no significant changes occurred in ATG-7, a modest reduction in ATG-5 expression was observed.

S3. Synthesis and Characterization. The synthesis of the R,S isomer of **1** has been reported.(9) The synthesis of the chiral intermediates and other isomers are described below.

Benzyl-(3-cyclohexyl-2-[3-isobutyl-4-(3-methyl-butyl)-piperazin-1-yl]-propyl)-amine (1-R,R): Borane-tetrahydrofuran complex (1M solution, 1.37 mL, 1.37 mmol, 8 equiv) was added to *N*-(3-Cyclohexyl-2-[5-isobutyl-4-(3-methyl-butyl)-2,3-dioxo-piperazin-1-yl]-propyl)-benzamide (**10 RR isomer**) (83 mg, 0.17 mmol, 1 equiv). The mixture was then heated to reflux and stirred for 5 days. The solution was then concentrated under reduced pressure to give a residue. The residue was taken up in a 10% HCl/MeOH solution (4 mL) and stirred for 1 day. The solution was concentrated under reduced pressure to give a residue which was taken up in 1M NaOH (pH 12), then extracted three times with DCM. The organic layer was separated, dried over anhydrous sodium sulfate, filtered, and concentrated under reduced pressure. The crude triamine free base was then purified by flash column chromatography (2% MeOH, 1% NH_4OH, in DCM) to yield pure triamine, **1 (RR isomer)** as a yellow oil (47 mg, 63%). **1 (RR isomer)**: ^{1}H-NMR (400 MHz, $CDCl_3$): δ 7.32 (m, 4H), 3.80 (m, 2H), 2.51 (m, 13H), 1.61 (m, 21H), 1.26 (m, 11H), 0.86 (m, 14H); ^{13}C-NMR (100 MHz, $CDCl_3$): δ 140.4, 128.3, 128.1, 126.8, 60.2, 53.9, 51.7, 49.6, 35.1, 34.3, 34.0, 33.0, 26.7, 26.5, 26.3, 26.1, 25.6, 24.1, 22.8, 22.6, 22.0. High resolution mass spectrum $[M + H]^+$: theory for $C_{12}H_{24}N_3$ 442.4161; found 442.4172. Anal Calcd $C_{29}H_{51}N_3$ 0.17 H_2O, Theory: C 77.77, H 11.55, N 9.38, Found: C 77.79, H 11.71, N 9.35. Note for long term storage, the free base of **1**(R,R) could be converted to its HCl salt. For example, the free base of **1**(RR; 39 mg, 0.09 mmol) was dissolved in absolute ethanol (2 mL) at 0°C. A 4 M HCl solution (4 mL) was slowly added to the free base solution. The solution

was stirred for 30 minutes and then concentrated. The resulting solid was taken up in water and concentrated to remove any remaining ethanol to give the amine hydrochloride salt of **1** (RR) (39 mg, 81% yield) as a crystalline solid.

Benzyl-(3-cyclohexyl-2-[3-isobutyl-4-(3-methyl-butyl)-piperazin-1-yl]-propyl)-amine (1; S,S isomer): Borane-tetrahydrofuran complex (1M solution, 14.7 mL, 14.7 mmol) was added via syringe to N-(3-Cyclohexyl-2-[5-isobutyl-4-(3-methyl-butyl)-2,3-dioxo-piperazin-1-yl]-propyl)-benzamide, (**10 SS isomer,** 890 mg, 1.84 mmol, 1 equiv) at rt. The mixture was then heated at reflux for 5 days. The reaction mixture was concentrated under reduced pressure to give a residue. A 10% concentrated HCl /methanol solution (20 mL) was then added at 0° C and stirred for 3 days. The mixture was concentrated under reduced pressure to give a residue. 1 M NaOH was added at 0°C (until pH 12) and the material was extracted three times with DCM. The organic layer was separated, dried over anhydrous sodium sulfate, filtered and concentrated to give the crude triamine (862 mg). The crude triamine was purified by flash chromatography (2% MeOH, 1% NH_4OH in DCM) to yield the triamine **1(S,S-isomer)** as a yellow oil (501 mg, 61% yield). **1(S,S)**: ^{1}H-NMR (400 MHz, $CDCl_3$): δ 7.32 (m, 3H), 7.23 (m, 1H), 3.78 (m, 2H), 2.50 (m, 12H), 1.61 (m, 7H), 1.26 (m, 8H), 0.88 (m, 14H); ^{13}C-NMR (100 MHz, $CDCl_3$): δ 140.6, 128.3, 128.1, 126.7, 60.3, 54.0, 51.7, 49.7, 35.2, 35.1, 34.3, 34.0, 33.0, 26.7, 26.5 26.3, 26.1, 25.6, 24.1, 22.9, 22.6, 22.0. High resolution mass spectrum $[M + H]^+$: theory for $C_{29}H_{52}N_3$: 442.4161; found 442.4164. Anal Calcd $C_{29}H_{51}N_3$ 0.30 H_2O Theory: C 76.97, H 11.49, N 9.29, Found: C 76.99, H 11.42, N 9.67. Alternatively, the free base could be converted to its HCl salt for long term storage. The free base of **1**(S,S) (49 mg 0.11 mmol) was dissolved in absolute ethanol (3 mL) at 0° C. A 4 M HCl solution (6 mL) was slowly added

to the free base solution. The solution was stirred for 30 minutes and then concentrated. The resulting solid was taken up in water and concentrated to remove any remaining ethanol to give the amine salt of **1(SS HCl salt)** (43 mg, 62%) as a crystalline solid.

Benzyl-(3-cyclohexyl-2-[3-isobutyl-4-(3-methyl-butyl)-piperazin-1-yl]-propyl)-amine (1; S,R isomer): Borane-tetrahydrofuran complex (1M solution, 9.6 mL, 9.6 mmol) was added via syringe to *N*-(3-Cyclohexyl-2-[5-isobutyl-4-(3-methyl-butyl)-2,3-dioxo-piperazin-1-yl]-propyl)-benzamide, **10 (SR isomer)**, (581 mg, 1.2 mmol) at rt. The mixture was then heated at reflux for 5 days. The reaction mixture was concentrated under reduced pressure to give a residue. A 10% concentrated HCl /methanol solution (20 mL) was then added at 0°C and stirred for 3 days. The mixture was concentrated under reduced pressure to give a residue. 1 M NaOH was added at 0°C until pH 12. The resulting material was then extracted three times with DCM. The organic layer was separated, dried over anhydrous sodium sulfate, filtered and concentrated to give the crude triamine (467 mg). The crude triamine was purified by flash chromatography (2% MeOH, 1% NH_4OH in DCM) to yield the pure triamine **1(S,R isomer)** as a yellow oil (233 mg, 43% yield). **1(S,R isomer)**: ^{1}H-NMR (400 MHz, $CDCl_3$): δ 7.32 (m, 4H), 7.25 (m, 1H), 3.79 (dd, 2H), 2.68 (m, 5H), 2.51 (m, 3H), 2.33 (m, 3H), 2.11 (m, 1H), 1.57 (m, 7H), 1.25 (m, 9H), 0.88 (m, 14H); ^{13}C-NMR (100 MHz, $CDCl_3$): δ 140.5, 128.4, 128.0, 126.8, 60.4, 54.0, 51.7, 49.7, 35.1, 34.2, 33.1, 26.7, 26.6, 26.3, 26.1, 25.6, 24.0, 22.9, 22.6, 22.0; High resolution mass spectrum $[M + H]^+$: theory for $C_{29}H_{52}N_3$: 442.4156; found 442.4165. Anal Calcd $C_{29}H_{51}N_3$ 0.10 H_2O. Theory: C 78.2,1 H 11.59, N 9.44 Found: C 78.21, H 11.55, N 9.49. Alternatively, one could convert the free base of **1** (SR) to the HCl salt for long term storage. For example, the free base of **1(S,R isomer)** (90 mg) was dissolved in absolute

ethanol (11 mL) at 0° C. A 4 M HCl solution (22 mL) was slowly added to the free base solution. The solution was stirred for 30 minutes and then concentrated. The resulting solid was taken up in water and concentrated to remove any remaining ethanol to give the amine hydrochloride salt of **1(SR)** (90 mg, 74%) as a crystalline solid.

R-4-Methyl-2-(3-methyl-butyrylamino)-pentanoic acid methyl ester, (4, R isomer). Diisopropylethylamine, DIEA, (0.95 g, 1.28 mL, 7.32 mmol) was added to a solution of isovaleric acid (0.37 g, 0.4 mL, 3.66 mmol) and D-leucine methyl ester hydrochloride (665 mg, 3.66 mmol) in dichloromethane (20 mL). The addition of DIEA helped to fully dissolve the D-leucine methyl ester hydrochloride. Hexafluorophosphate azabenzotriazole tetramethyl uranium, HATU, (2.78 g, 7.32 mmol), was then added forming a suspension. Upon stirring, the suspension turned light green. The suspension was stirred for 48 h at rt. TLC (2% MeOH in DCM) showed complete consumption of starting material. The reaction was filtered to remove the unconsumed HATU solid and the solid was washed with DCM. The filtrate was poured into a separatory funnel, washed successively with aq. Na_2CO_3, H_2O, 0.01 M HCl, and then water. The organic layers were pooled together, dried over anhydrous Na_2SO_4, filtered, and concentrated to give crude **4** (883 mg). This crude was purified by flash chromatography (0.5% MeOH in DCM) to provide the pure amide **4** (581 mg, 70% yield). **4 (R isomer)**: ^{1}H NMR (400 MHz, $CDCl_3$): δ 5.77(d, 1H, *J*=7.7 Hz), 4.66 (td, 1H, *J*=8.7, 5 Hz), 3.73 (s, 3H), 2.12 (m, 3H), 1.66 (m, 3H), 1.53 (m, 1H), 0.95 (ddd, 12H, *J*=7.5, 6.2, 3 Hz); ^{13}C NMR (100 MHz, $CDCl_3$) δ 173.7, 172.3, 52.1, 50.4, 45.8, 41.6, 26.1, 24.8, 22.7, 22.4, 21.8. High resolution mass spectrum $[M + Na]^+$: theory for $C_{12}H_{23}NNaO_3$: 252.1576; found 252.1580. Anal Calcd $C_{12}H_{23}NO_3$ Theory: C 62.85, H 10.11, N 6.11, Found: C 62.62 H, 10.33, N 6.07.

S-4-Methyl-2-(3-methyl-butyrylamino)-pentanoic acid methyl ester (4, S-isomer): To a solution of L-leucine methyl ester hydrochloride (5 g, 27.5 mmol) and isovaleric acid (3 mL, 2.81 g, 27.5 mmol) in dichloromethane (140 mL) was added diisopropylethylamine, DIEA (9.6 mL, 7.11 g, 55 mmol,) and hexafluorophopsphate azabenzotriazole tetramethyl uronium, (HATU ,21 g, 55 mmol) forming a suspension. Upon stirring, the suspension turned light green and then became milky white overnight. TLC (2% MeOH/DCM) showed consumption of the starting amino ester. The reaction was then filtered to remove the HATU related solids and the solid was washed with DCM. The filtrate was collected and poured into a separatory funnel and washed successively with saturated aq. Na_2CO_3, H_2O, 0.01 M HCl, and H_2O. The organic layer was then collected, dried over anhydrous Na_2SO_4, filtered, and concentrated under reduced pressure to give the crude **4 (S isomer)** (10.4 g). The crude amide **4 (S-isomer)** was consumed in the next step without further purification. **4 (S-isomer)**: ^{1}H NMR: (400 MHz, $CDCl_3$): δ 5.78 (d, 1H, J=7.9 Hz), 4.66 (td, J=8.7 and 5 Hz), 3.73 (s, 3H), 2.12 (m, 3H), 1.66 (m, 3H), 1.53 (m, 1H), 0.95 (m, 12H).

R-4-Methyl-2-(3-methyl-butyrylamino)-pentanoic acid (5, R-isomer): The amide ester, **4 (R-isomer)**, (543 mg, 2.37 mmol), was dissolved in MeOH (22 mL) and 1M NaOH (2.5 mL, 2.5 mmol) was added slowly at 0°C and then the reaction was left to warm to rt and stirred overnight. Complete consumption of the starting amide ester was observed by TLC (2% MeOH in DCM). The reaction mixture was then acidified by adding a slight excess of 1M HCl (2.7 mL, 2.7 mmol) and concentrated under reduced pressure. 0.1 M HCl (30 mL) was added to the resulting solid and the aqueous phase was extracted three times with ethyl acetate. The organic layers were then pooled together, dried over anhydrous Na_2SO_4, filtered and concentrated under reduced pressure to give a white

solid. The crude acid was then taken up in dichloromethane and filtered to yield the pure acid as a white powder (370 mg, 73%). **5 (R-isomer)**: ^{1}H NMR (400 MHz, DMSO-d_6): δ 8.01 (d, 1H, *J*=7.9 Hz), 4.22 (ddd, 1H, *J*=10.2, 7.9, 5.1 Hz), 1.96 (m, 3H), 1.63 (m, 1H), 1.51 (m, 2H), 0.86 (m, 12H); ^{13}C NMR (100 MHz, DMSO-d_6): δ 174.3, 171.6, 50.0, 44.4, 39.8, 25.6, 24.3, 22.9, 21.3, 22.2, 21.1. High resolution mass spectrum $[M + H]^+$: theory for $C_{11}H_{21}NO_3$ 216.1600; found 216.1600. Anal Calcd $C_{11}H_{21}NO_3$ Theory: C 61.37, H 9.83, N 6.51 Found: C 61.36, H 10.00, N 6.43.

S-4-Methyl-2-(3-methyl-butyrylamino)-pentanoic acid (5, S-isomer): The crude **4 (S-isomer)** (6.3 g, 27.5 mmol) was dissolved in MeOH (270 mL) at rt and 1M NaOH was added (28.9 mL, 28.9 mmol) slowly at 0°C and then the reaction was left to warm to rt and stirred overnight. Complete conversion of the starting methyl ester to the salt of **5** was not observed by TLC (2% MeOH in DCM) after overnight stirring. Another 1.05 equiv of NaOH was added and stirred overnight after which, complete conversion of the methyl ester to the salt of **5** was observed. The reaction mixture was acidified by adding 1 M aq. HCl until pH 3 and then concentrated under reduced pressure. The resulting solid was taken up in .01 M HCl (150 mL) and extracted three times with ethyl acetate. The organic layers were combined, dried over anhydrous Na_2SO_4, filtered, and concentrated under reduced pressure to give crude **5 (S-isomer)**. The crude solid was then taken up in cold DCM and filtered to give pure **5 (S-isomer)** as a white solid (3.8 g, 64%). **5 (S-isomer)**: ^{1}H NMR (400 MHz, DMSO-d_6): δ 8.01 (d, 1H, J=7.9 Hz), 4.22 (ddd, 1H, J=10.2, 8.5 Hz), 1.96 (m, 3H), 1.63 (m, 1H), 1.50 (m, 2H), 0.86 (m, 12H); ^{13}C-NMR (100 MHz, DMSO-d_6): 174.3, 171.6, 50.0, 44.4, 39.8, 25.7, 24.3, 22.9, 22.3, 22.2, 21.1. High resolution mass

spectrum $[M + Na]^+$: theory for $C_{11}H_{22}NNaO_3$ 238.1419; found 238.1420. Anal Calcd $C_{11}H_{21}NO_3$ 0.17 H_2O Theory: C 59.67 H 9.71 N 6.33 Found: C 59.67 H 9.38 N 6.30.

3-Cyclohexyl-2-[4-methyl-2-(3-methyl-butyrylamino)-pentanoylamino]-propionic acid methyl ester (6, RR-isomer): To a suspension of 4-methyl-2-(3-methyl-butyrylamino)-pentanoic acid, **5 (R-isomer)**, (356.4 mg, 1.66 mmol) and *R*-2-Amino-3-cyclohexyl-propionic acid methyl ester hydrochloride (380 mg, 1.72 mmol) in DCM (13 mL) was added *N*,*N*-diisopropylethylamine, DIEA, (0.6 mL, 445 mg, 3.44 mmol). Note: The addition of DIEA helped to fully dissolve the carboxylic acid, **5** and the stirred solution turned light green upon addition of DIEA. Hexafluorophosphate Azabenzotriazole Tetramethyl Uronium (HATU, 850 mg, 2.24 mmol) was added and formed a light green suspension. The reaction was stirred overnight at rt, upon which, it became a milky white suspension. TLC (5% MeOH in 95% DCM) was used to monitor the consumption of starting material and the product was visualized using $KMnO_4$ staining. The suspension was filtered to remove the HATU solid, and the filtrate transferred to a separatory funnel and washed successively with saturated aq. Na_2CO_3, H_2O, 0.01 M aq. HCl, and then a final wash with H_2O. The organic layer was collected, dried over anhydrous Na_2SO_4, filtered and concentrated under reduced pressure to provide crude **6** (RR isomer) (851 mg). This crude was purified by flash chromatography (0.5% MeOH in DCM) yielding the pure R,R-diamide ester **6** (403 mg, 64% yield). **6 (RR isomer)**: ^{1}H-NMR (400 MHz, $CDCl_3$): δ 6.41 (d, 1H, *J*=8.5 Hz), 5.86 (d, 1H, *J*=8.2 Hz), 4.59 (m, 1H), 4.49 (td, 1H, *J*=8.5, 5.6), 3.73 (s, 3H), 2.10 (m, 3H), 1.67 (m, 10H), 1.53 (m, 1H), 1.29 (m 1H), 1.16(m, 3H) 0.93(m, 14H); ^{13}C NMR (100 MHz, $CDCl_3$) δ 173.1, 172.5, 172.0, 52.2, 51.3, 50.1, 45.8, 41.0, 30.7, 33.9, 33.3, 32.4, 26.3, 26.1, 26.0, 25.9, 24.7, 22.8, 22.4, 22.3, 22.2. High

resolution mass spectrum [M + Na]$^+$: theory for $C_{21}H_{38}N_2NaO_4$: 405.2729; found 405.2794. Anal Calcd $C_{21}H_{38}N_2O_4$ Theory: C 65.94, H 10.01, N 7.32 Found: C 65.86, H 10.16, N 7.29.

3-Cyclohexyl-2-[4-methyl-2-(3-methyl-butyrylamino)-pentanoylamino]-propionic acid methyl ester (6, SS isomer): To a solution of 4-methyl-2-(3-methyl-butyrylamino)-pentanoic acid, **5, (S-isomer**, 2.22 g, 10.3 mmol) and 2*S*-Amino-3-cyclohexyl-propionic acid methyl ester hydrochloride (2.32 g, 10.5 mmol) in DCM (80 mL) was added diisopropylethylamine (DIEA, 3.5 mL, 2.7 g, 20.9 mmol). Note: DIEA addition helped to dissolve **5** and the stirred solution turned light green. Hexafluorophosphate azabenzotriazole tetramethyl uronium (HATU, 8.1 g, 21.3 mmol) was added forming a light green suspension. The reaction was stirred overnight at rt upon which it became a milky white suspension. TLC (5% MeOH/DCM) was used to monitor the consumption of the starting acid **5**. The suspension was filtered to remove the HATU solid and the filtrate was transferred to a separatory funnel and washed successively with saturated aq. Na_2CO_3, H_2O, 0.01 M HCl, and then a final wash with H_2O. The organic layer was collected, dried over anhydrous Na_2SO_4, filtered and concentrated under reduced pressure. The crude **6** (3.95 g), was purified by flash chromatography (0.5% MeOH in DCM R_f=0.2) yielding the pure S,S-methyl ester (3.11 g, 79% yield). **6 (SS isomer)**: ^{1}H-NMR (400 MHz, $CDCl_3$): δ 6.41 (d, 1H, J=7.9 Hz), 6.22 (d, 1H, J=8.4 Hz), 4.55 (m, 2H), 3.72 (s, 3H), 2.10 (m, 3H), 1.67 (m, 8H), 1.54 (m, 2H), 1.23 (m, 4H), 0.88 (m, 14H); ^{13}C-NMR (100 MHz, $CDCl_3$): δ 173.2, 172.6, 172.0, 52.2, 51.3, 50.2, 45.9, 41.1 39.8, 34.0, 33.4, 32.4, 26.3, 26.2, 26.1, 26.0, 24.7, 22.9, 22.5, 22.4, 22.2. High resolution mass

spectrum [M + Na]+: theory for $C_{21}H_{38}N_2NaO_4$ 405.2729; found 405.2728. Anal Calcd $C_{21}H_{38}N_2O_4$ Theory: C 65.94, H 10.01, N 7.32 Found: C 66.17, H 9.97, N 7.27.

3-Cyclohexyl-2-[4-methyl-2-(3-methyl-butyrylamino)-pentanoylamino]-propionic acid methyl ester (6, SR isomer): To a solution of 4-Methyl-2-(3-methyl-butyrylamino)-pentanoic acid, (**5, R isomer**), (1 g, 4.64 mmol) and L-cyclohexylalanine methyl ester hydrochloride (S-isomer, 1.03 g, 4.64 mmol) in DCM (40 mL) was slowly added diisopropylethylamine, DIEA, (1.20 g, 1.62 mL, 9.28 mmol) at 0 °C. DIEA helped to dissolve **5** and the solution turned green upon its addition. HATU (3.53 g, 9.28 mmol) was then added forming a light green suspension. The reaction was then warmed to rt and stirred overnight forming a white suspension. TLC (5% MeOH/DCM) was used to confirm consumption of the starting acid. The crude reaction mixture was then filtered and washed with DCM (3x). The organic layers were pooled and transferred to a separatory funnel and washed with saturated aq. Na_2CO_3, H_2O, 0.01 M HCl, and a final H_2O wash. The organic layers were pooled together, dried over anhydrous Na_2SO_4, filtered, and concentrated under reduced pressure to give a crude solid (1.92 g). The crude was purified by column chromatography (1% MeOH in DCM) yielding the pure diamide ester**,** **6 (SR isomer)** as a white solid (1.27 g, 72%). **6 (SR isomer)**: ^{1}H-NMR (400 MHz, $CDCl_3$): δ 6.93 (d, 1H, J=8.4 Hz), 6.22 (d, 1H, J=8.4 Hz), 4.57 (m, 2H), 3.69 (s, 3H), 2.08 (m, 3H), 1.62 (m, 10H), 1.22 (m, 4H), 0.94 (m, 14H); ^{13}C-NMR (100 MHz): δ 173.1, 172.7, 172.0, 52.2, 51.3, 50.1, 45.8, 40.9, 39.6, 38.6, 34.1, 33.5, 32.3, 26.3, 26.14, 26.11, 26.0, 24.8, 22.8, 22.4, 22.3, 22.2. High resolution mass spectrum [M + H]+: theory for $C_{21}H_{39}N_2O_4$ 383.2904; found 383.2913.

4-Methyl-2-(3-methyl-butyrylamino)-pentanoic acid (1-carbamoyl-2-cyclohexyl-ethyl)-amide (7, RR isomer): 3-Cyclohexyl-2-[4-methyl-2-(3-methyl-butyrylamino)-pentanoylamino]-propionic acid methyl ester (**6-**RR isomer, 363 mg, 0.95 mmol,) was dissolved in MeOH (10 mL). To this solution, a vigorous stream of ammonia gas was added at 0°C for 1 h. The ammonia gas was produced by heating concentrated ammonium hydroxide between 35°-40°C and bubbling the NH_3 gas through a glass trap (to prevent back flow) and then into the solution. After 1 hour, the ammonia gas production ceased and the solution was stoppered and warmed to rt and stirred after brief venting. TLC (5% MeOH in DCM, R_f=0.6) was used to monitor conversion of **6** into **7**. After 5 days, the solution was concentrated under reduced pressure forming a white solid. The white solid was taken up in cold DCM (50 mL) and filtered. The filtered precipitate was the desired R,R triamide **7** (311 mg, 89%). **7 (RR isomer)**: ^{1}H NMR (400 MHz, DMSO-d$_6$): δ 7.94 (d, 1H, J= 6.8 Hz), 7.72 (d, 1H, J=7), 7.20 (s, 1H), 6.95 (s, 1H), 4.26 (m, 2H), 1.99 (s, 3H), (1.61 m, 6H), 1.43 (s, 4H), 1.25(s, 1H), 1.11 (m, 3H), 0.86 (s, 14H); ^{13}C-NMR (100 MHz, DMSO-d$_6$): δ 173.8, 171.6, 171.4, 51.0, 50.0, 44.3, 40.2, 39.3, 33.3, 33.1, 31.7, 25.8, 25.6, 25.3, 24.0, 22.7, 22.1, 22.0, 21.4. High resolution mass spectrum $[M + Na]^+$: theory for $C_{20}H_{37}N_3NaO_3$: 390.2733; found 390.2746. Anal Calcd $C_{20}H_{37}N_3O_3$ Theory: C 65.36, H 10.15, N 11.43; Found: C 65.31, H 10.16, N 11.32.

4-Methyl-2-(3-methyl-butyrylamino)-pentanoic acid (1-carbamoyl-2-cyclohexyl-ethyl)-amide (7, SS-isomer): 3-Cyclohexyl-2-[4-methyl-2-(3-methyl-butyrylamino)-pentanoylamino]-propionic acid methyl ester, **6 (SS isomer),** (3.09 g, 8.1 mmol) was dissolved in MeOH (90 mL). To this solution a vigorous stream of ammonia gas (NH_3) was introduced at 0° C, while stirring for 1 hour. The ammonia gas was produced by

heating ammonium hydroxide between 35-40° C, then bubbling the NH_3 gas (through a glass trap to prevent backflow) into the solution. After 1 hour the introduction of ammonia gas was ceased. The solution was stoppered and warmed to rt, vented briefly and stirred for 3 days. After 3 days, TLC (5% MeOH/DCM) showed the presence of the starting diamido ester **6** was still present. Another vigorous stream of NH_3 gas was introduced at 0° C while stirring for 1 hour after which, a precipitate was observed to form in the solution. After stirring overnight, a large amount of precipitate had formed. The mixture was concentrated under reduced pressure, taken up in cold DCM, filtered and a solid collected (precipitate A; 1.13 g). The filtrate was observed to contain a mixture of the ester **6** and crude amide **7** by TLC (5% MeOH in DCM). The filtrate was then taken up in MeOH (50 mL) and NH_3 gas was introduced for 1 hour at 0° C while stirring. After an hour, introduction of NH_3 gas was stopped and the solution was allowed to stir for 3 days. After 3 days the reaction mixture was concentrated under reduced pressure, taken up in cold DCM, filtered and precipitate B was collected. After NMR confirmed their identity, the two precipitates (A and B) were combined to provide the pure triamide **7** (SS isomer, 2.75 g, 93% yield). **7 (S,S isomer)**: ^{1}H-NMR (400 MHz, DMSO-d_6): δ 7.94 (d, 1H, J=8 Hz), 7.72 (d, 1H, J=8.4 Hz), 7.20 (s, 1H), 6.95 (S, 1H), 4.25 (m, 2H), 1.99 (m, 3H), 1.60 (m, 6H), 1.43 (m, 4H), 1.13 (m, 4H), 0.85 (m, 14H) ^{13}C-NMR(125 MHz, DMSO-d_6): δ 173.8,171.5,171.4, 51.0, 50.0, 44.3, 40.2, 39.3, 33.3, 33.1, 31.7, 25.8, 25.6, 25.3, 24.0, 22.7, 22.1, 22.0, 21.4. High resolution mass spectrum $[M + Na]^+$: theory for $C_{20}H_{37}N_3NaO_3$ 390.2733; found 390.2732. Anal Calcd $C_{20}H_{37}N_3O_3$ Theory: C 65.36, H 10.15, N 11.43, Found: C 65.52, H 10.18, N 11.57.

4-Methyl-2-(3-methyl-butyrylamino)-pentanoic acid (1-carbamoyl-2-cyclohexyl-ethyl)-amide (7 SR-isomer): 3-Cyclohexyl-2-[4-methyl-2-(3-methyl-butyrylamino)-pentanoylamino]-propionic acid methyl ester, **6** (SR isomer**,** 1.18 g, 3.08 mmol) was dissolved in MeOH (30 mL). To this solution, a vigorous stream of ammonia gas was introduced (through a glass trap to prevent backflow) into the solution at 0°C, while stirring for 1 h. The ammonia gas was produced by gently heating concentrated ammonium hydroxide between 35-40 °C and bubbling the gas into the solution containing the methyl ester. After 1 hour, the ammonia gas production was ceased and the solution stoppered and warmed to rt, vented briefly and stirred. TLC (5% MeOH in DCM) was used to monitor conversion of the starting diamide ester into the triamide. After 7 days, the solution was concentrated under reduced pressure to form a white solid. The white sold was taken up in cold DCM then filtered. The precipitate was collected as the pure triamide, **7 (SR isomer)** (1.02 g, 90%). **7 (SR isomer)**: ^{1}H-NMR (400 MHz, DMSO-d$_6$): δ 8.23 (d, 1H, J=8.5 Hz), 8.01 (d, 1H, J=6.8), 7.26 (s, 1H), 7.00 (s, 1H), 4.17 (m, 2H), 1.97 (m, 3H), 1.51 (m, 10H), 1.24 (br, 1H), 1.09 (m, 4H), 0.82 (m, 14H)); ^{13}C-NMR (125 MHz): 174.3,172.1, 51.9,49.9, 44.2, 40.4, 38.5, 33.6, 33.5, 31.2, 26.04, 25.98, 25.7, 25.6, 24.2, 22.4, 22.31, 22.26 22.2, High resolution mass spectrum [M + H]$^+$: theory for $C_{20}H_{38}N_3O_3$ 368.2908; found 368.2910; Anal Calcd $C_{20}H_{37}N_3O_3$ Theory: C 65.36, H 10.15, N 11.43, Found: C 65.44, H 10.14, N 11.43.

N^1-(2-Amino-1-cyclohexylmethyl-ethyl)-4-methyl-N2-(3-methyl-butyl)-pentane-1,2-diamine (8, RR isomer): Borane-tetrahydrofuran complex (1M solution, 5.8 mmol, 5.8 mL, 8 equiv) was added via syringe to 4-methyl-2-(3-methyl-butyrylamino)-pentanoic acid (1-carbamoyl-2-cyclohexyl-ethyl)-amide (**7 RR isomer**), (268 mg, 0.73 mmol) at rt. The

mixture was then heated to reflux for 2 days. The reaction mixture was concentrated under reduced pressure to give a residue. A 10% concentrated HCl/Methanol solution (12 mL) was then added at 0° C and stirred for 3 days. The mixture was concentrated under reduced pressure to give a residue. 1M NaOH was added to the residue at 0°C until the aqueous phase reached pH 12. The material was then extracted three times with DCM, dried over anhydrous sodium sulfate, filtered and concentrated to yield R,R-triamine **8** as a clear oil (230 mg, 97%) without further purification. **8 (RR isomer)**: ^{1}H-NMR (400 MHz, $CDCl_3$): δ 2.62 (m, 7H), 2.37 (dd, 1H, J=10.9, 8 Hz), 1.64 (m, 11H), 1.27 (m, 10H), 0.90 (m, 14H); ^{13}C-NMR (100 MHz): δ 56.0, 54.0, 49.3, 44.2, 43.9, 41.4, 39.6, 38.6, 33.6, 32.9, 32.6, 25.6, 25.4, 25.3, 25.1, 24.1, 22.3, 21.8, 21.7, 21.6.

N^1-(2-Amino-1-cyclohexylmethyl-ethyl)-4-methyl-N2-(3-methyl-butyl)-pentane-1,2-diamine (8, SS isomer): Borane-tetrahydrofuran complex (1M solution, 58.14 mL, 58.14 mmol, 8 equiv) was added via syringe to 4-methyl-2-(3-methyl-butyrylamino)-pentanoic acid (1-carbamoyl-2-cyclohexyl-ethyl)-amide, (**7,** SS isomer), (2.67 g, 7.27 mmol). The mixture was then heated to reflux and stirred for 3 days after which, the solution was concentrated under reduced pressure to give a residue. A 10% concentrated HCl/MeOH solution (65 mL) was added at 0°C and stirred for 3 days. 1M NaOH was added at 0°C until the aqueous phase reached pH 12. The material was then extracted 3 times with DCM, dried over anhydrous sodium sulfate, filtered and concentrated to give the crude triamine **8** as a crude yellow oil (2.5 g: 105%), which was used without further purification. **8 (SS isomer)**: ^{1}H-NMR (400 MHz $CDCl_3$): δ 2.61 (m, 6H), 2.37 (dd, 1H, J=11.6, 5.9 Hz), 1.82 (S, 3H), 1.58 (m, 8H), 1.30 (m, 11H), 0.90 (m, 14H); ^{13}C-NMR (100 MHz, $CDCl_3$): δ

62.2, 56.0, 55.8, 50.2, 45.1, 44.8, 42.3, 40.5, 39.5, 34.9, 34.5, 33.8, 33.6, 26.5, 26.30, 26.26, 26.1, 25.1, 23.2, 22.7, 22.6, 18.9, 13.8, 0.94.

N^1-(2-Amino-1-cyclohexylmethyl-ethyl)-4-methyl-N2-(3-methyl-butyl)-pentane-1,2-diamine (8, SR isomer): Borane-tetrahydrofuran (1 M solution, 22 mL, 22 mmol) was added via syringe to 4-methyl-2-(3-methyl-butyrylamino)-pentanoic acid (1-carbamoyl-2-cyclohexyl-ethyl)-amide, **7 (SR isomer)**, (978 mg, 2.66 mmol) at rt. The mixture was then heated to reflux for 2 days. The reaction mixture was concentrated under reduced pressure to give a residue. A 10% concentrated HCl/MeOH solution (20 mL) was then added at 0°C and stirred for 3 days. The mixture was concentrated under reduced pressure to give a residue. 1 M NaOH was added at 0°C until the aqueous phase reached pH 12. The material was then extracted three times with DCM, dried over anhydrous sodium sulfate, filtered and concentrated to give the crude triamine **8 (SR)** as a clear oil (832 mg, 96%). This was used without further purification. **8 (SR isomer)**: ^{1}H-NMR (400 MHz, $CDCl_3$): δ 2.57 (m, 8H), 2.38 (dd, 1H, J=11.5, 6.9 Hz), 1.66 (m, 9H), 1.27 (m, 13H), 0.91 (m, 14H); ^{13}C-NMR (100 MHz, $CDCl_3$): δ 56.9, 56.0, 50.2, 45.3, 45.0, 42.6, 40.6, 39.6, 34.5, 33.8, 33.6, 26.5, 26.3, 26.1, 25.1, 22.1, 22.8, 22.64, 22.61, 0.92.

N-(3-Cyclohexyl-2-[4-methyl-2-(3-methyl-butylamino)-pentylamino]-propyl)-benzamide (9, RR isomer): A solution of *N*-(benzoyloxy)succinimide (131 mg, 0.6 mmol, 1 equiv) in DCM (1 mL) was added dropwise to a stirred solution of **8** (RR isomer, 196 mg, 0.6 mmol, 1 equiv) in DCM (1 mL) at 0° C. The reaction mixture was allowed to warm up to room temperature and stirred overnight. After TLC (7% MeOH, 1% NH_4OH, in DCM) showed complete consumption of the starting triamine, the reaction mixture was washed with aqueous Na_2CO_3, dried over anhydrous Na_2SO_4, filtered, and concentrated to give

crude **9** (RR isomer). The crude (241 mg) was purified by flash chromatography (2% MeOH, 1% NH_4OH in DCM) to yield pure benzamide **9 (RR isomer)** as an oil (138 mg, 53%). **9 (RR isomer)**: ^{1}H-NMR (400 MHz, $CDCl_3$): δ 7.84 (m, 2H), 7.46 (m, 3H), 7.33 (s, 1H), 3.58 (dt, 1H, J=13.6, 4.6 Hz), 3.26 (ddd, 1H, J=13.5, 6.7, 5.3 Hz), 2.88 (m, 1H), 2.57 (m, 6H), 2.55 (m, 2H), 1.60 (m, 8H, includes H_2O), 1.30 (m, 12H) 0.90 (m, 9H), 0.83 (m, 6H); ^{13}C-NMR (100 MHz, $CDCl_3$): δ 167.5, 134.8, 131.2, 128.4, 127.0, 55.6, 54.1, 48.8, 44.6, 42.6, 41.9, 40.7, 39.1, 34.3, 33.6, 33.5, 26.5, 26.22, 26.20, 26.0, 26.0, 23.1, 22.6, 22.5.

N-(3-Cyclohexyl-2-[4-methyl-2-(3-methyl-butylamino)-pentylamino]-propyl)-benzamide (9, SS-isomer): A solution of *N*-(benzoyloxy)succinimide (940 mg, 4.29 mmol) in DCM (4 mL) was added dropwise to a stirred solution of the triamine **8 SS-isomer** (1.40 g, 4.29 mmol) in DCM (40 mL) at 0° C. The reaction mixture was allowed to warm to room temperature and stirred overnight. After TLC (7% MeOH, 1% NH_4OH, in DCM) showed complete consumption of the starting triamine, the reaction mixture was washed with aq. Na_2CO_3, the organic layer was separated, dried over anhydrous Na_2SO_4, filtered, and concentrated to give crude benzamide **9**. The crude (1.77 g) was purified by flash chromatography (2% MeOH, 1% NH_4OH in DCM) to remove the remaining *N*-(benzoyloxy) succinimide and give **9 (SS-isomer)** as an oil (1.18 g, 64%). **9 (SS isomer)**: ^{1}H-NMR (400 MHz, $CDCl_3$): δ 7.85 (m, 2H), 7.44 (m, 4H), 3.60 (dt, 1H, J=13.8, 4.5 Hz)), 3.25 (m, 1H), 2.89 (m, 1H), 2.61 (m, 7H), 1.41 (m, 20H), 0.89 (m, 8H), 0.83 (m, 6H); ^{13}C-NMR (100 MHz, $CDCl_3$): δ 167.5, 134.6, 131.2, 128.4, 127.1, 55.6, 54.1, 48.5, 44.5, 42.6, 41.6, 40.7, 38.8, 34.3, 33.59, 33.55, 26.5, 26.21, 26.18, 26.0, 25.0, 23.1, 22.53, 22.50.

N-(3-Cyclohexyl-2-[4-methyl-2-(3-methyl-butylamino)-pentylamino]-propyl)-benzamide (9, SR-isomer): To a solution of crude N[1]-(2-Amino-1-cyclohexylmethyl-ethyl)-4-methyl-N2-(3-methyl-butyl)-pentane-1,2-diamine (**8 SR-isomer,** 827 mg, 2.54 mmol) in DCM (4 mL) was slowly added a solution of *N*-(benzoyloxy)succinimide (557 mg, 2.54 mmol) in DCM (4 mL) at 0°C. The reaction was warmed to rt and stirred overnight. TLC (7% MeOH 1% NH_4OH, in DCM) was used to confirm complete conversion of **8** to the benzamide **9**. The reaction mixture was washed with aq. saturated Na_2CO_3, the organic layer was separated, dried over anhydrous sodium sulfate, and filtered to give the crude benzamide (3.07 g). The crude was purified by flash chromatography (2% MeOH, 1% NH_4OH, in DCM) to yield the pure benzamide **9 (SR)** as an orange oil (0.718 g, 66%). **9 (SR isomer)**: ^{1}H-NMR (400 MHz, $CDCl_3$): δ 7.86 (m, 2H), 7.42 (m, 4H), 3.59 (dt, 1H, J=13.7, 4.4 Hz), 3.27 (dt, 1H, J=13.5, 6 Hz), 2.67 (m, 6H), 2.46 (dd, 1H, J=11.9, 6.4 Hz), 1.61 (m, 7H), 1.26 (m, 10H), 0.86 (m, 14H); ^{13}C-NMR (100 MHz, $CDCl_3$): δ 167.4, 134.6, 131.1, 128.3, 127.0, 55.7, 54.2, 48.5, 44.8, 42.4, 41.6, 40.8, 38.7, 34.3, 33.6, 33.5, 26.4, 26.2, 26.1, 26.0, 25.0, 23.1, 22.49, 22.45.

N-(3-Cyclohexyl-2-[5-isobutyl-4-(3-methyl-butyl)-2,3-dioxo-piperazin-1-yl]-propyl)-benzamide (10 RR isomer): To a solution of benzamide **9 (RR isomer)** (129 mg, 0.3 mmol 1 equiv) in DCM (7 mL) at 0°C was slowly added a 5-fold excess of oxalyldiimidazole (285 mg, 1.51 mmol, 5 equiv) in DCM (8 mL). The resulting reaction mixture was allowed to stir at room temperature for 3 days until complete consumption of starting material was observed by TLC (7% MeOH, 1% NH_4OH in DCM). The mixture was then concentrated under reduced pressure to provide a crude residue. The residue (400 mg) was purified by flash chromatography (2% MeOH in DCM) to give the pure cyclized

product **10 (RR isomer)** as a white powder (94 mg, 65% yield), **10 (RR isomer)**: ^{1}H-NMR (400 MHz, $CDCl_3$): δ 7.76 (d, 2H, J=7), 7.45 (m, 4H), 6.99 (s, 1H), 4.88 (s, 1H), 3.95 (m, 1H), 3.62 (m, 2H), 3.42 (m, 2H), 3.25 (d, 1H, J=13), 2.86 (m, 1H), 1.53 (m, 16H), 1.22 (m, 7H), 0.94 (m, 8H), 0.79 (dd, 6H); ^{13}C-NMR (100 MHz, $CDCl_3$): δ 167.4, 159.6, 157.1, 133.3, 131.6, 128.7, 127.1, 52.0, 44.6, 43.2, 40.6, 37.0, 36.8, 34.6, 33.6, 32.9, 26.3, 26.12, 26.08, 25.9, 25.1, 22.9, 22.6, 22.3, 21.5. High resolution mass spectrum [M + Na]$^+$: theory for $C_{29}H_{45}N_3NaO_3$ 506.3359; found 506.3363. Anal Calcd $C_{29}H_{45}N_3O_3$ Theory: C 72.01, H 9.38, N 8.69, Found: C 71.71 H 9.35 N 8.58.

N-(3-Cyclohexyl-2-[5-isobutyl-4-(3-methyl-butyl)-2,3-dioxo-piperazin-1-yl]-propyl)-benzamide (**10, SS isomer):** To a solution of the benzamide **9** (SS-isomer, 1.17 g, 2.73 mmol) in DCM (64 mL) at 0° C was slowly added a five-fold excess of oxalyldiimidazole (2.59 g, 13.63 mL) in DCM (71 mL). The resulting suspension was allowed to stir at room temperature for 3 days until complete consumption of the starting benzamide was observed by TLC (7% MeOH, 1% NH_4OH in DCM). The mixture was then concentrated under reduced pressure. The crude reaction residue (2.78 g) was purified by flash chromatography (2% MeOH in DCM) to give the pure S,S-diketopiperazine **10** as a white powder (1.00 g, 76% yield). **10 (SS isomer)**: ^{1}H-NMR (400 MHz, $CDCl_3$): δ 7.78 (m, 2H), 7.48 (m, 1H), 7.40 (m, 2H), 7.14 (s, 1H), 4.88 (s, 1H), 3.92 (ddd, 1H, J=13.5, 9.8, 5.8 Hz), 3.65 (m, 2H), 3.44 (m, 2H), 3.26 (dd, 1H, J=13.2, 1.5 Hz), 2.86 (ddd, 1H, J=13.5, 9.5, 5.4), 1.81 (m, 2H), 1.66 (m, 5H), 1.5 (m, 8H), 1.25 (m, 6H), 0.93 (m, 8H), 6.79 (m, 6H); ^{13}C-NMR (100 MHz, $CDCl_3$): δ 167.4, 159.4, 157.0, 133.3, 131.6, 128.6, 127.0, 51.9, 44.6, 43.0, 40.5, 37.0, 36.7, 34.5, 33.6, 32.9, 26.2, 26.1, 26.0, 25.9, 25.0, 22.8, 22.5, 22.3, 21.4. High resolution mass spectrum [M+Na]$^+$: theory for $C_{29}H_{45}N_3NaO_3$: 506.3359; found

506.3345. Anal Calcd $C_{29}H_{45}N_3O_3$ Theory: C 72.01, H 9.38, N 8.69, Found: C 72.11, H 9.48, N 8.71.

N-(3-Cyclohexyl-2-[5-isobutyl-4-(3-methyl-butyl)-2,3-dioxo-piperazin-1-yl]-propyl)-benzamide (10, SR-isomer): To a solution of the diamine benzamide **9 (SR isomer)** (710 mg, 1.65 mmol) in DCM (64 mL) at 0°C was slowly added a five-fold excess of oxalyldiimidazole (1.59 g, 8.35 mmol) in DCM (71 mL). The resulting suspension was allowed to stir at room temperature for 3 days until complete consumption of the starting benzamide was observed by TLC (7% MeOH, 1% NH_4OH in DCM). The mixture was then concentrated under reduced pressure. The crude reaction residue (2.18 g) was purified by flash chromatography (2% MeOH in DCM) to give the pure diketopiperazine as a white powder (602 mg, 75% yield). **10 (SR isomer)**: ^{1}H-NMR (400 MHz, $CDCl_3$): δ 7.73 (m, 2H), 7.45 (m, 3H), 6.80 (s, 1H), 4.85 (m, 1H) 3.92 (m, 2H), 3.66 (dd, 1H J=12.8, 4.1 Hz), 3.41 (m, 1H), 3.25 (m, 2H), 2.81 (m, 1H), 1.94 (m, 1H), 1.69 (m, 7H), 1.37 (m, 12H), 0.9 (m,14H); ^{13}C-NMR (100 MHz, $CDCl_3$): 168.0, 158.5, 156.4, 134.0, 131.4, 128.4, 127.2, 51.8, 44.2, 42.5, 41.5, 40.1, 37.1, 36.5, 34.3, 33.9, 32.7, 26.4, 26.2, 26.0, 25.8, 25.1, 23.2, 22.6, 22.1, 21.2 High resolution mass spectrum $[M + H]^+$: theory for $C_{29}H_{45}N_3O_3$ 484.3534; found 484.3552. Anal Calcd $C_{29}H_{45}N_3O_3$ 0.12 H_2O. Theory: C 71.37, H 9.34, N 8.61, Found: C 71.37, H 9.42, N 8.63.

S4. <u>Circular Dichroism Sample Processing</u>. Circular dichroism (CD) absorption spectra of the diastereomers of compound **1** were measured using a Jasco J-810 CD spectrometer. Samples were prepared in buffer solution (1X PBS, pH 7.4) and run at a 200 nm/min scan rate in the wavelength range from 210 to 225 nm, with 1 mM compound. All experiments were done using a quartz cell of 4 mm path length at rt.

Circular Dichroism Figures

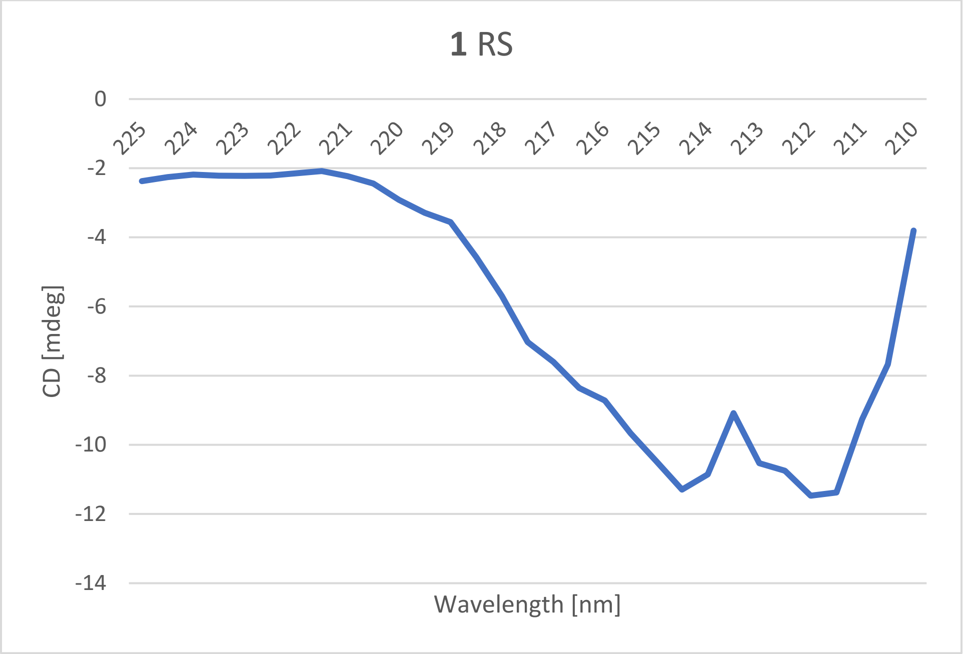

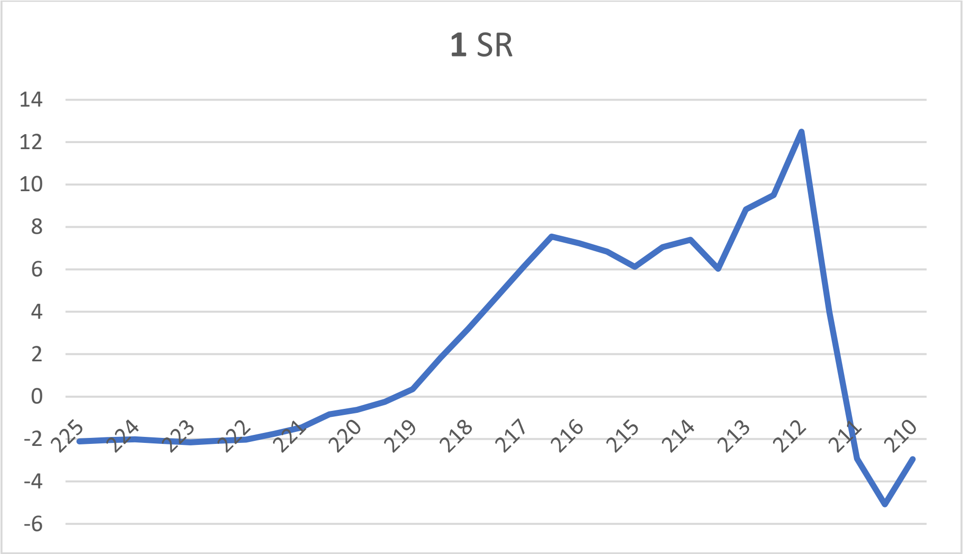

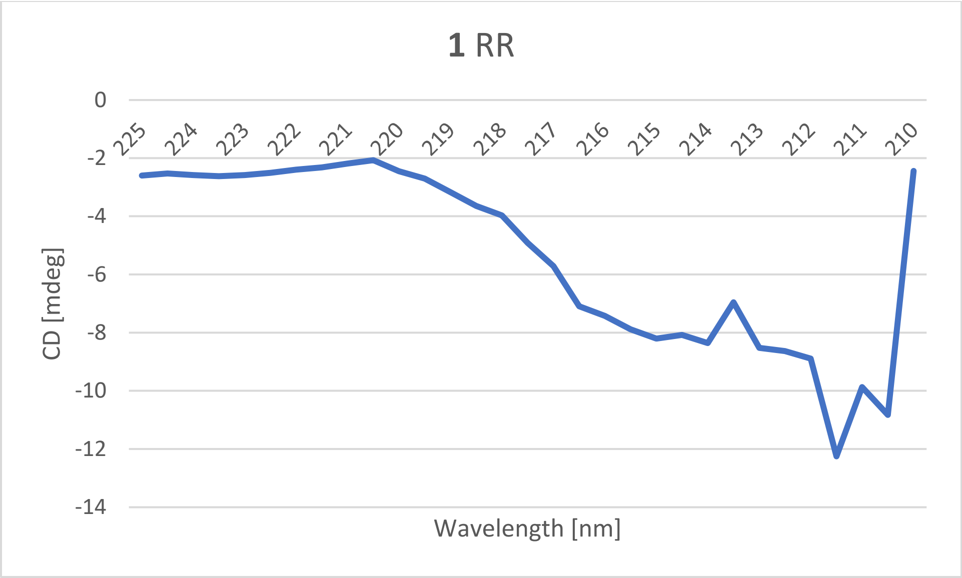

1 RR
0
-2
-4
-6
-8
-10
-12
-14
225
224
223
222
221
220
219
218
217
216
215
214
213
212
211
210
CD [mdeg]
Wavelength [nm]

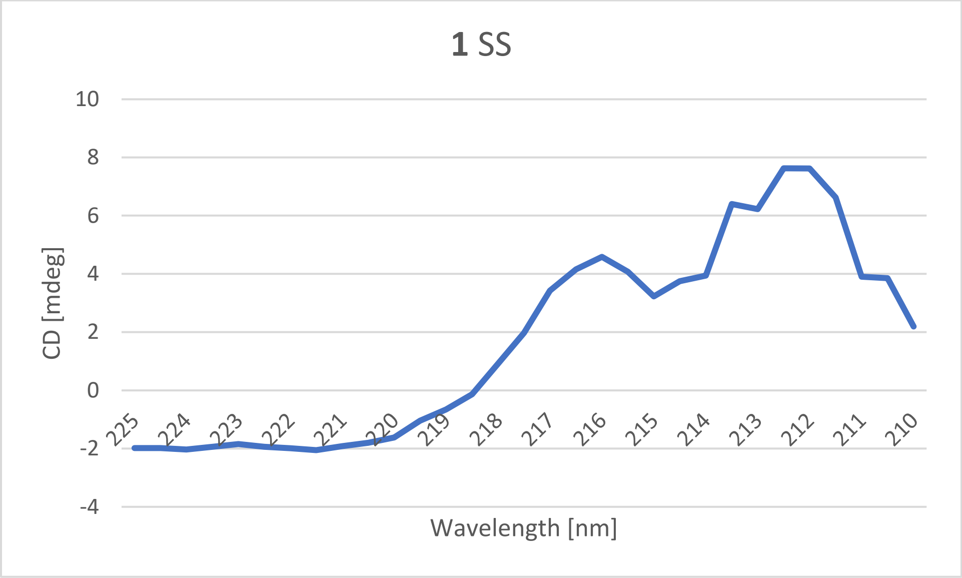

1 SS
10
8
6
4
2
0
-2
-4
225
224
223
222
221
220
219
218
217
216
215
214
213
212
211
210
CD [mdeg]
Wavelength [nm]

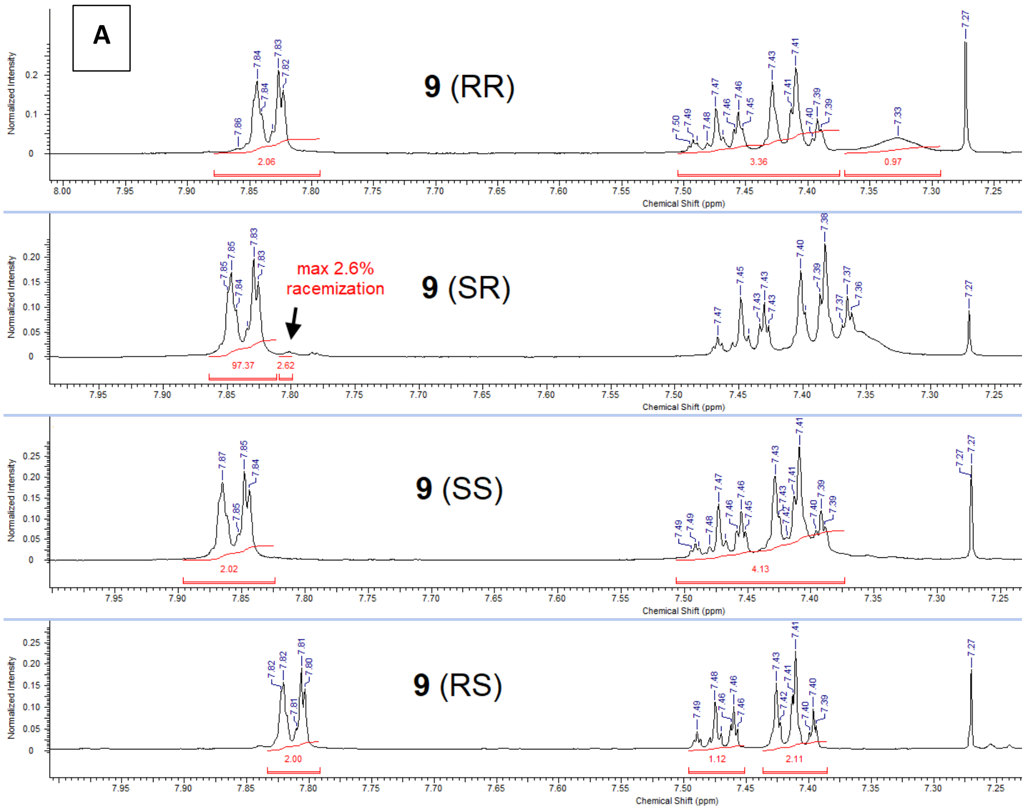
A
9 (RR)
9 (SR)
max 2.6% racemization
9 (SS)
9 (RS)
Normalized Intensity
Chemical Shift (ppm)

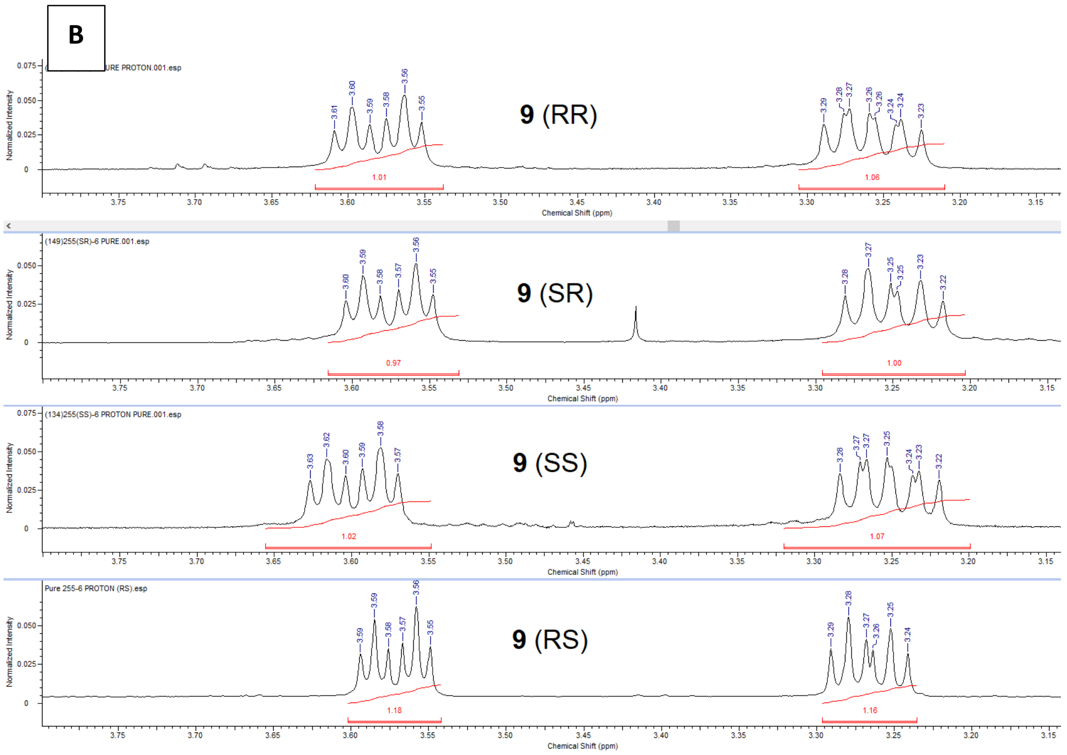
B
9 (RR)
9 (SR)
9 (SS)
9 (RS)
Normalized Intensity
Chemical Shift (ppm)

C

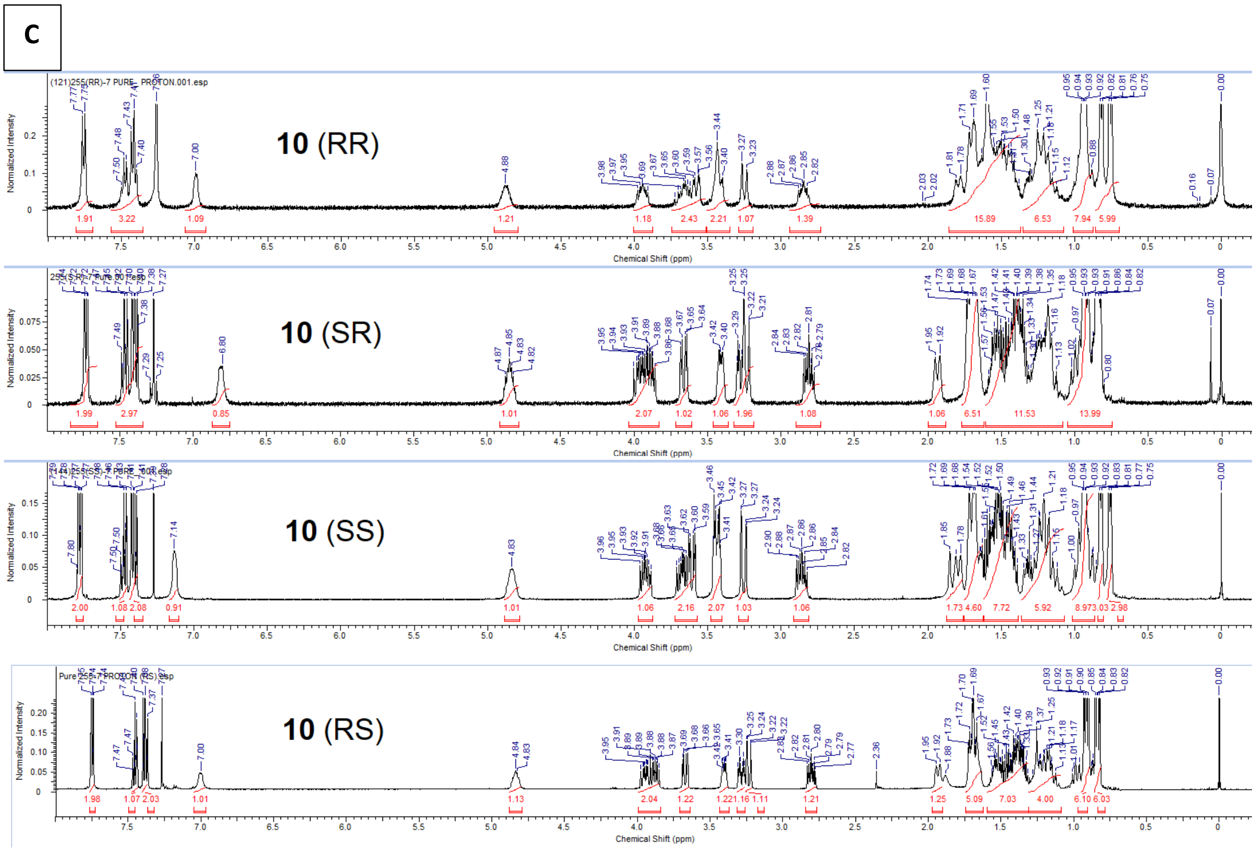
10 (RR)
10 (SR)
10 (SS)
10 (RS)
Normalized Intensity
Chemical Shift (ppm)

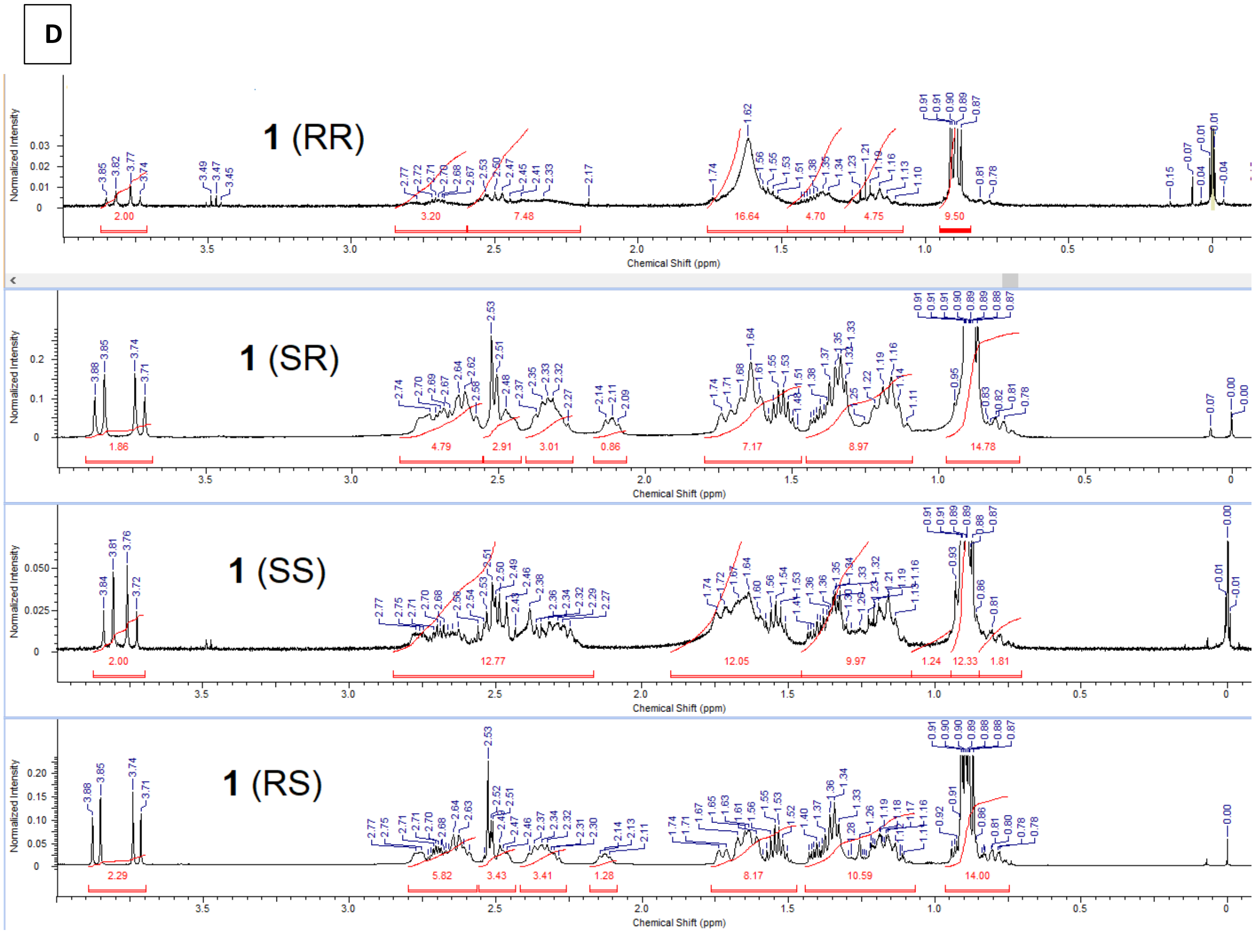

Figure S3. Assessment of racemization in compounds 9, 10, and 1. Since compound **9** is generated after the two HATU mediated coupling steps, it provided a convenient ortho CH (aryl signal) near 7.80 ppm (**panel A**) and another at 3.62 ppm (**panel B**) that were resolved in the different diastereomers of **9**. Thus, one estimate possible racemization by integrating the ^{1}H NMR spectrum. As shown above, only the SR isomer appeared to have any racemization and it was estimated to be less than 2.6%. This estimate is likely too high as well because it quantifies the amount of doubly racemized RS isomer that could be present in the SR isomer, which is very unlikely to be generated. In sum, we concluded that racemization was barely detectable through compound **9.** As shown in **panel C**, little to no racemization was also observed in the penultimate compound **10**, whose diastereomers had resolved ^{1}H NMR signals from 1.75-2 ppm and near 3.25 ppm. As shown in **panel D**, the benzylic CH_2 in the diastereomers of **1** appear as a doublet of doublets centered near 3.8 (far left side of panel D). Due to the magnitude of the coupling constants, one could resolve and identify the presence of the RR (3.77 ppm) and SS (3.76 ppm) isomers in the SR isomer.

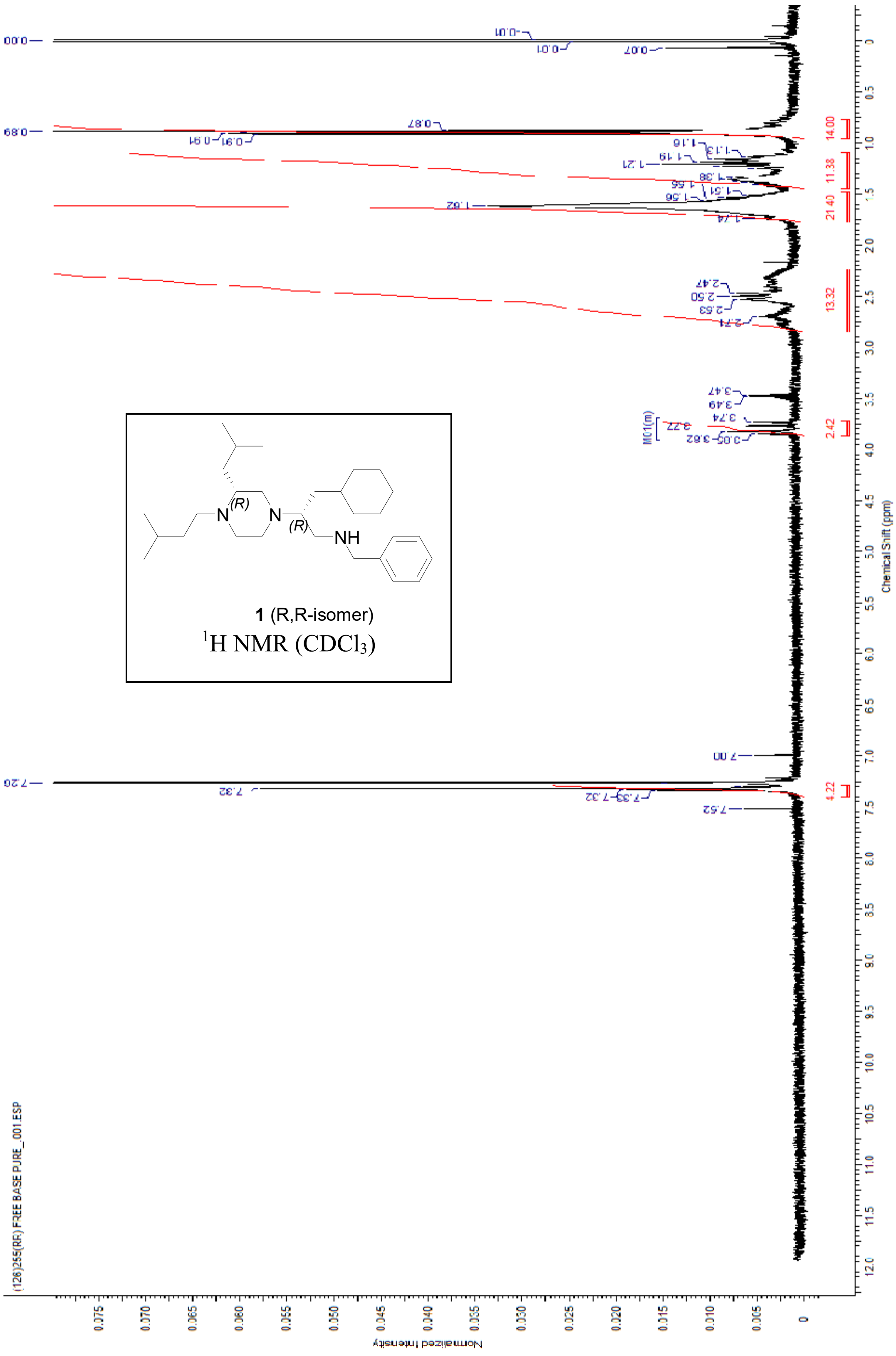

1 (R,R-isomer)

^{1}H NMR ($CDCl_3$)

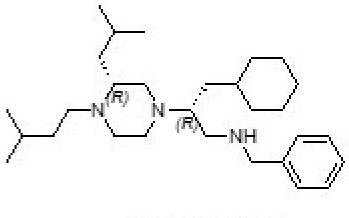

1 (R,R-isomer)

^{13}C NMR ($CDCl_3$)

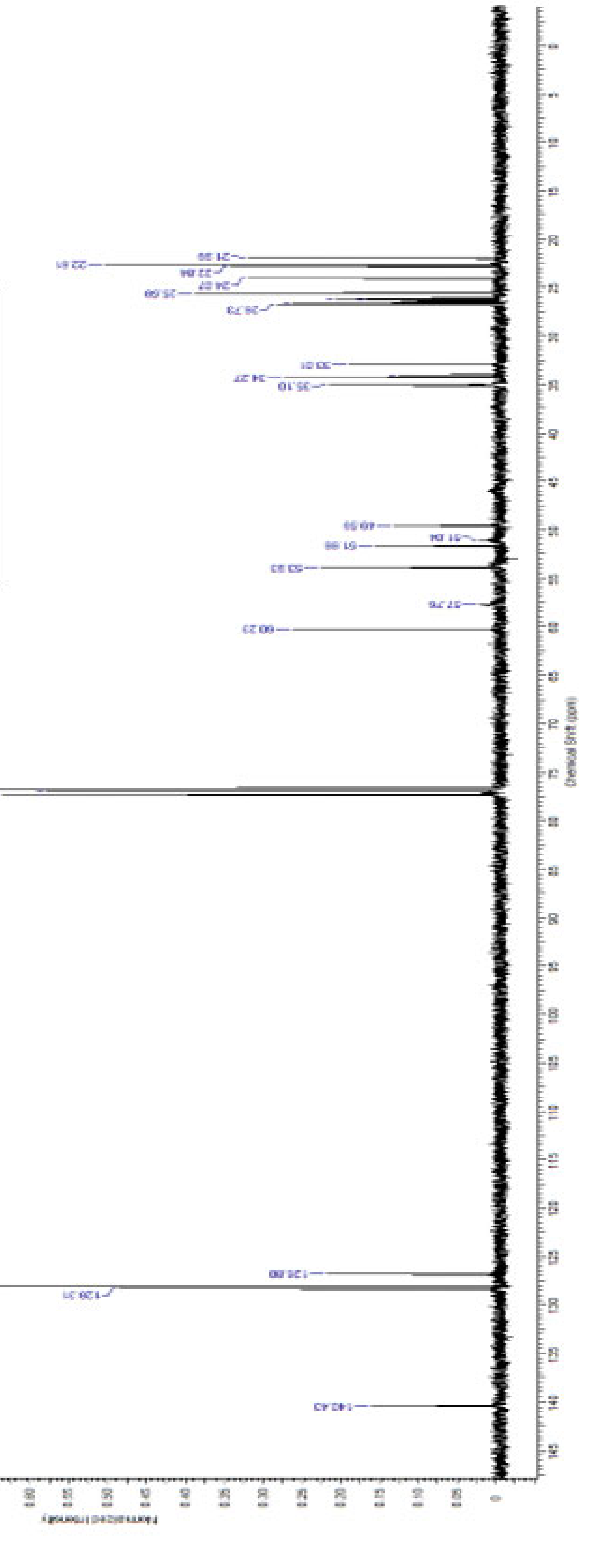

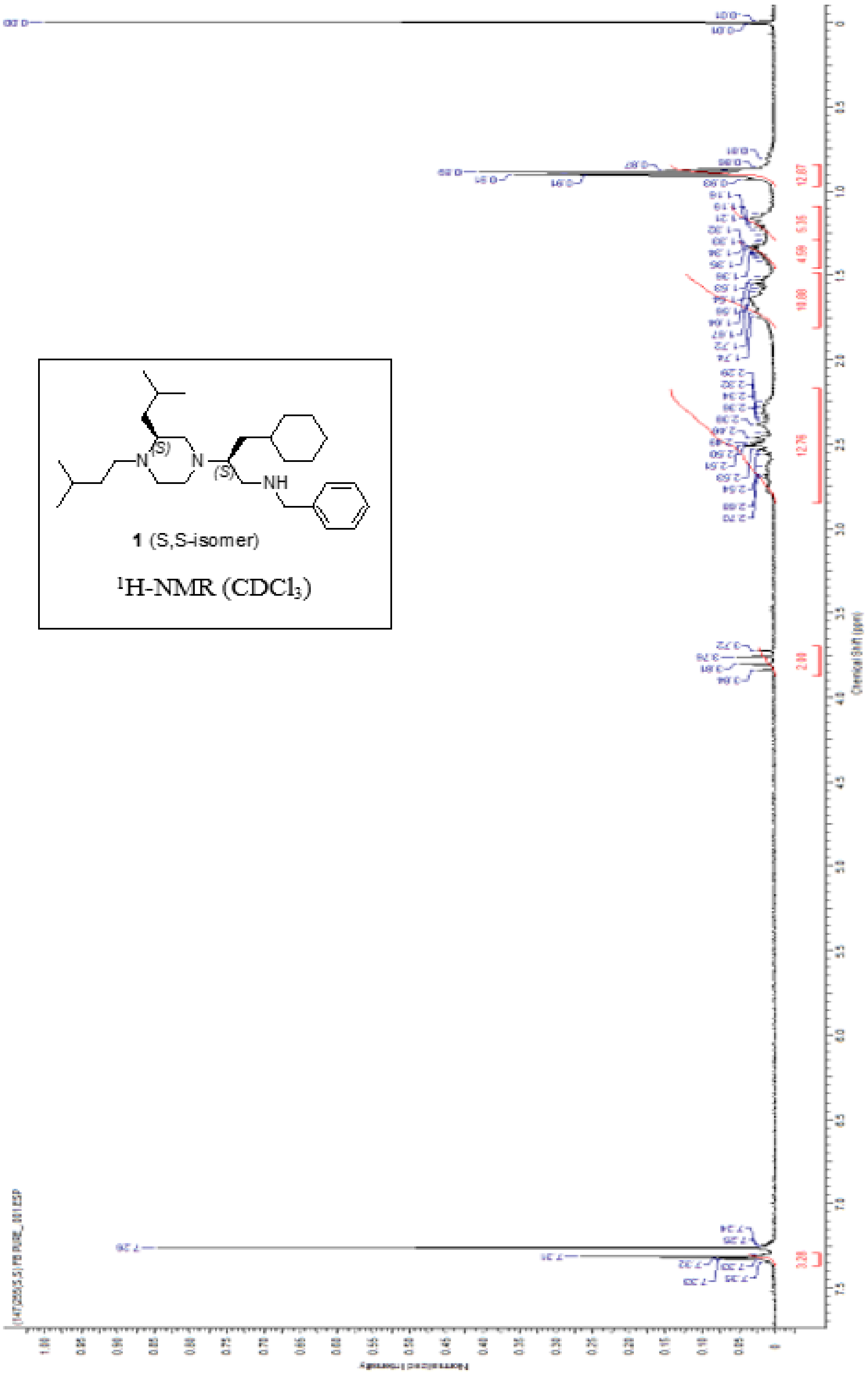

1 (S,S-isomer)

^{1}H-NMR ($CDCl_3$)

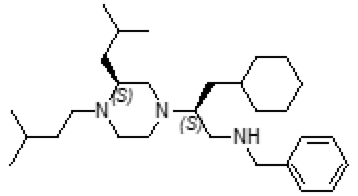

1 (S,S-isomer)

^{13}C-NMR ($CDCl_3$)

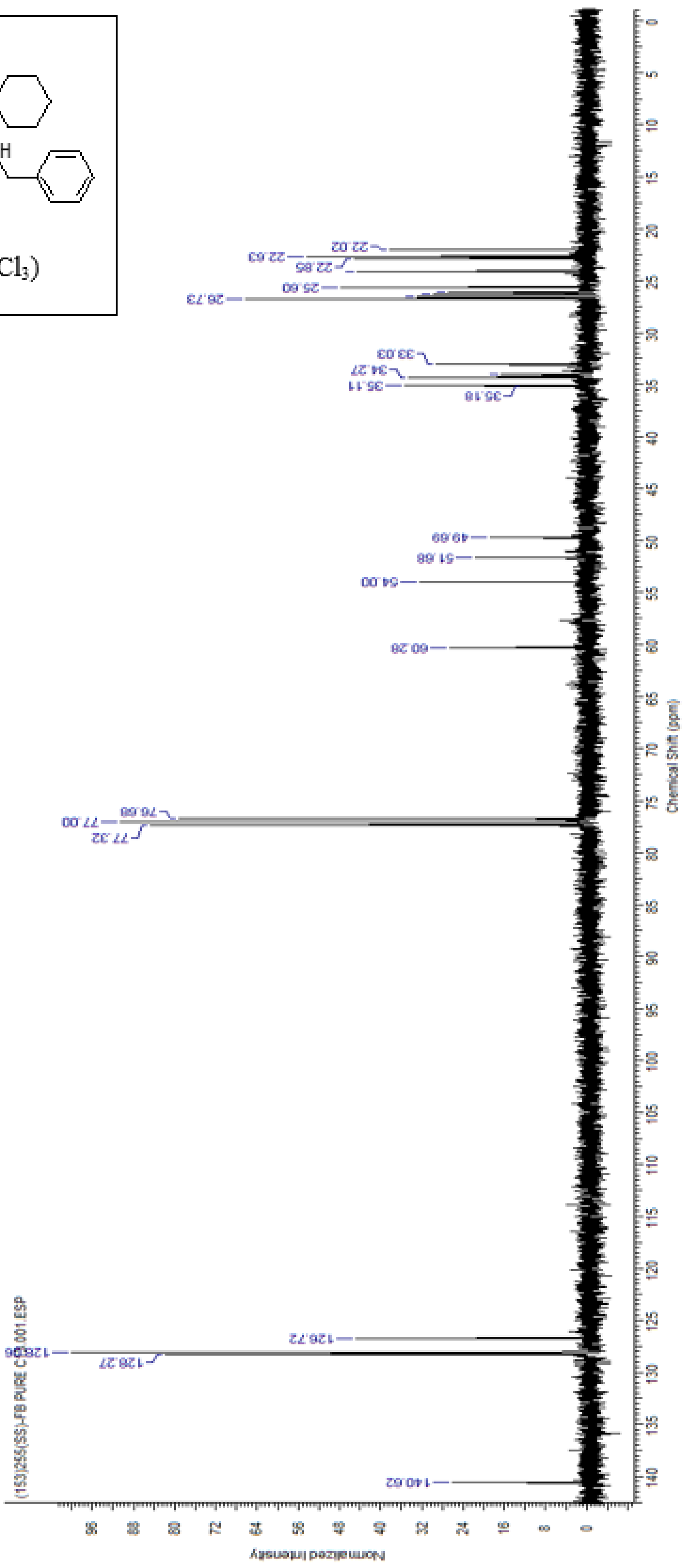

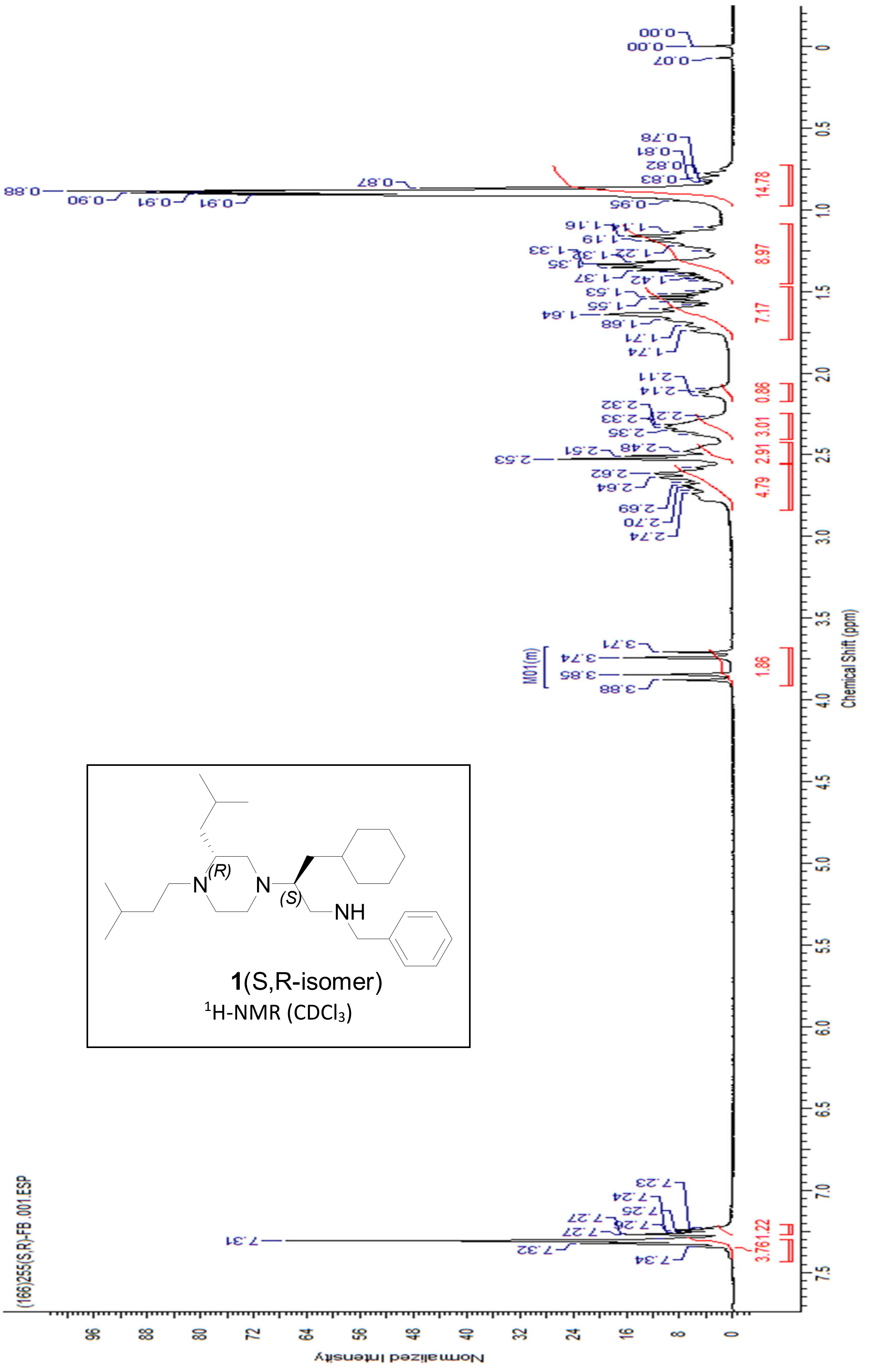

1(S,R-isomer)
^{1}H-NMR ($CDCl_3$)

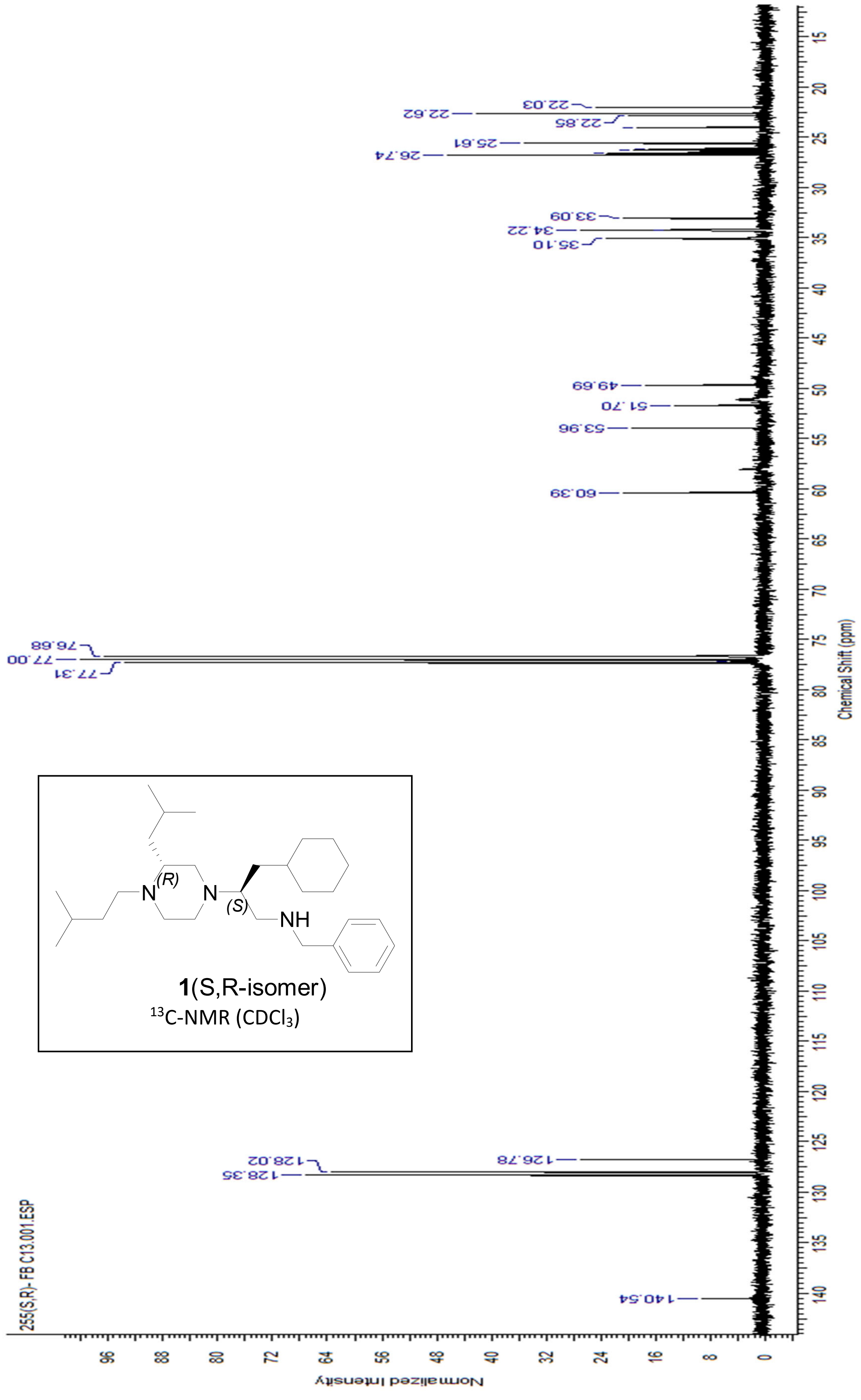
255(S,R)- FB C13.001.ESP
140.54
128.35
128.02
126.78
77.31
77.00
76.68
60.39
53.96
51.70
49.69
35.10
34.22
33.09
26.74
25.61
22.85
22.62
22.03
Chemical Shift (ppm)
Normalized Intensity
(R)
(S)
N
N
NH
1(S,R-isomer)
13C-NMR (CDCl3)

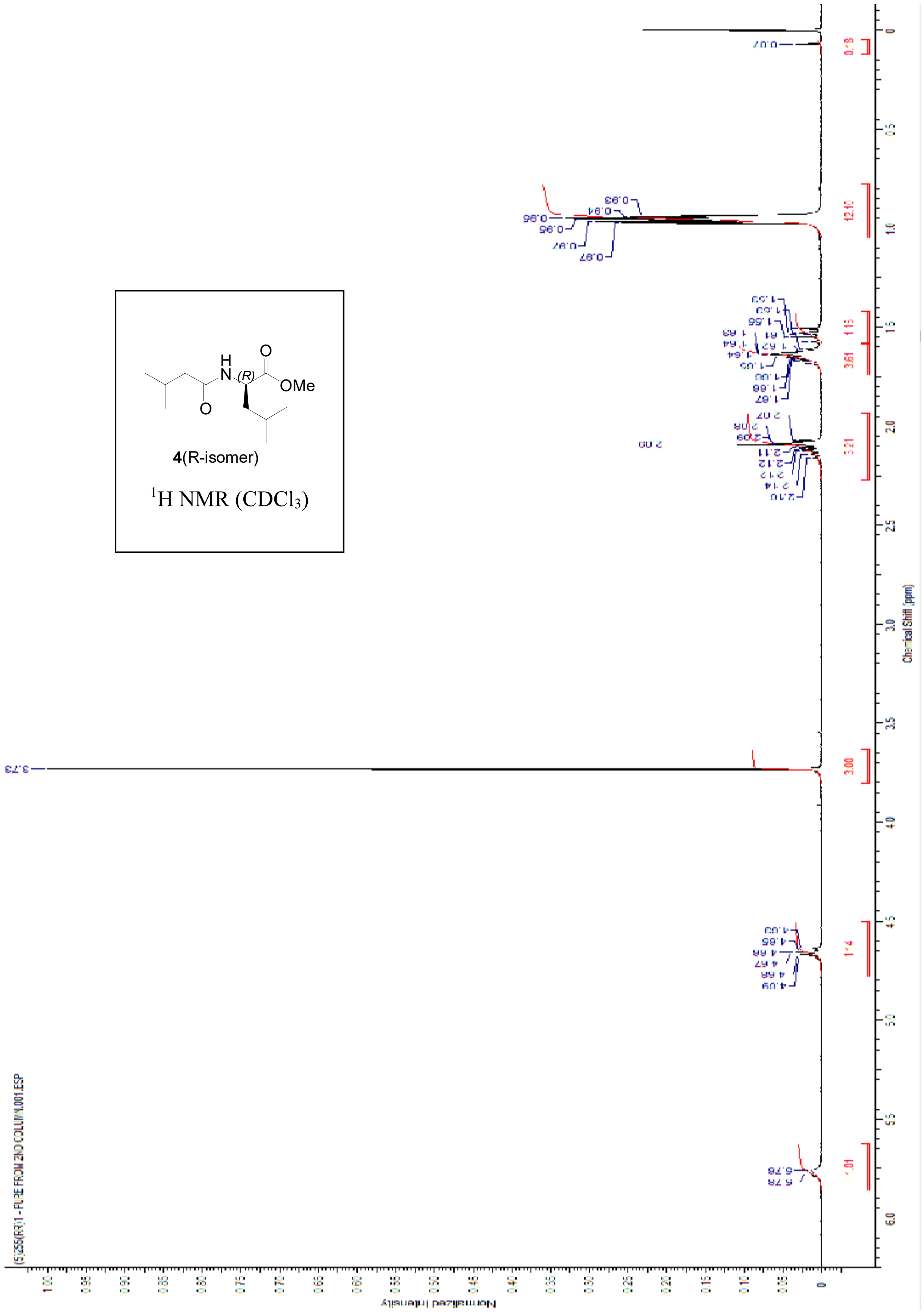

OMe
4(R-isomer)
1H NMR (CDCl3)
Chemical Shift (ppm)
Normalized Intensity

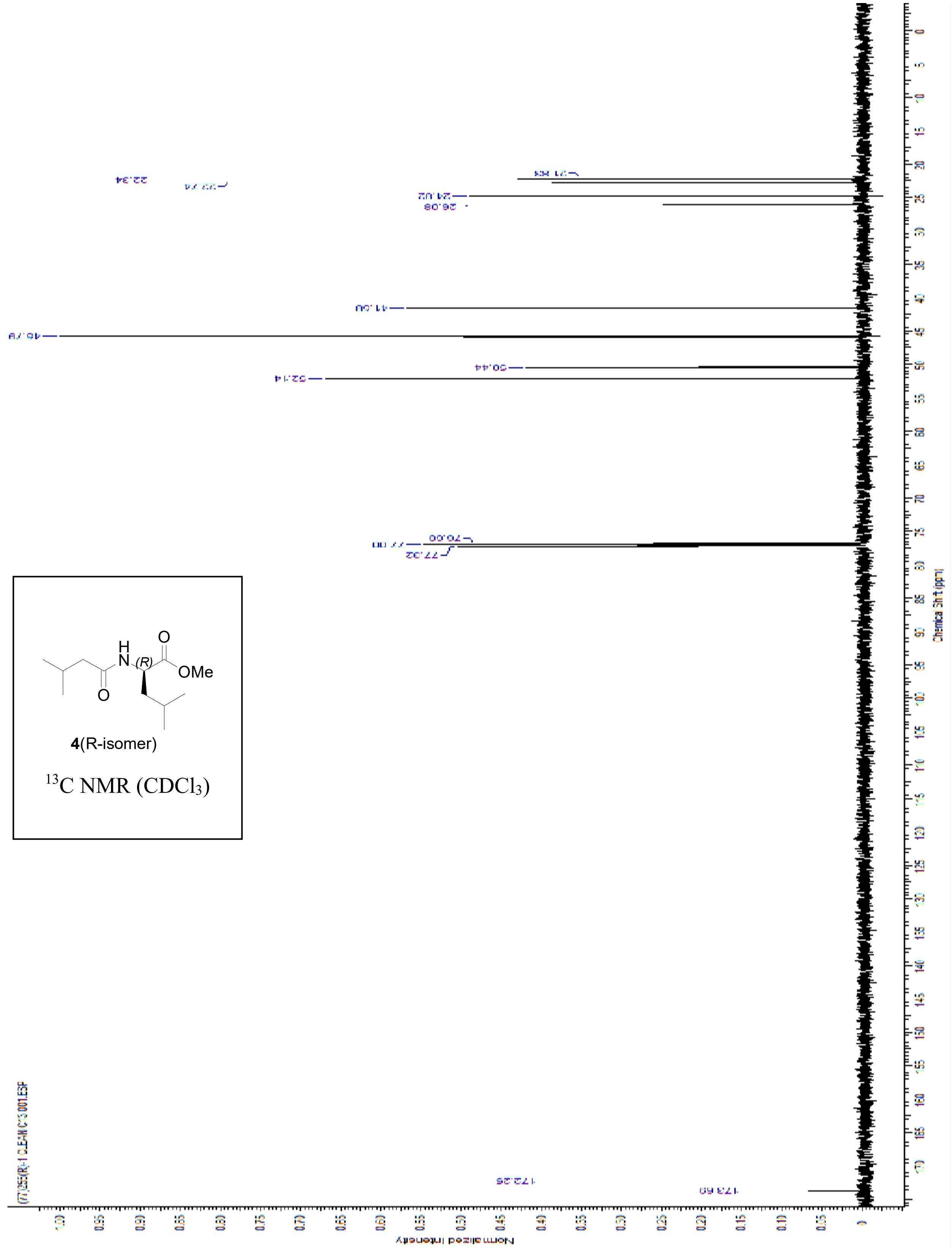

4(R-isomer)

^{13}C NMR ($CDCl_3$)

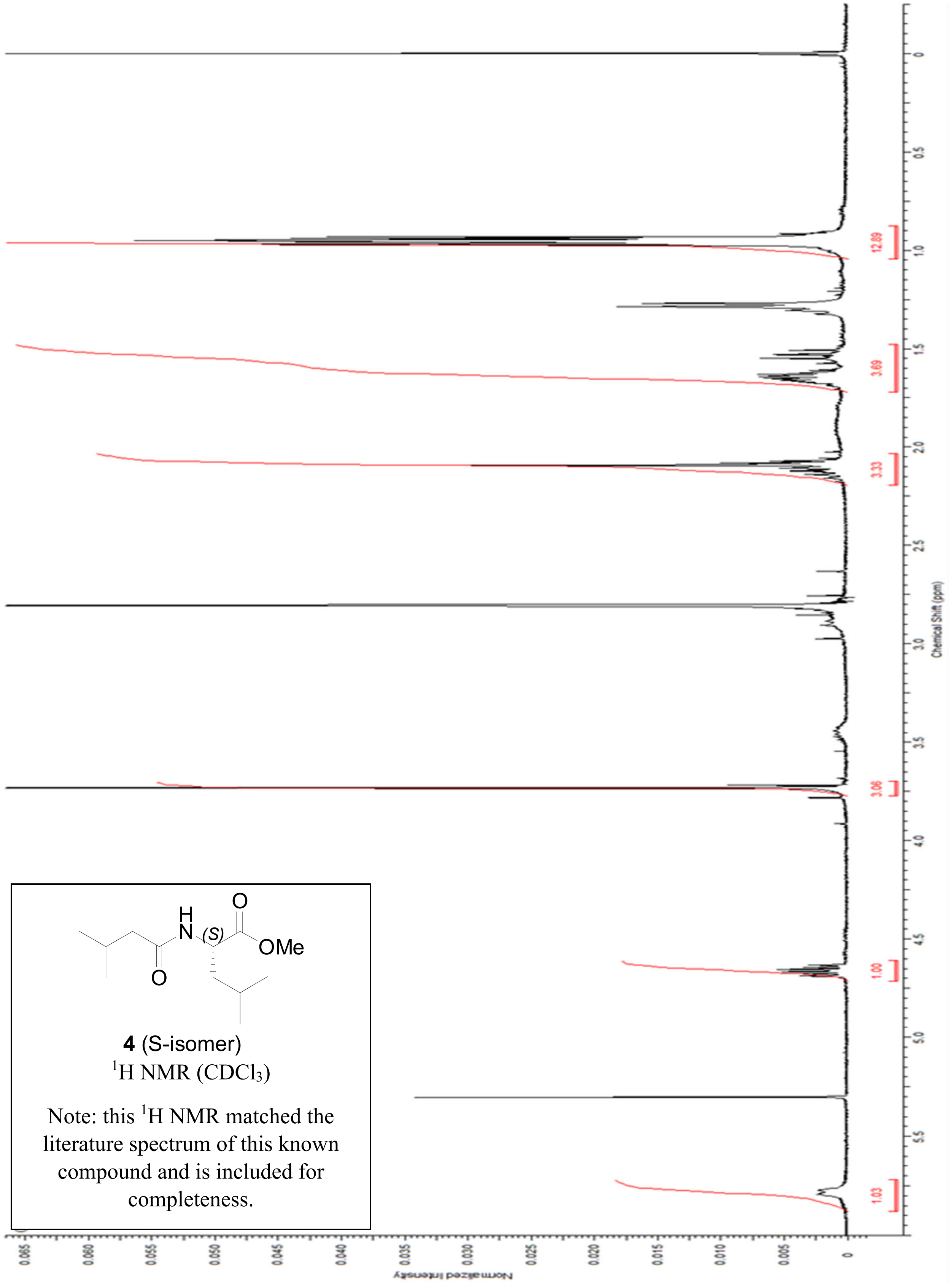

4 (S-isomer)

^{1}H NMR ($CDCl_3$)

Note: this ^{1}H NMR matched the literature spectrum of this known compound and is included for completeness.

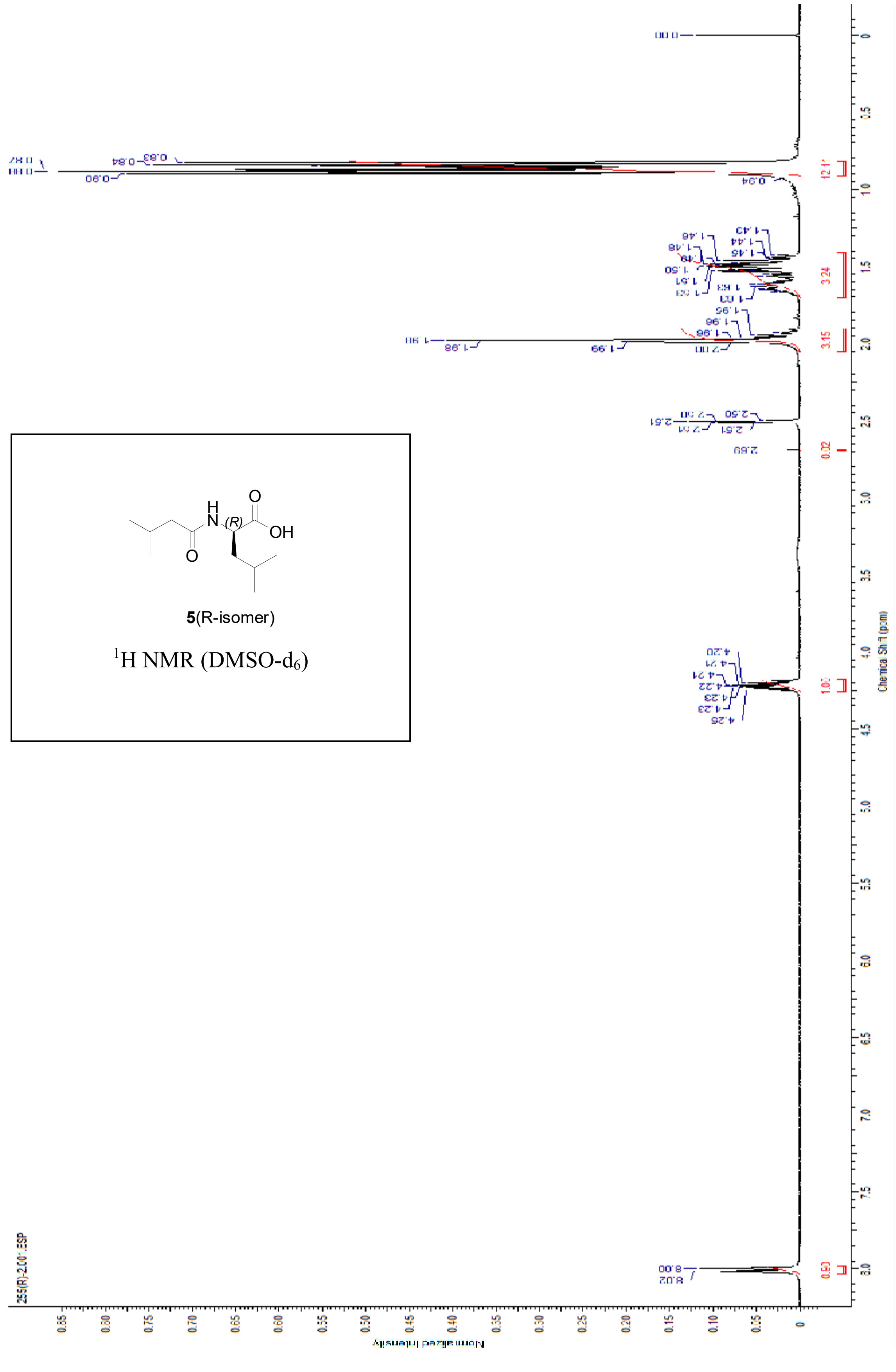

5(R-isomer)

^{1}H NMR (DMSO-d_6)

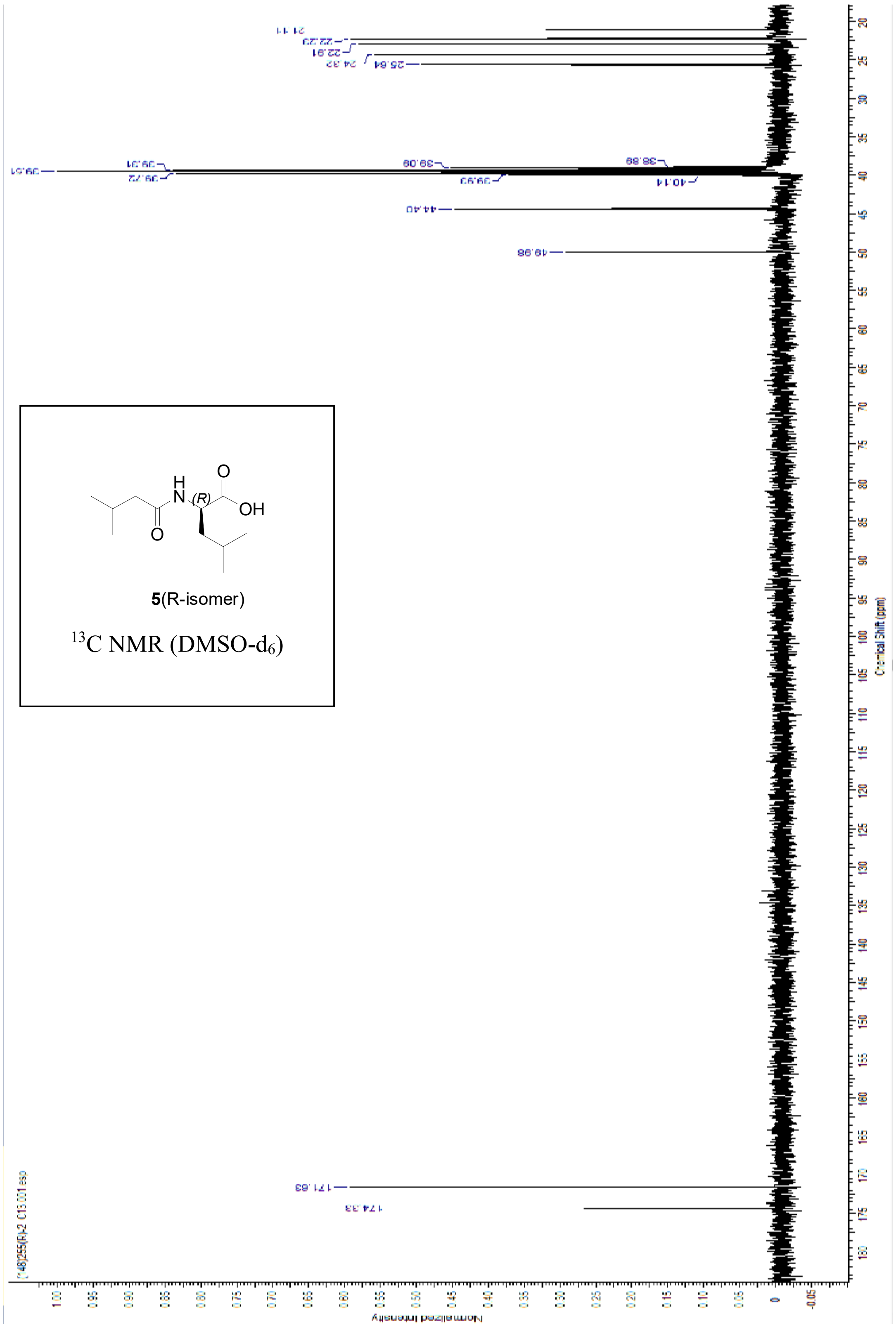

5(R-isomer)

^{13}C NMR (DMSO-d$_6$)

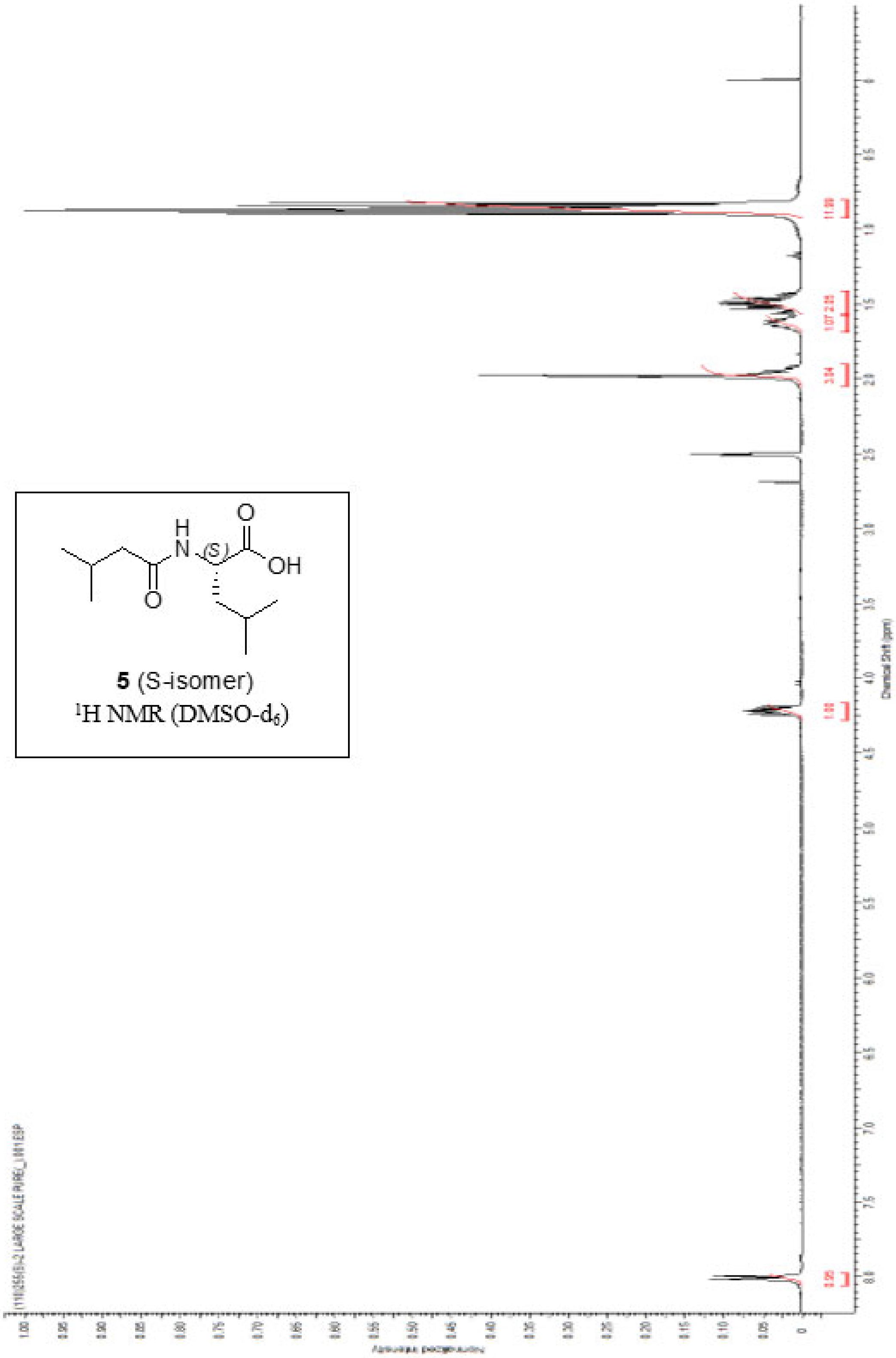
5 (S-isomer)
1H NMR (DMSO-d6)

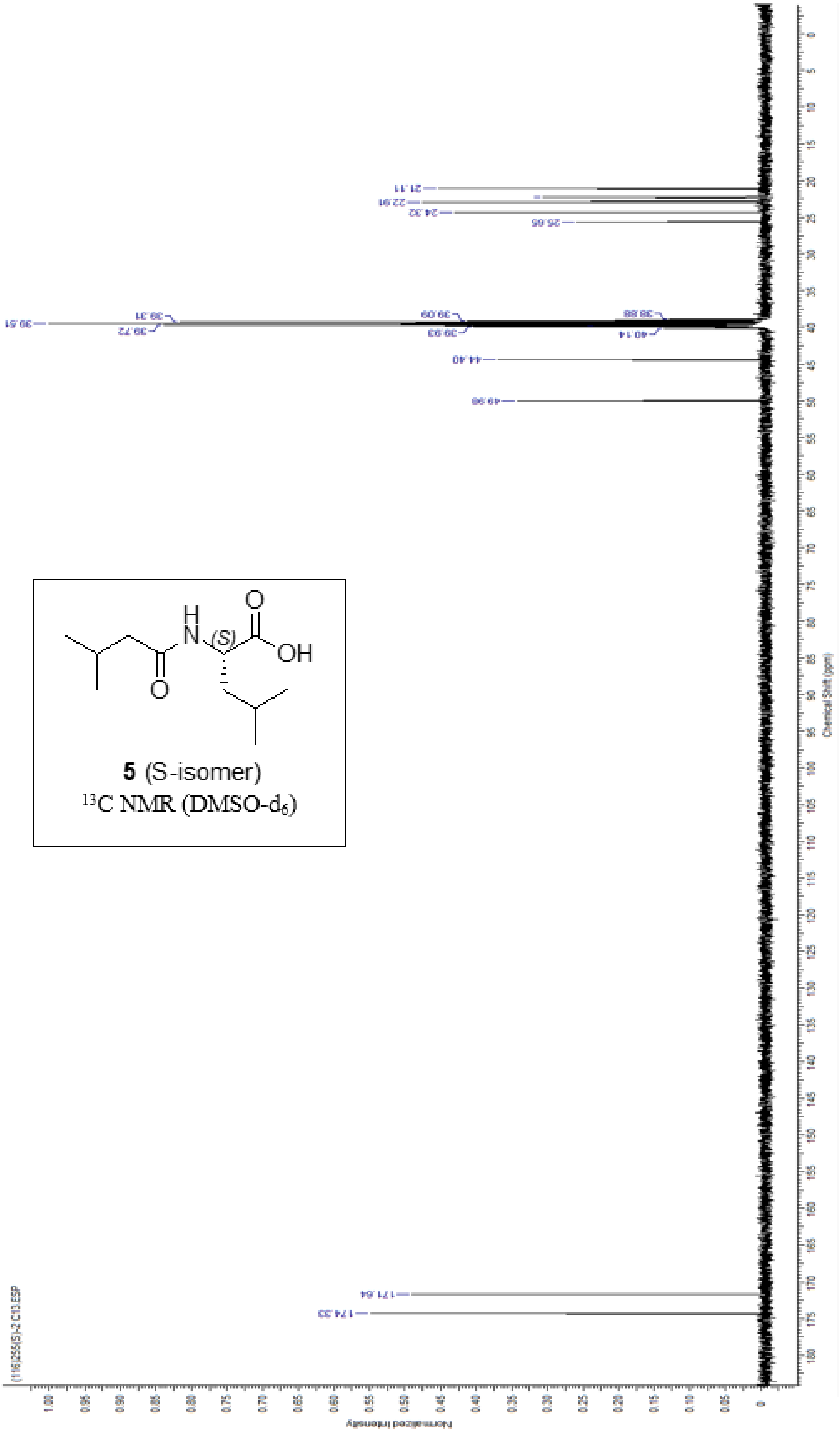

O
H
N
(S)
OH
O
5 (S-isomer)
^{13}C NMR (DMSO-d_6)
21.11
22.91
24.32
25.65
39.31
39.51
39.72
39.09
39.93
38.88
40.14
44.40
49.98
171.64
174.33
Chemical Shift (ppm)
Normalized Intensity

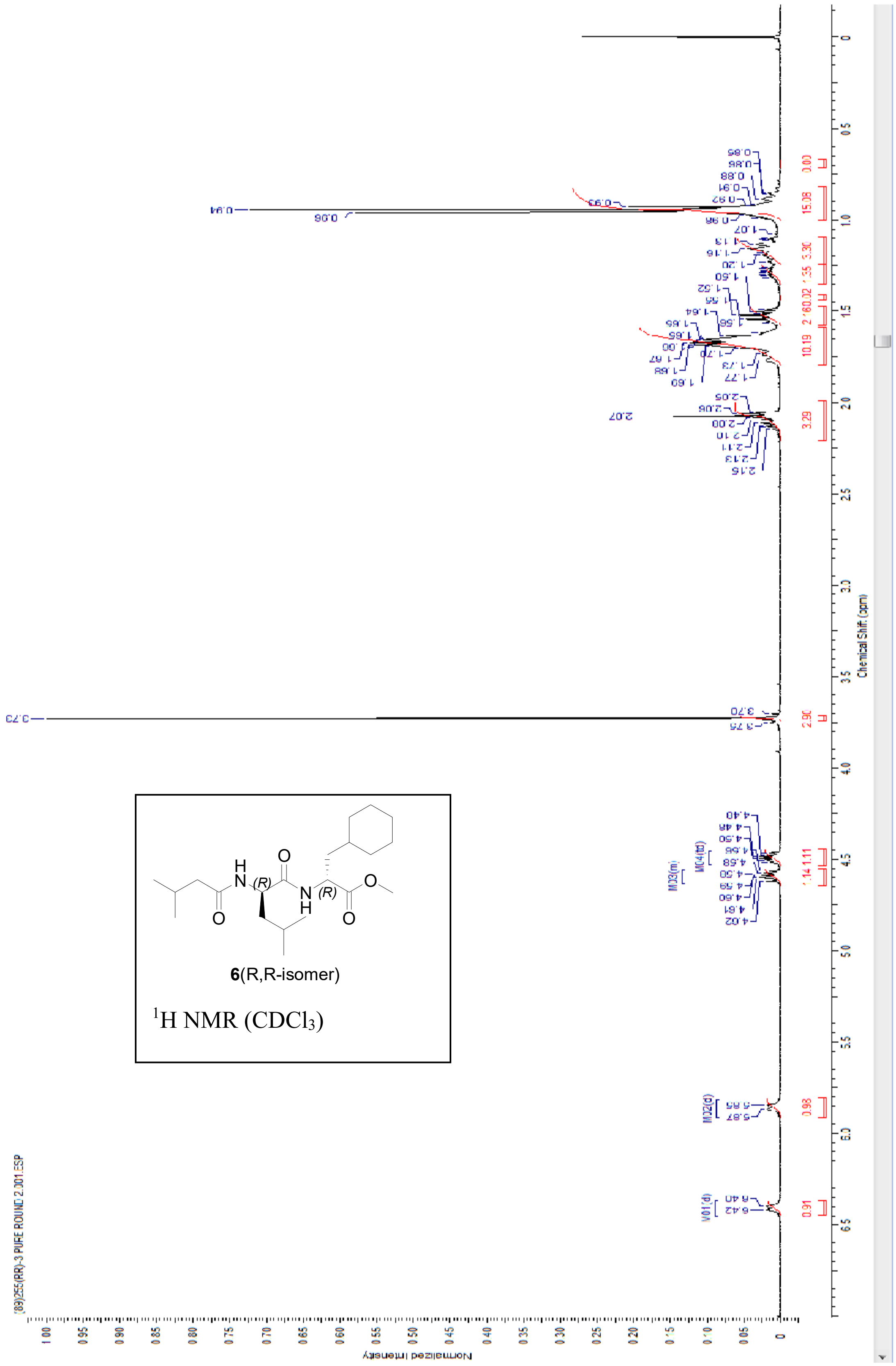

6(R,R-isomer)

^{1}H NMR ($CDCl_3$)

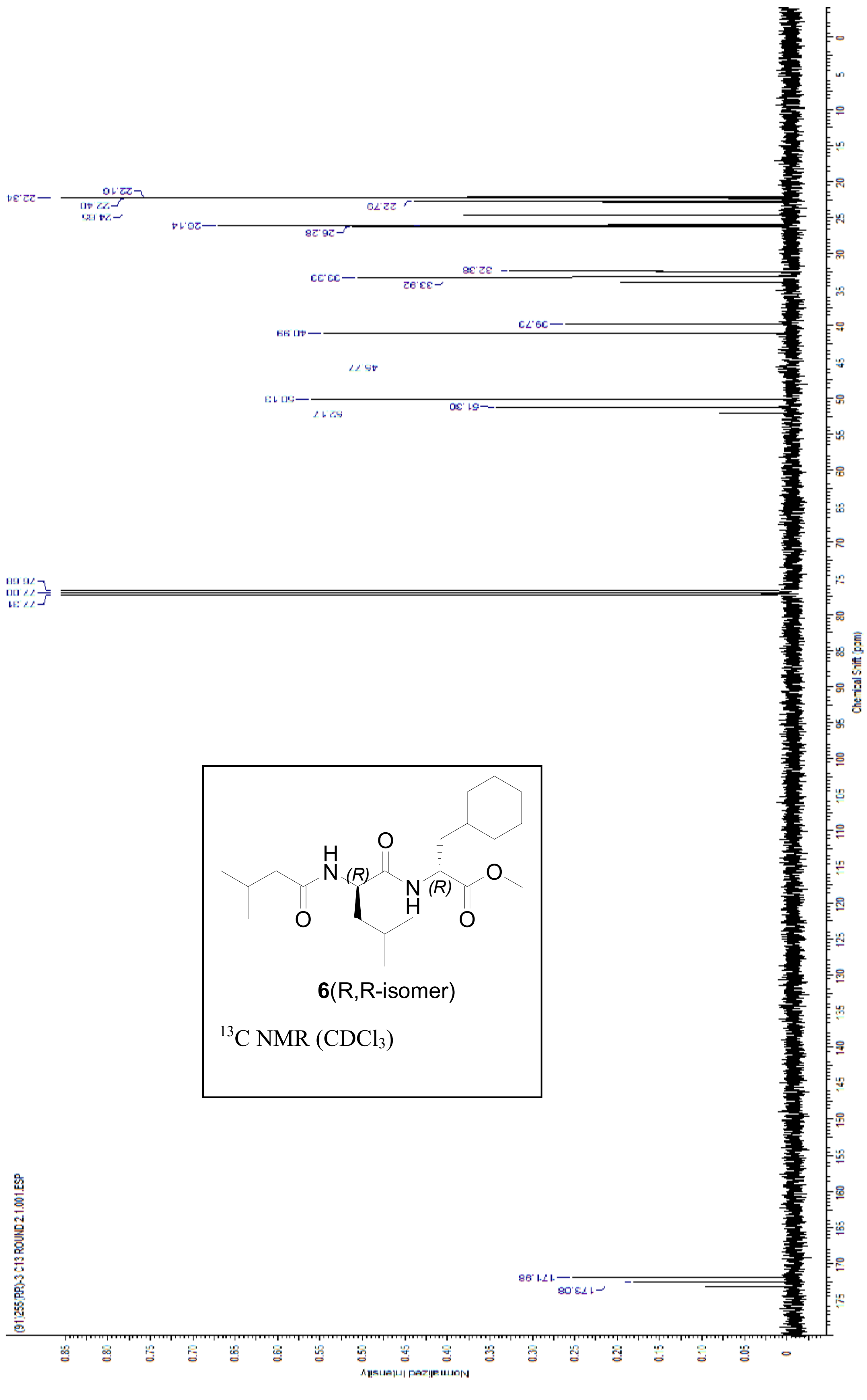
6(R,R-isomer)
^{13}C NMR ($CDCl_3$)
Chemical Shift (ppm)
Normalized Intensity

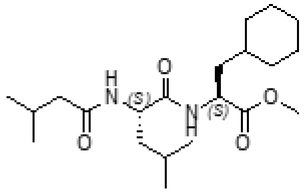

6 (S,S-isomer)

^{1}H NMR ($CDCl_3$)

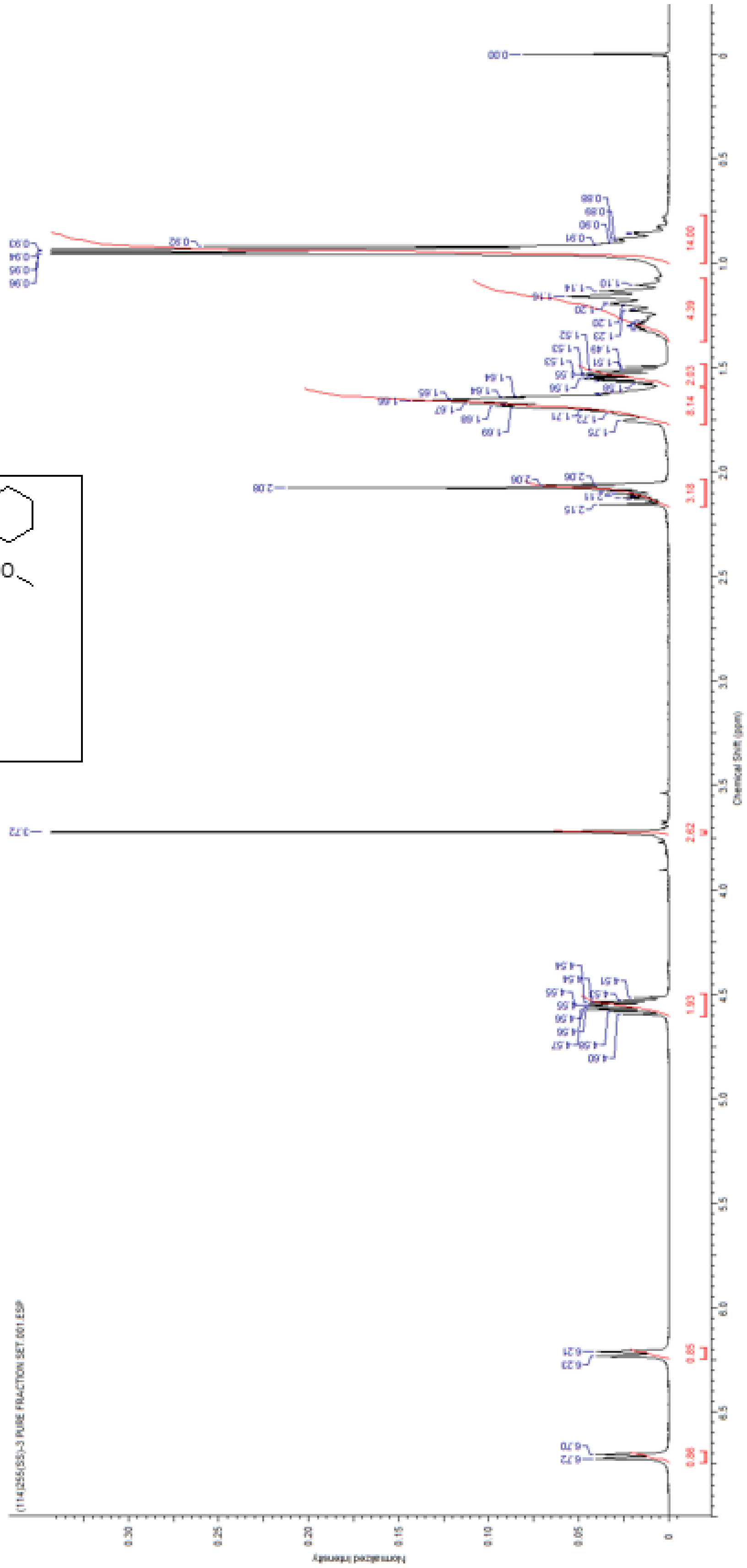

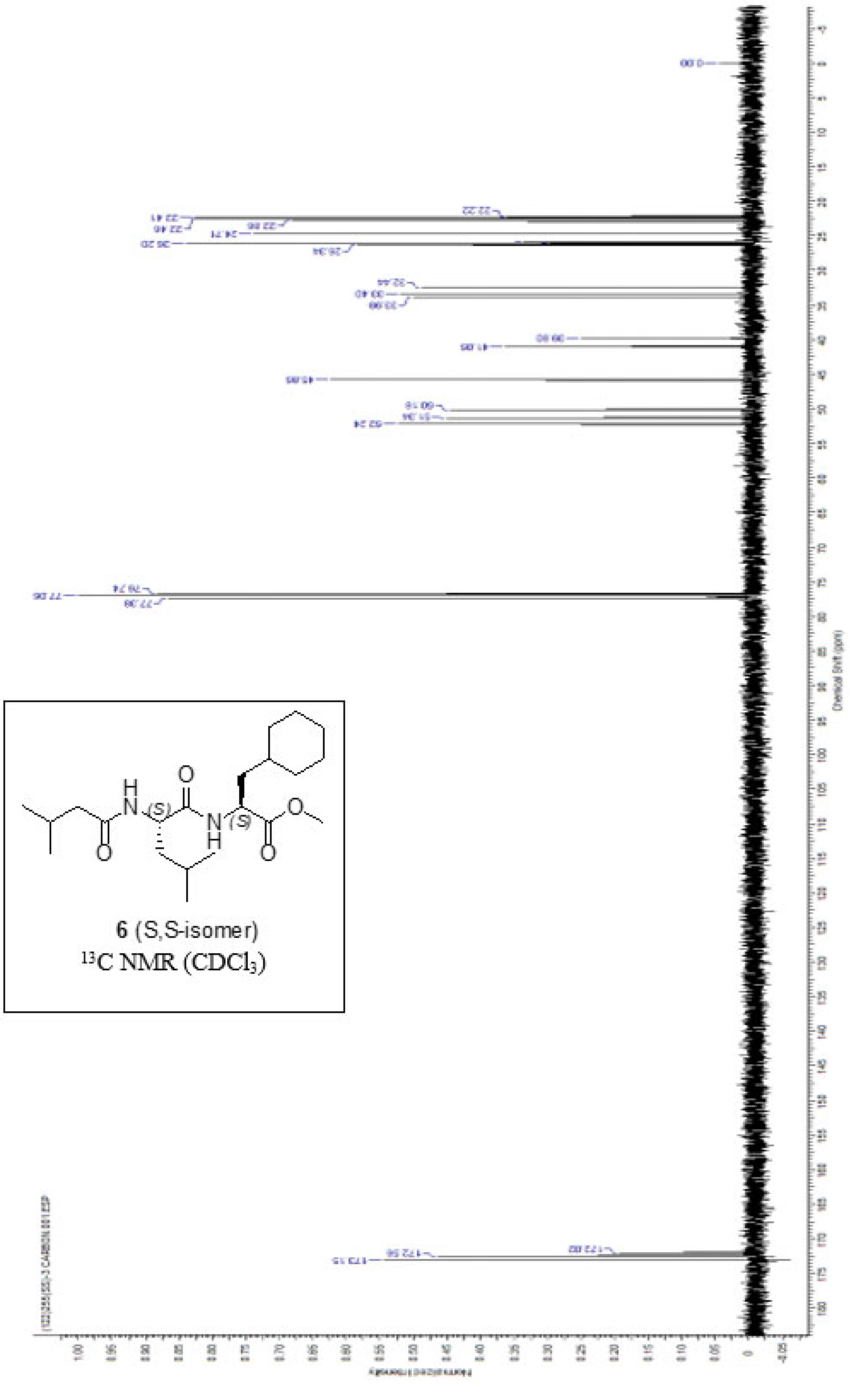

6 (S,S-isomer)
^{13}C NMR ($CDCl_3$)

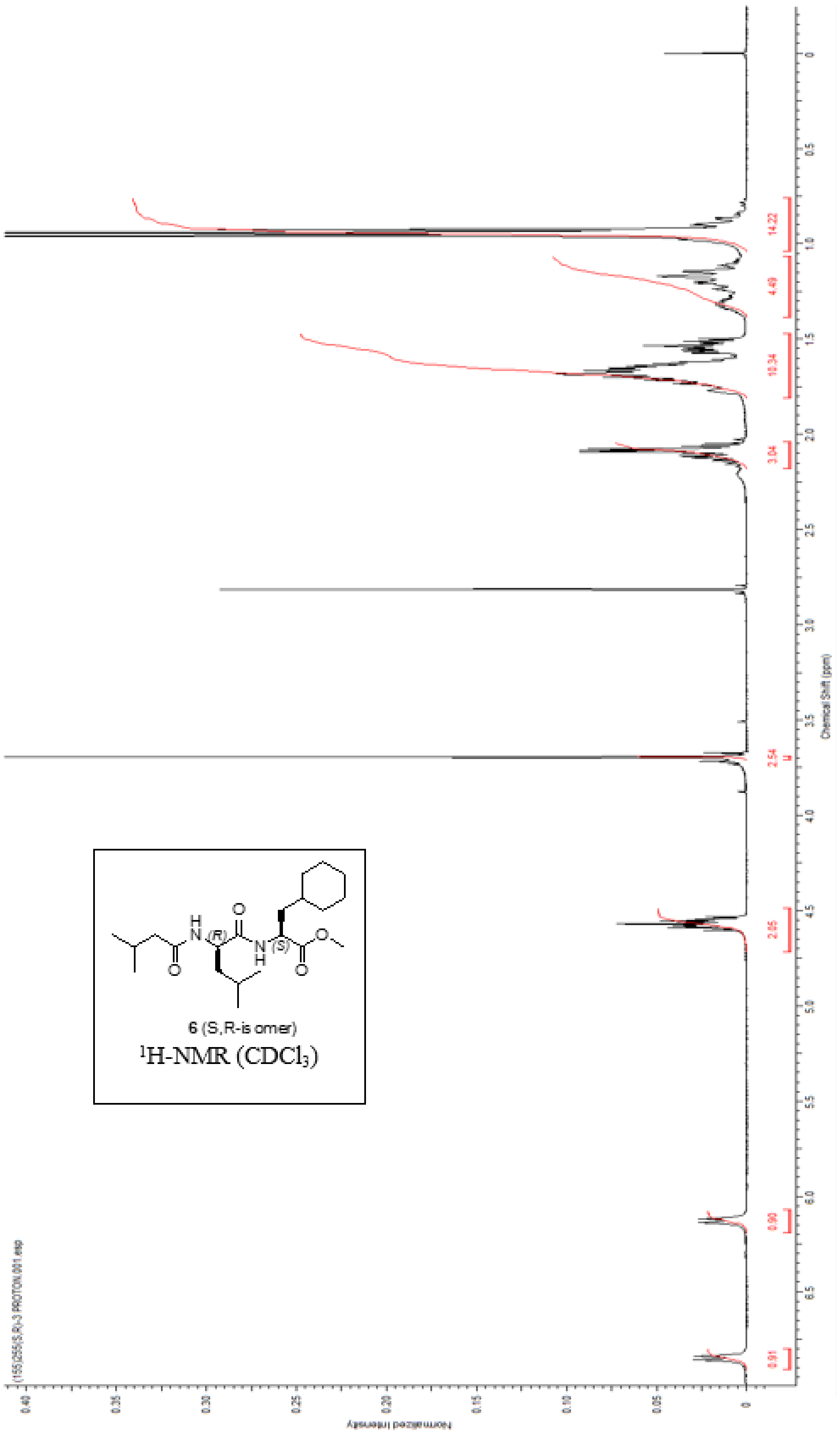

6 (S,R-is omer)
1H-NMR (CDCl3)
14.22
4.49
10.34
3.04
2.54
2.05
0.90
0.91
Chemical Shift (ppm)
Normalized Intensity

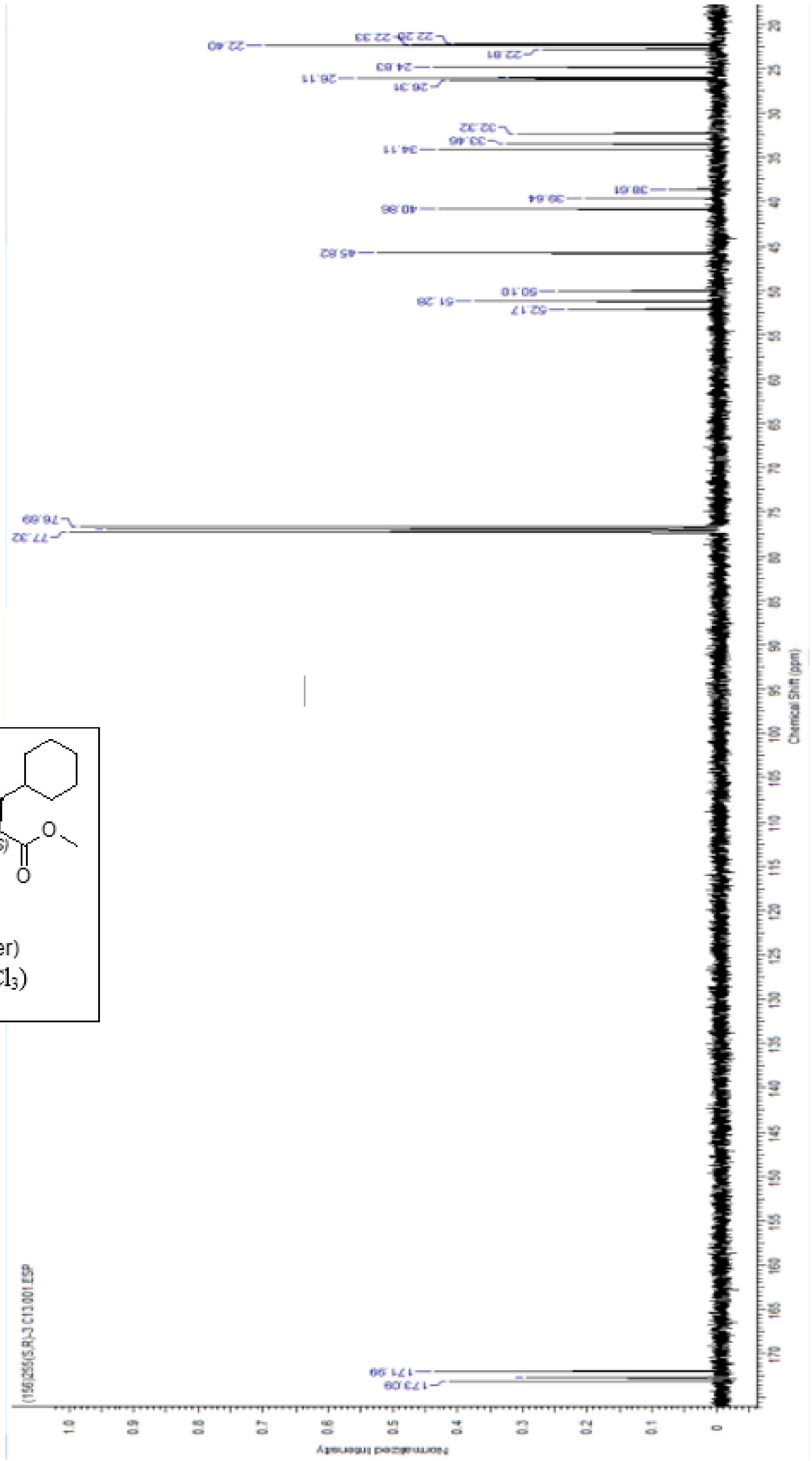

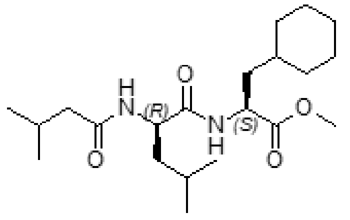

6 (S,R-isomer)
^{13}C-NMR ($CDCl_3$)

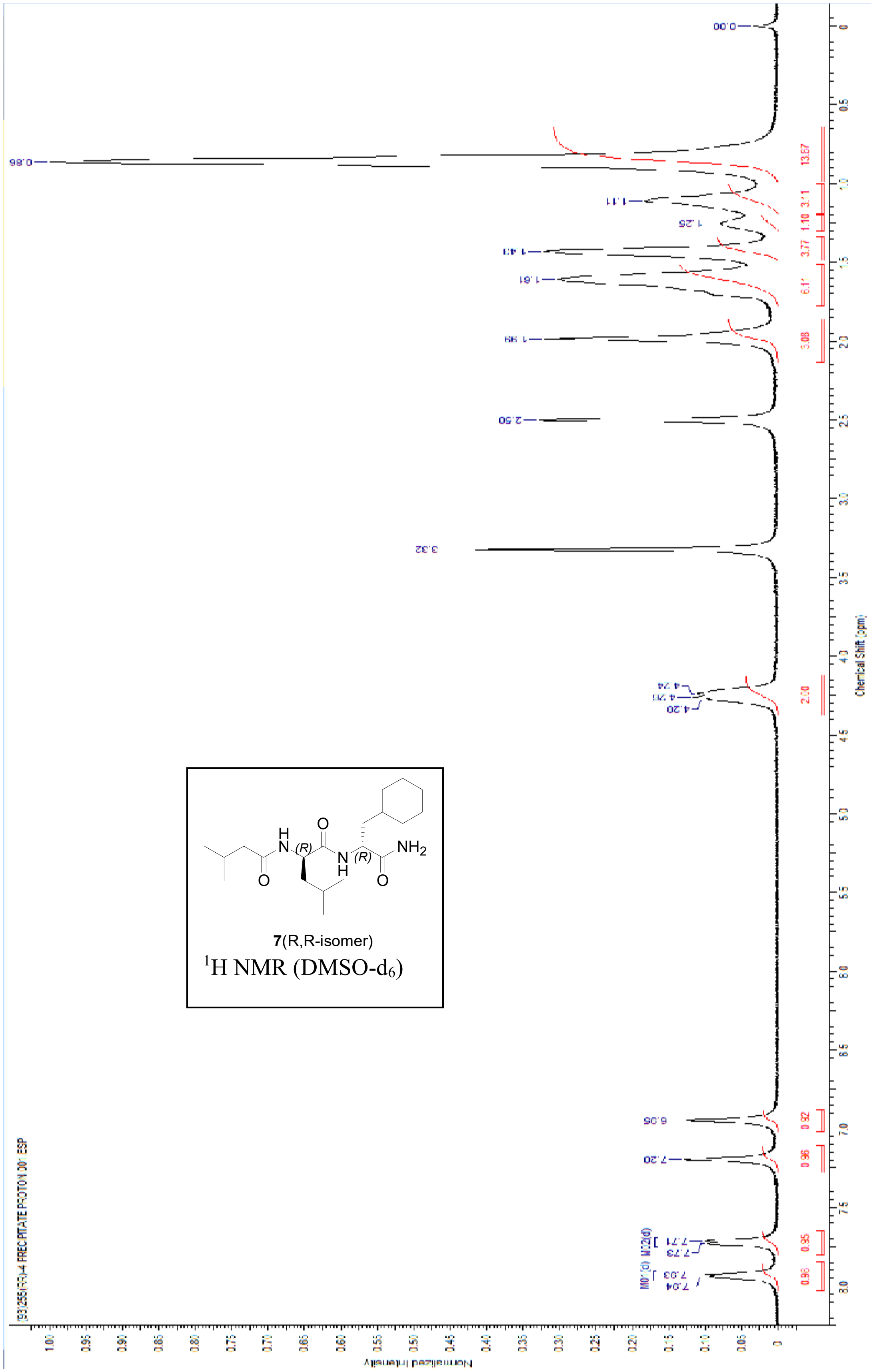
7(R,R-isomer)
1H NMR (DMSO-d6)

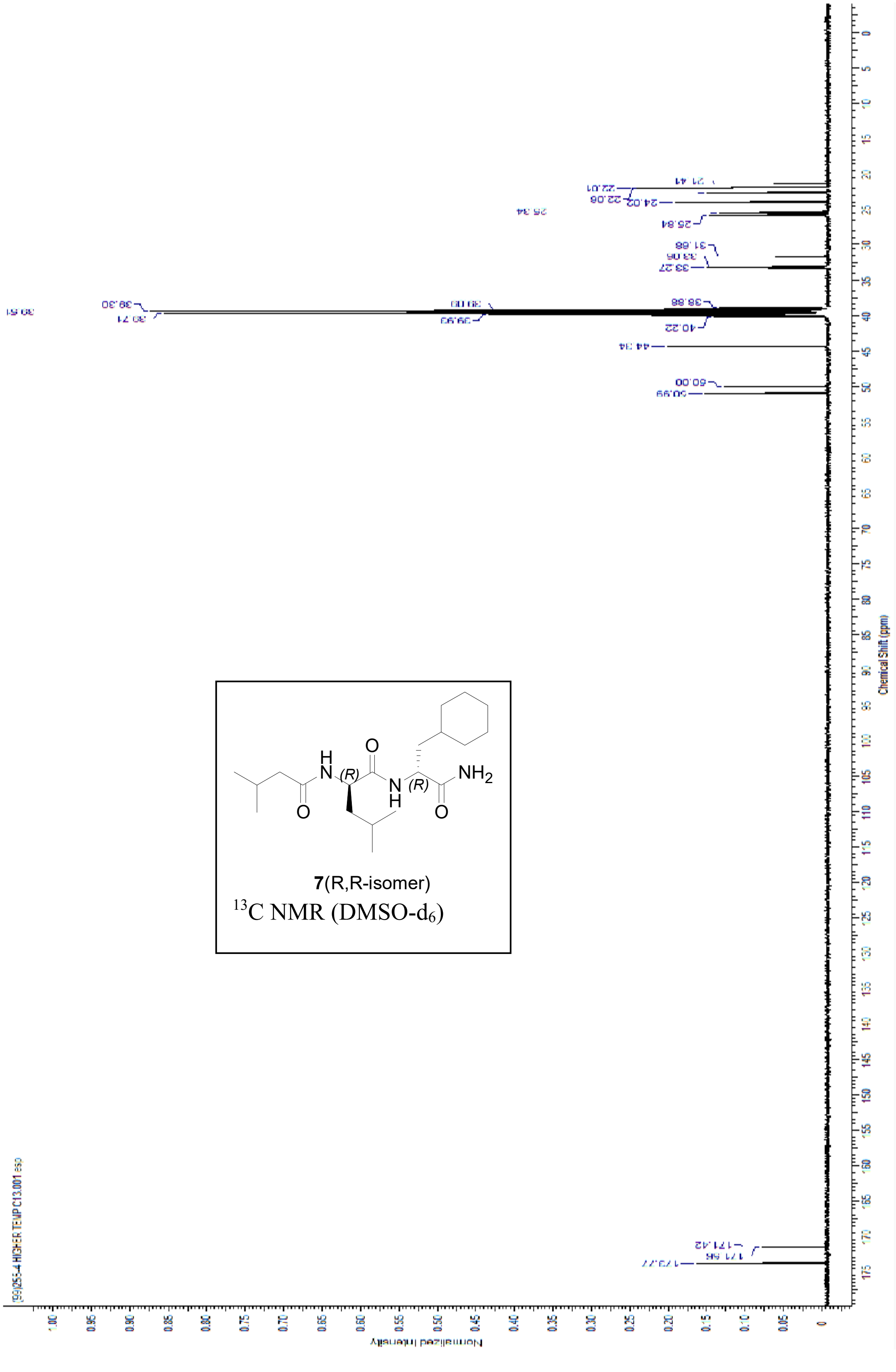
7(R,R-isomer)
^{13}C NMR (DMSO-d_6)

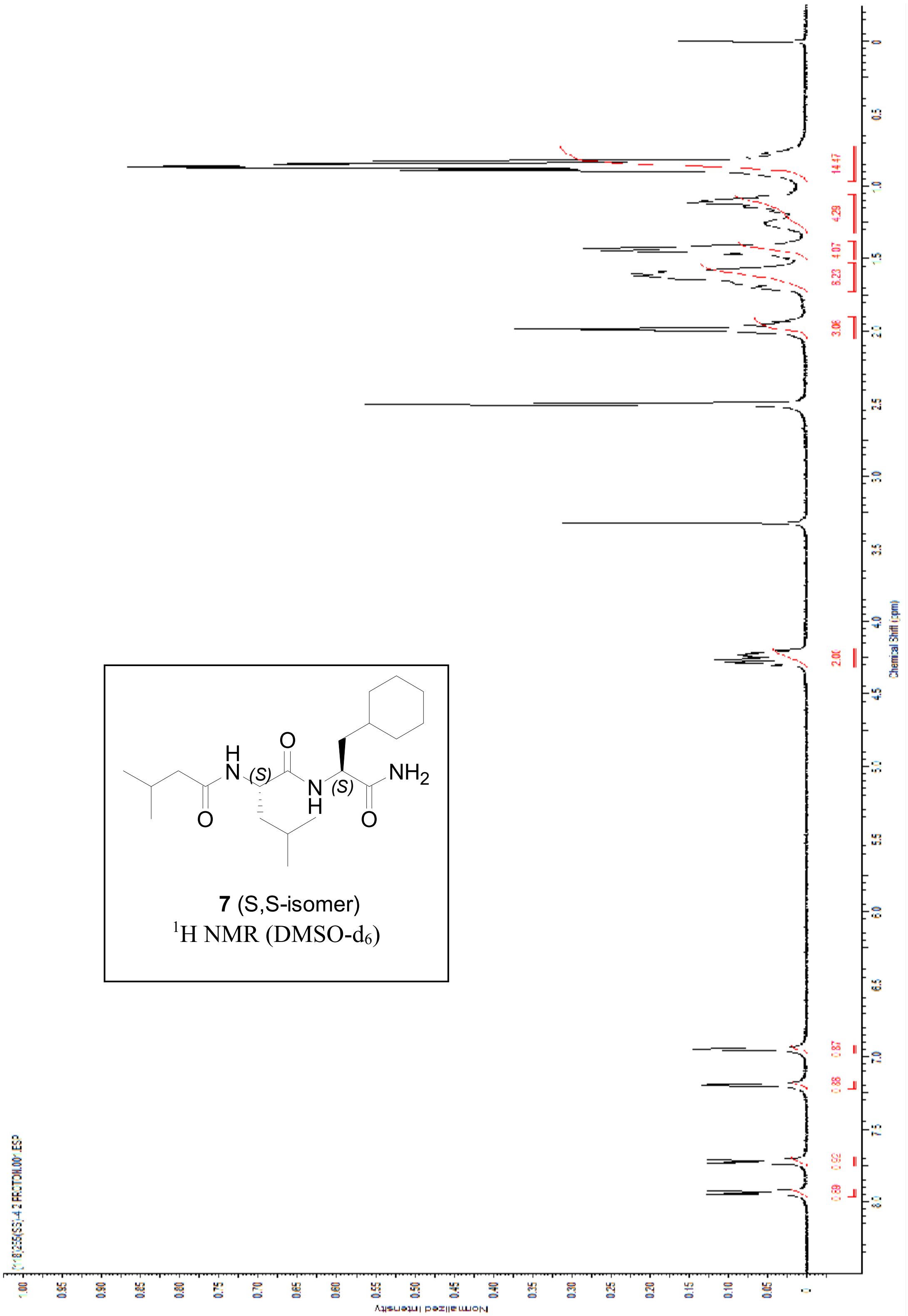

7 (S,S-isomer)

^{1}H NMR (DMSO-d_6)

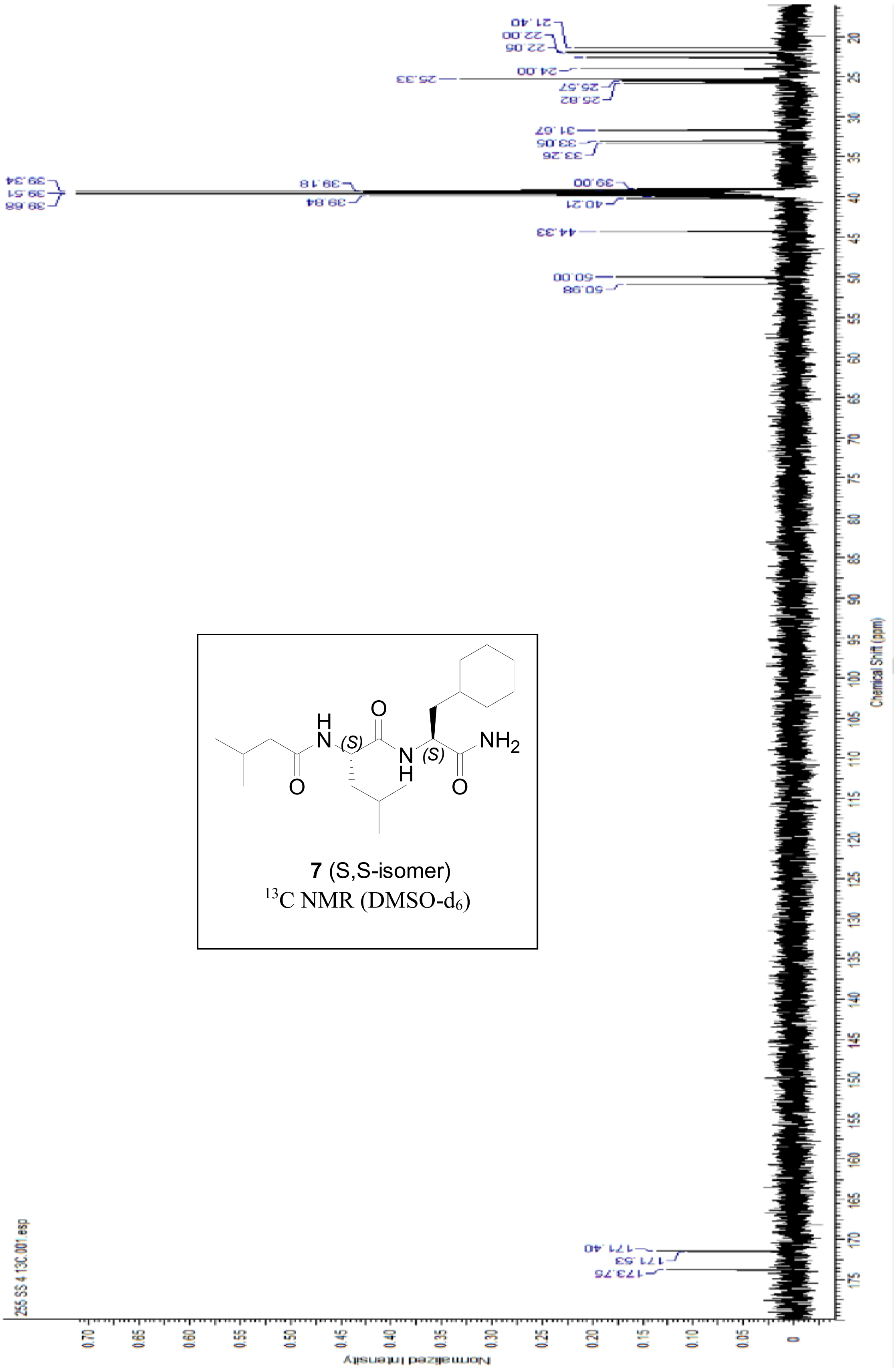

7 (S,S-isomer)

^{13}C NMR (DMSO-d_6)

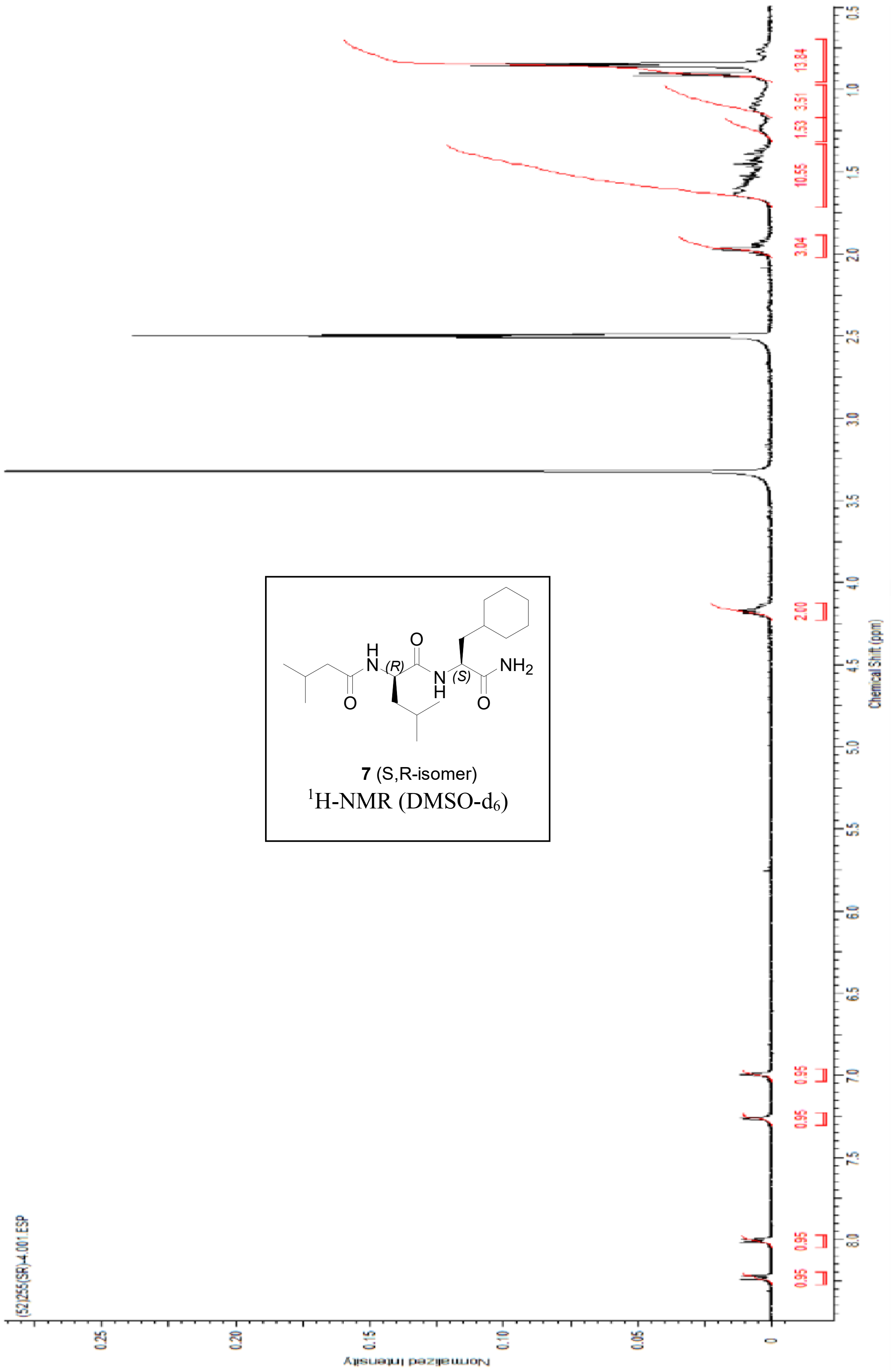

7 (S,R-isomer)
1H-NMR (DMSO-d6)
(52)255(SR)-4.001.ESP
Normalized Intensity
Chemical Shift (ppm)
13.84
3.51
1.53
10.55
3.04
2.00
0.95
0.95
0.95
0.95

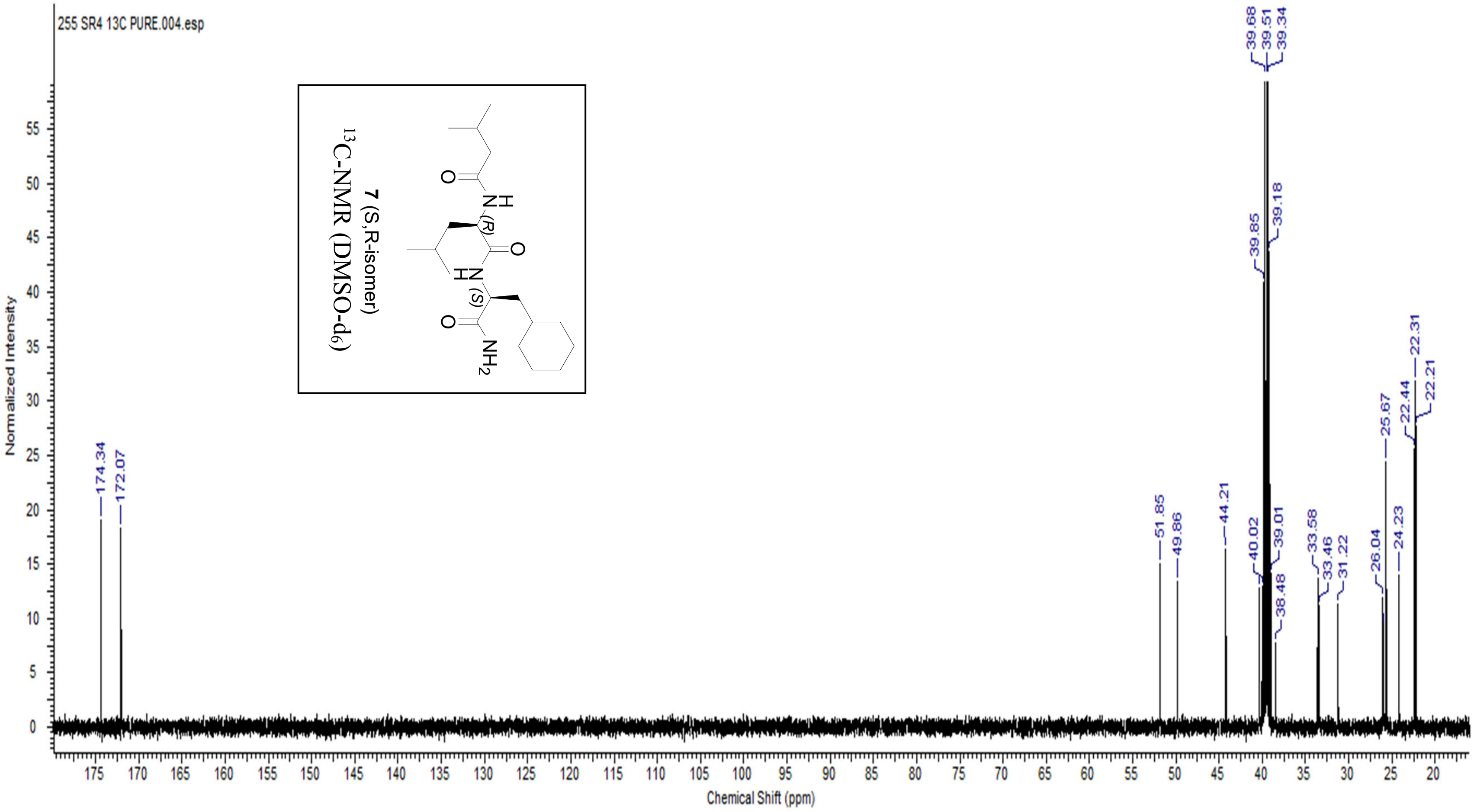

7 (S,R-isomer)

^{13}C-NMR (DMSO-d_6)

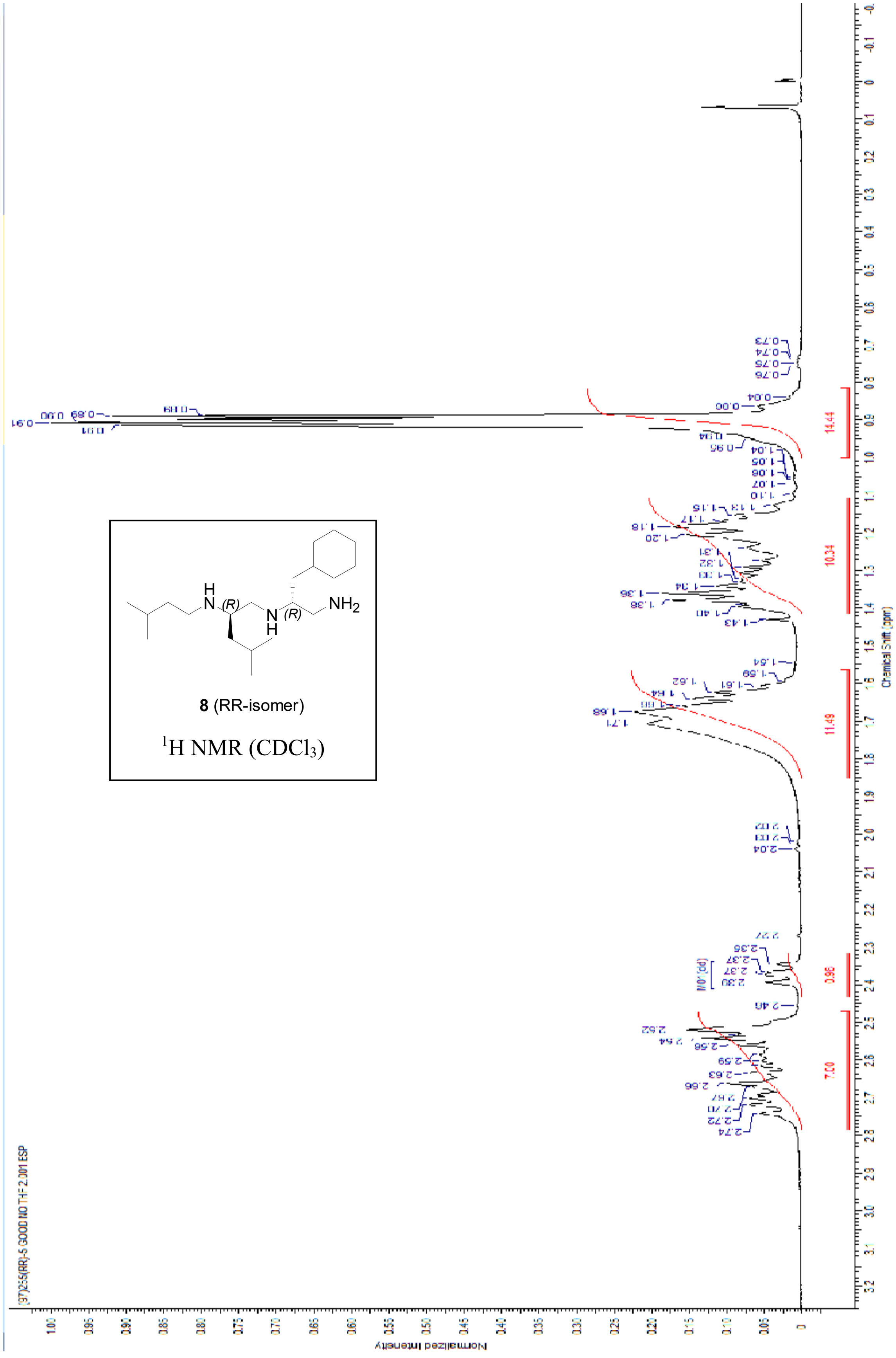

8 (RR-isomer)

^{1}H NMR ($CDCl_3$)

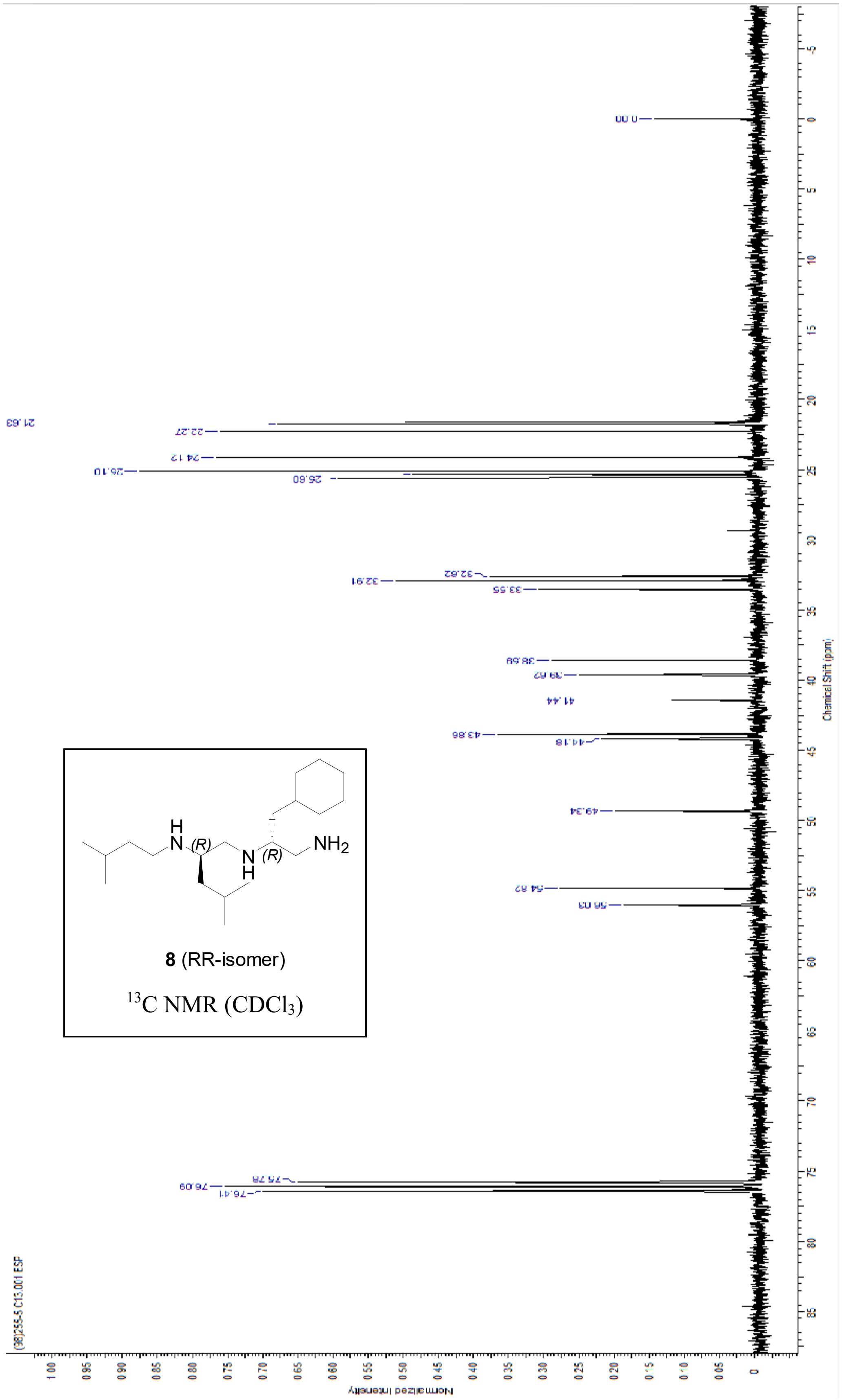
8 (RR-isomer)
^{13}C NMR ($CDCl_3$)
(R)
(R)
NH2
0.00
21.63
22.27
24.12
25.10
25.60
32.82
32.91
33.55
38.59
39.62
41.44
43.86
44.18
49.34
54.82
56.03
75.78
76.09
76.41
Chemical Shift (ppm)
Normalized Intensity

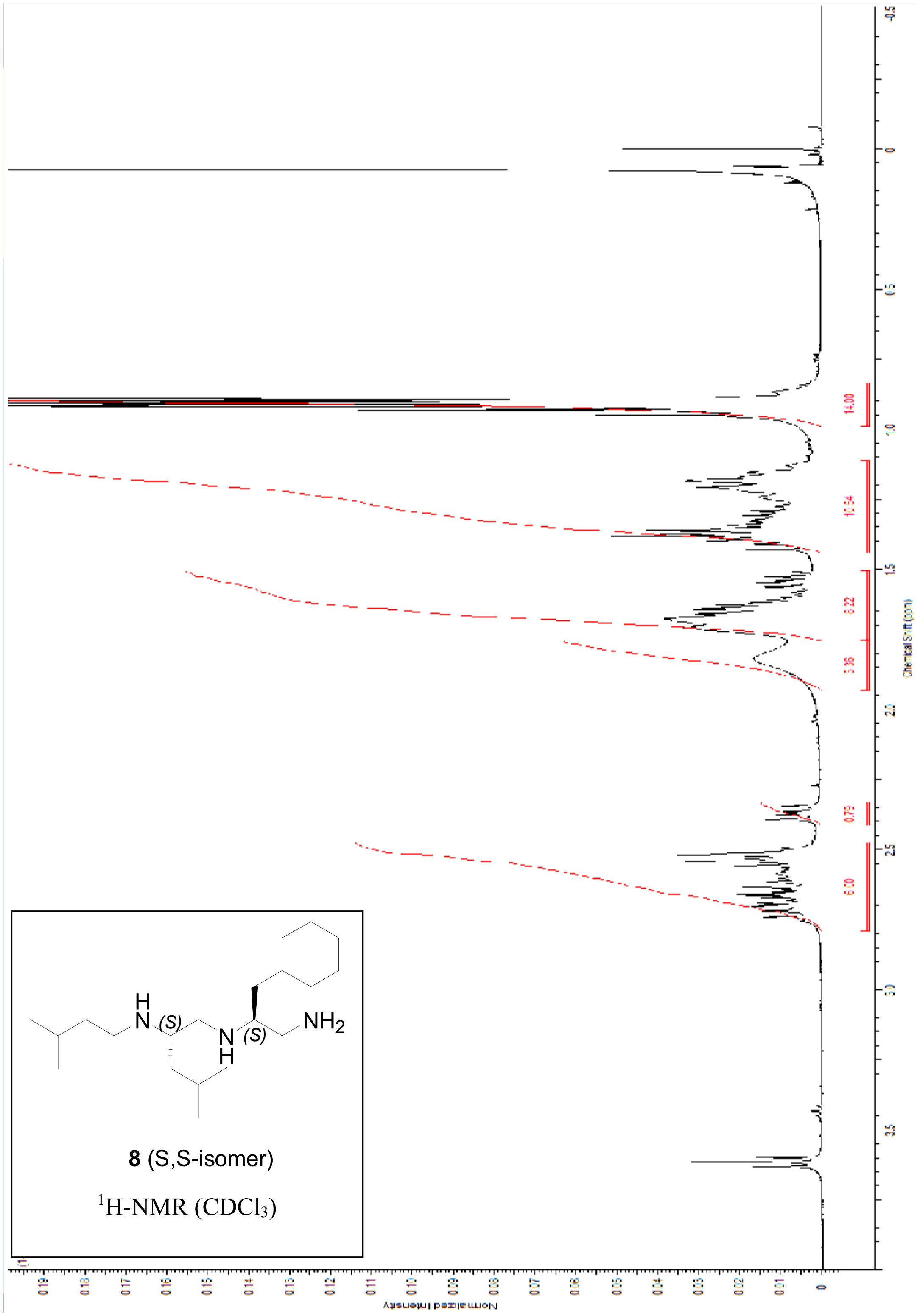

8 (S,S-isomer)

^{1}H-NMR ($CDCl_3$)

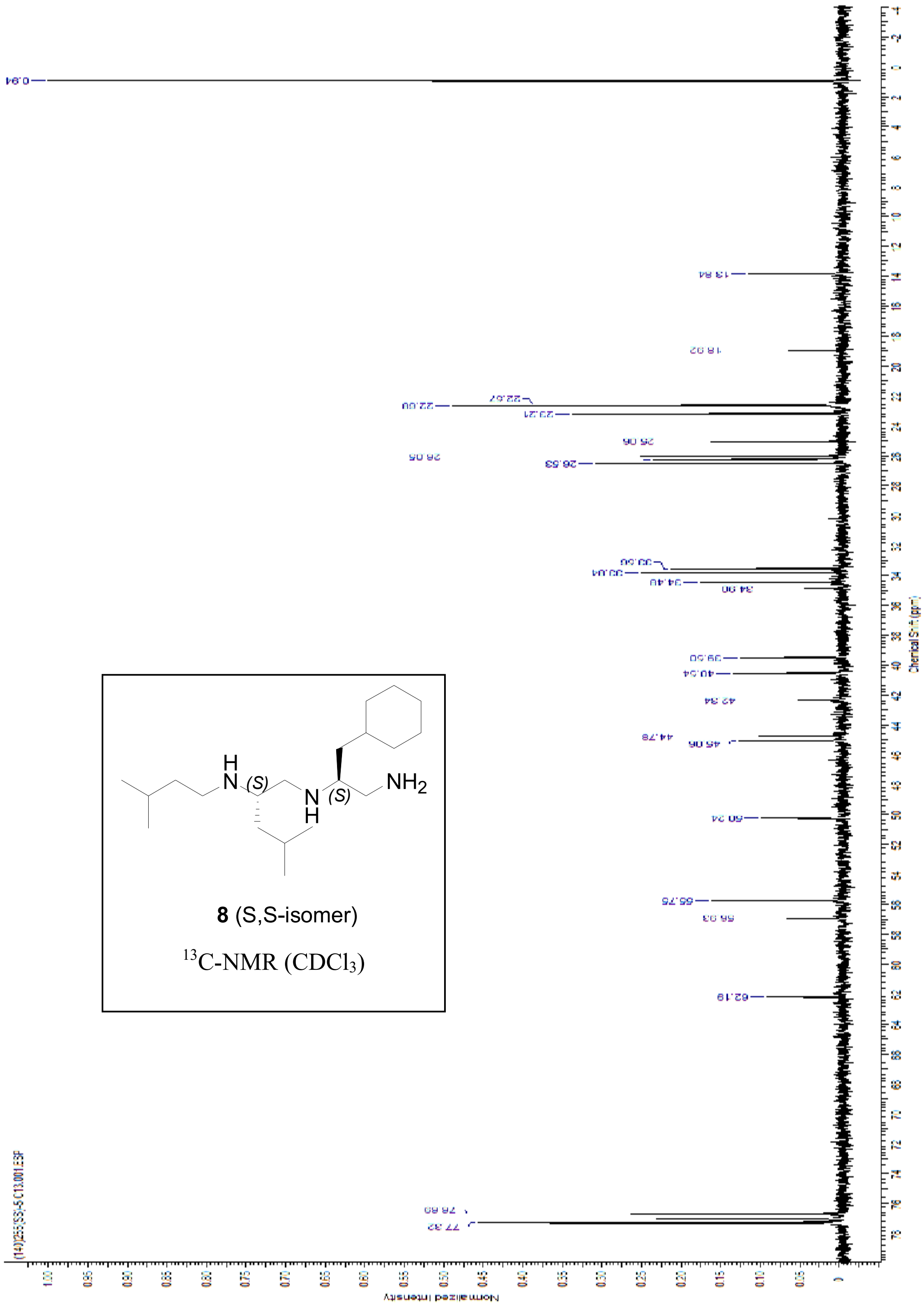

8 (S,S-isomer)

^{13}C-NMR ($CDCl_3$)

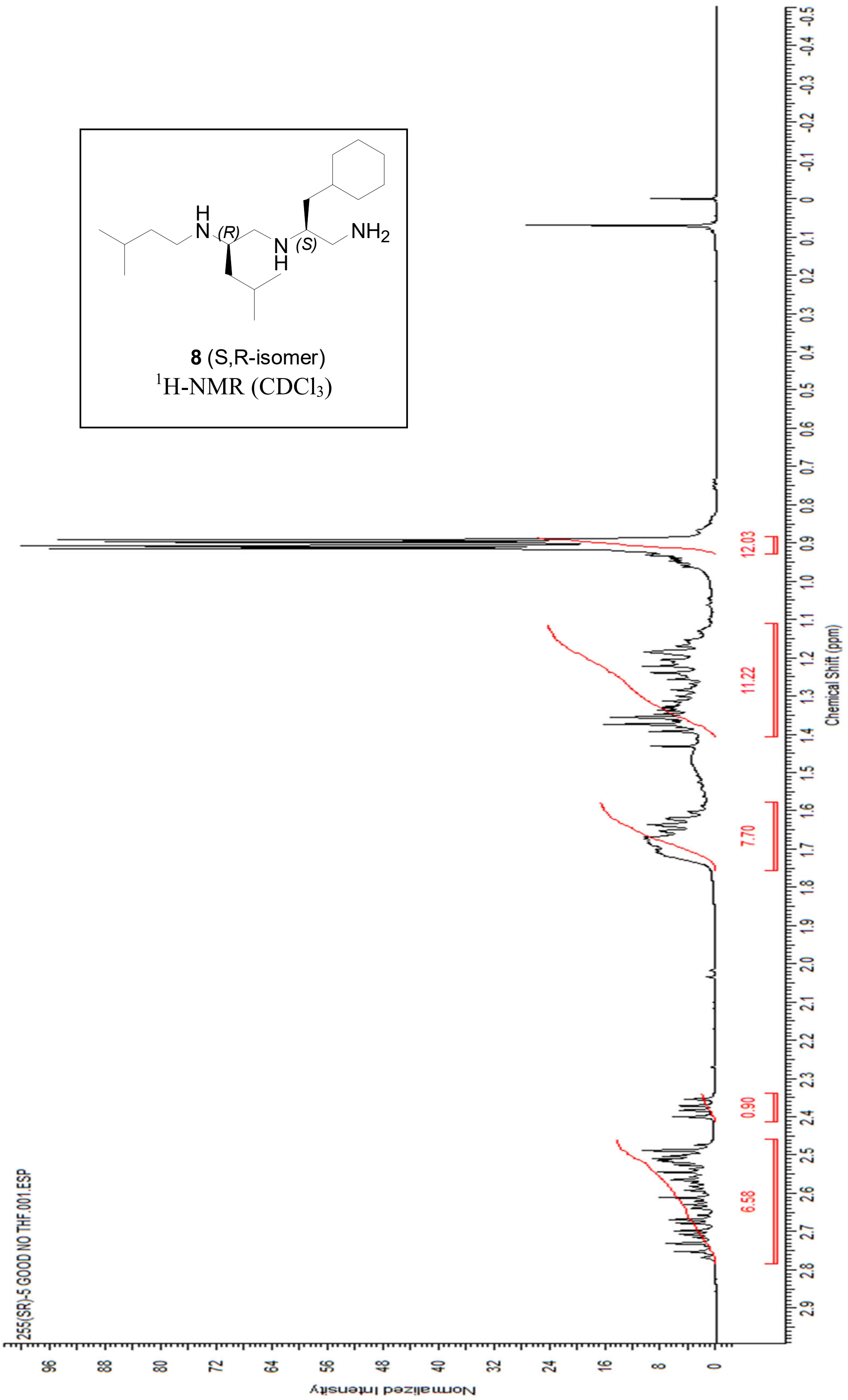

8 (S,R-isomer)
^{1}H-NMR ($CDCl_3$)

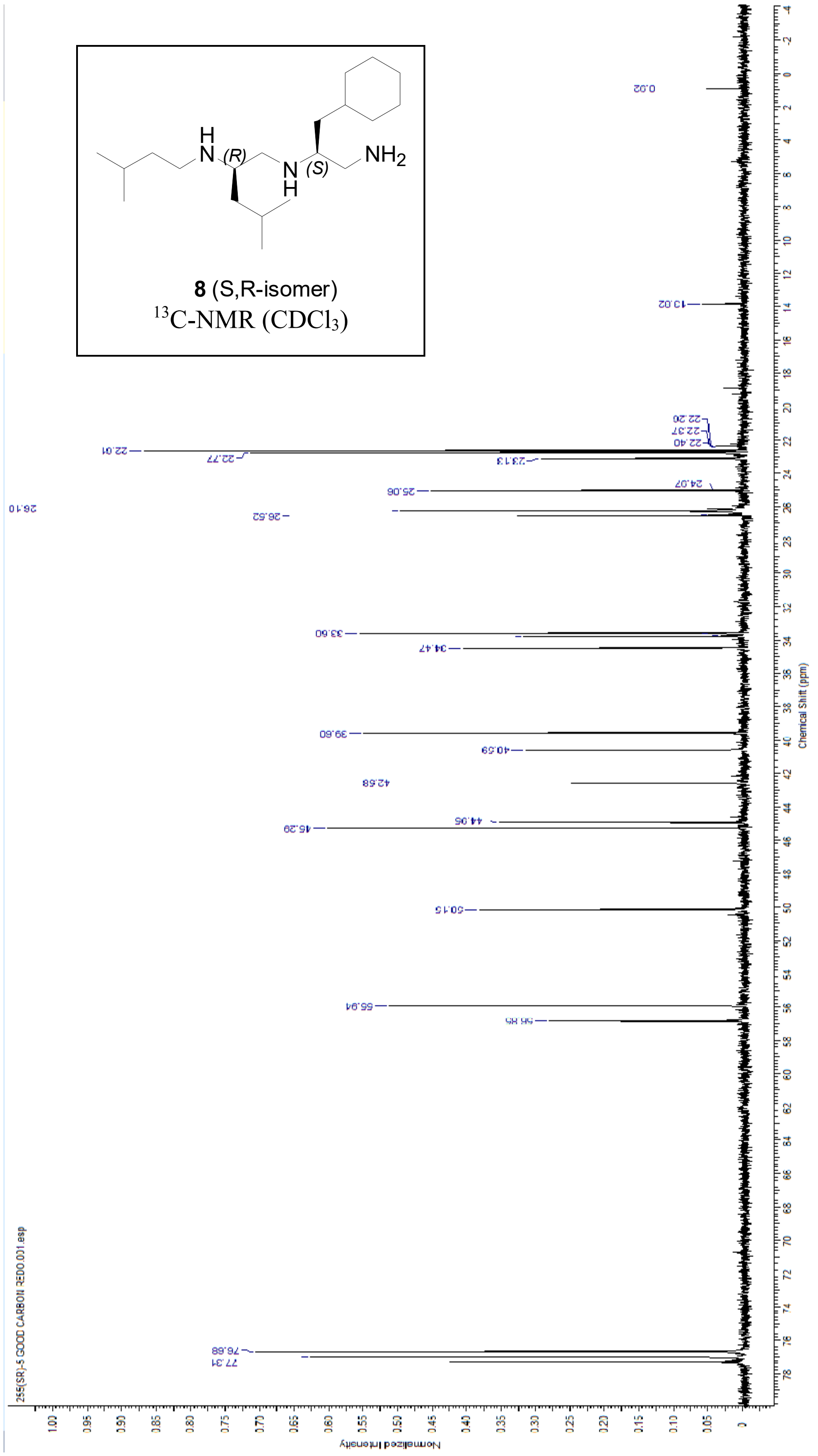

8 (S,R-isomer)
^{13}C-NMR ($CDCl_3$)

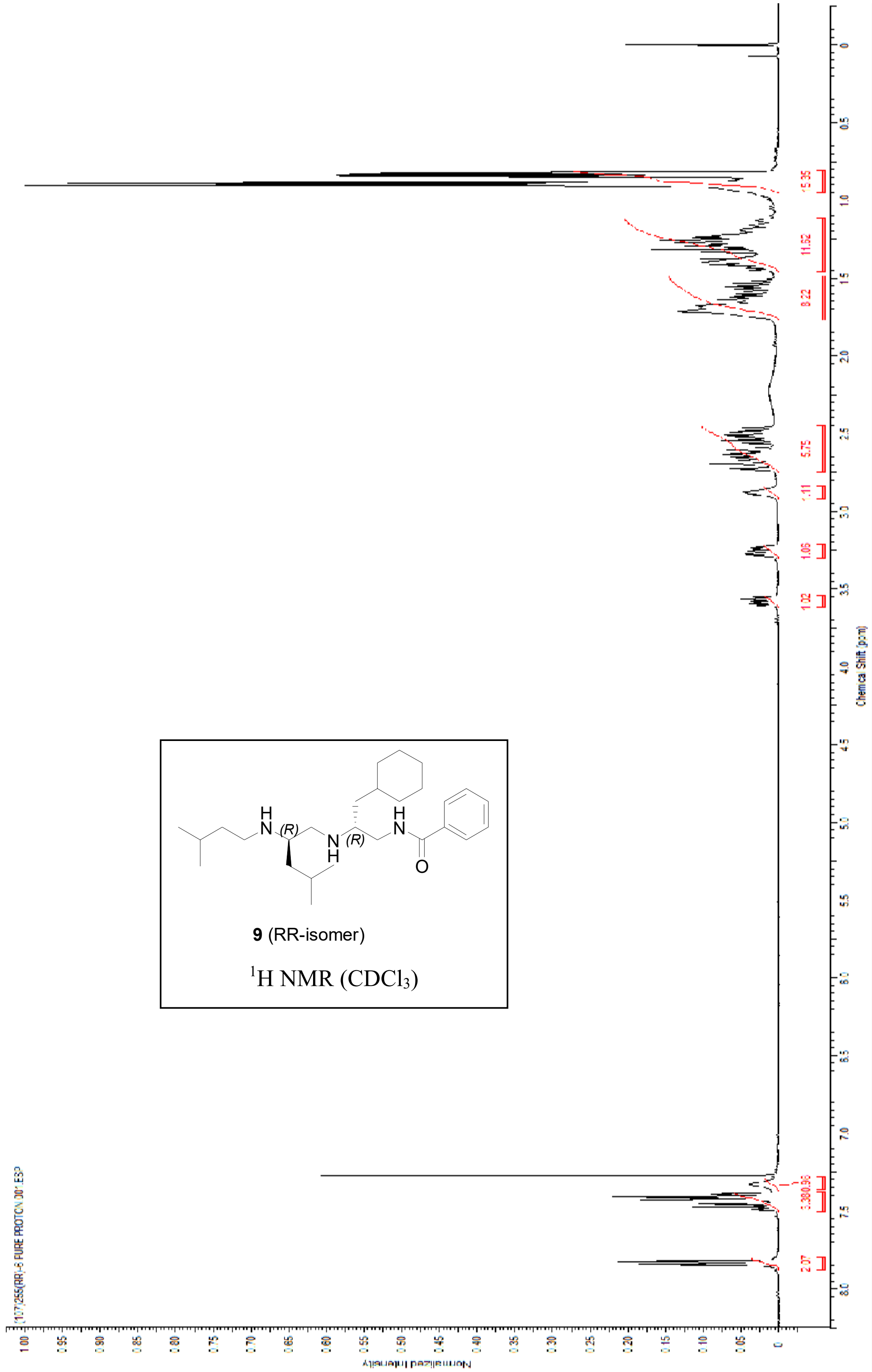

9 (RR-isomer)
^{1}H NMR ($CDCl_3$)
Chemical Shift (ppm)
Normalized Intensity

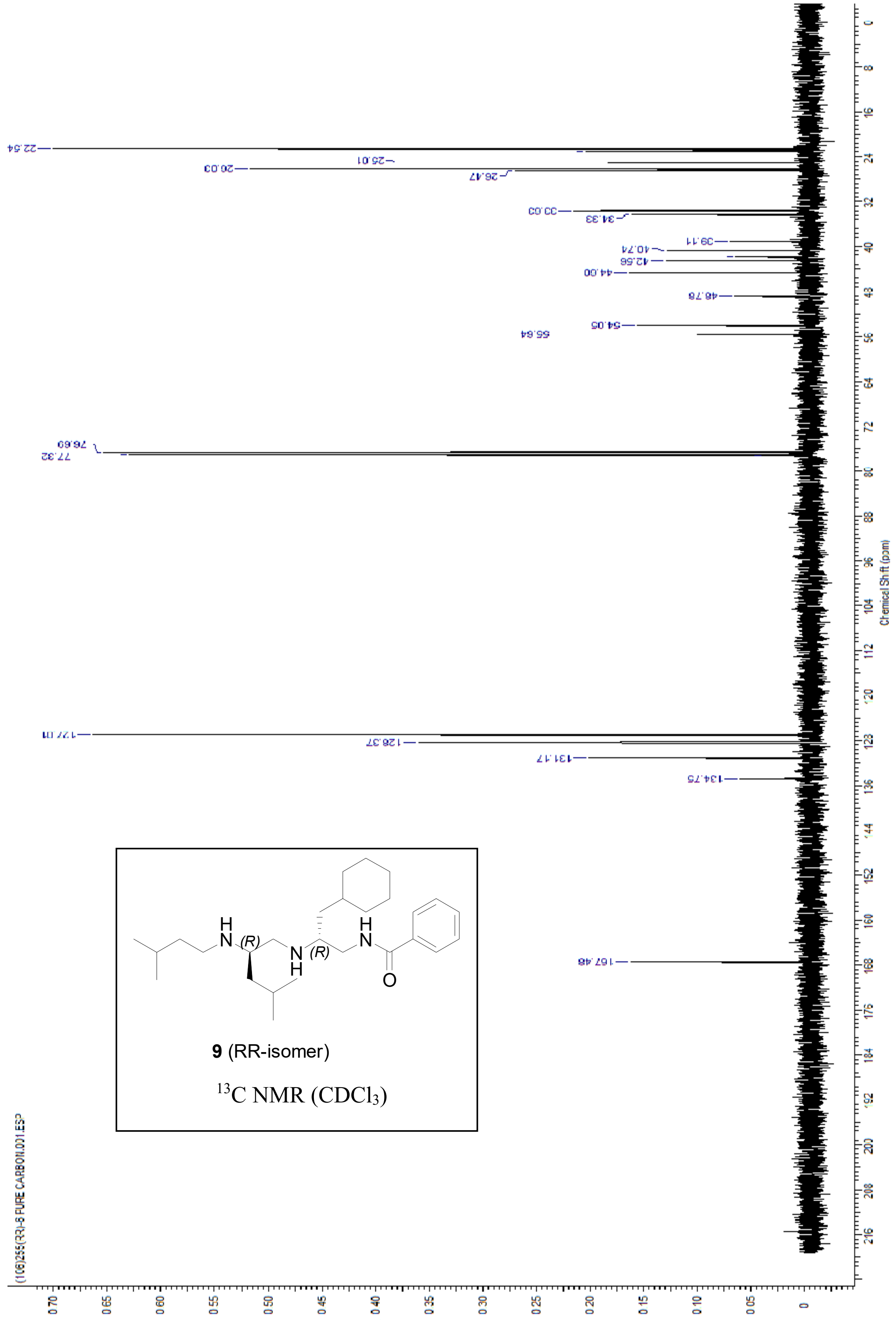

9 (RR-isomer)

^{13}C NMR ($CDCl_3$)

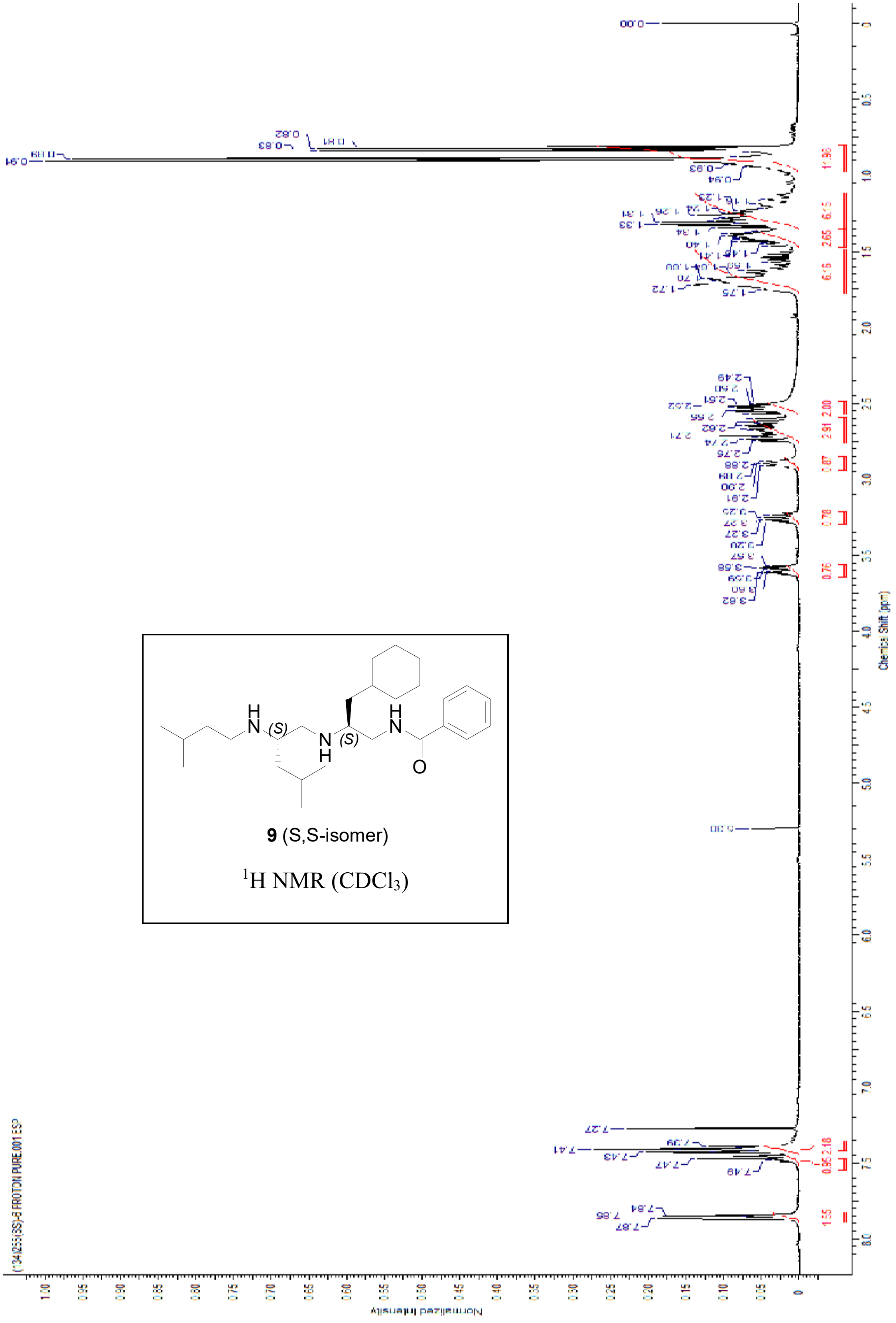

9 (S,S-isomer)

^{1}H NMR ($CDCl_3$)

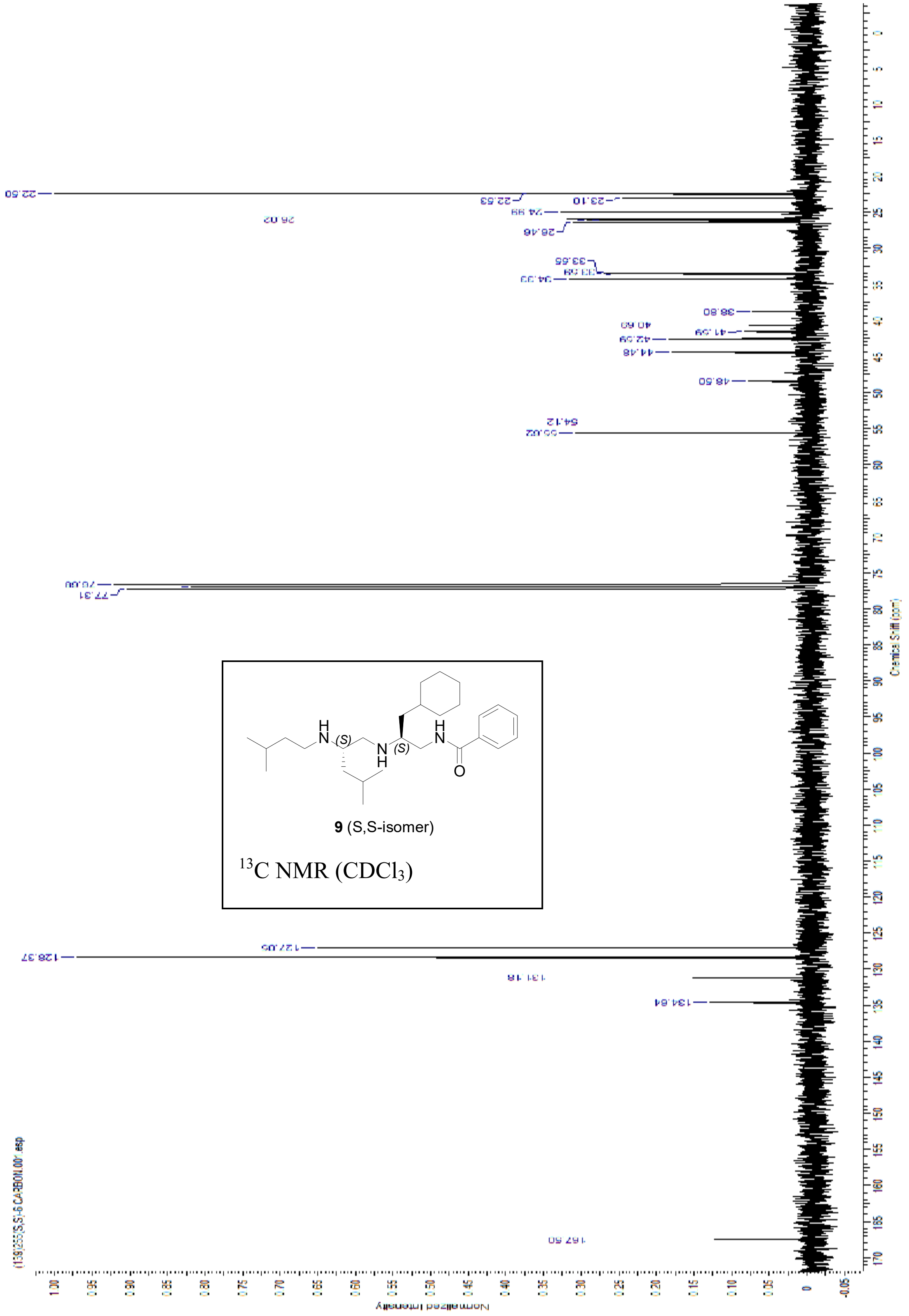

9 (S,S-isomer)
13C NMR (CDCl3)

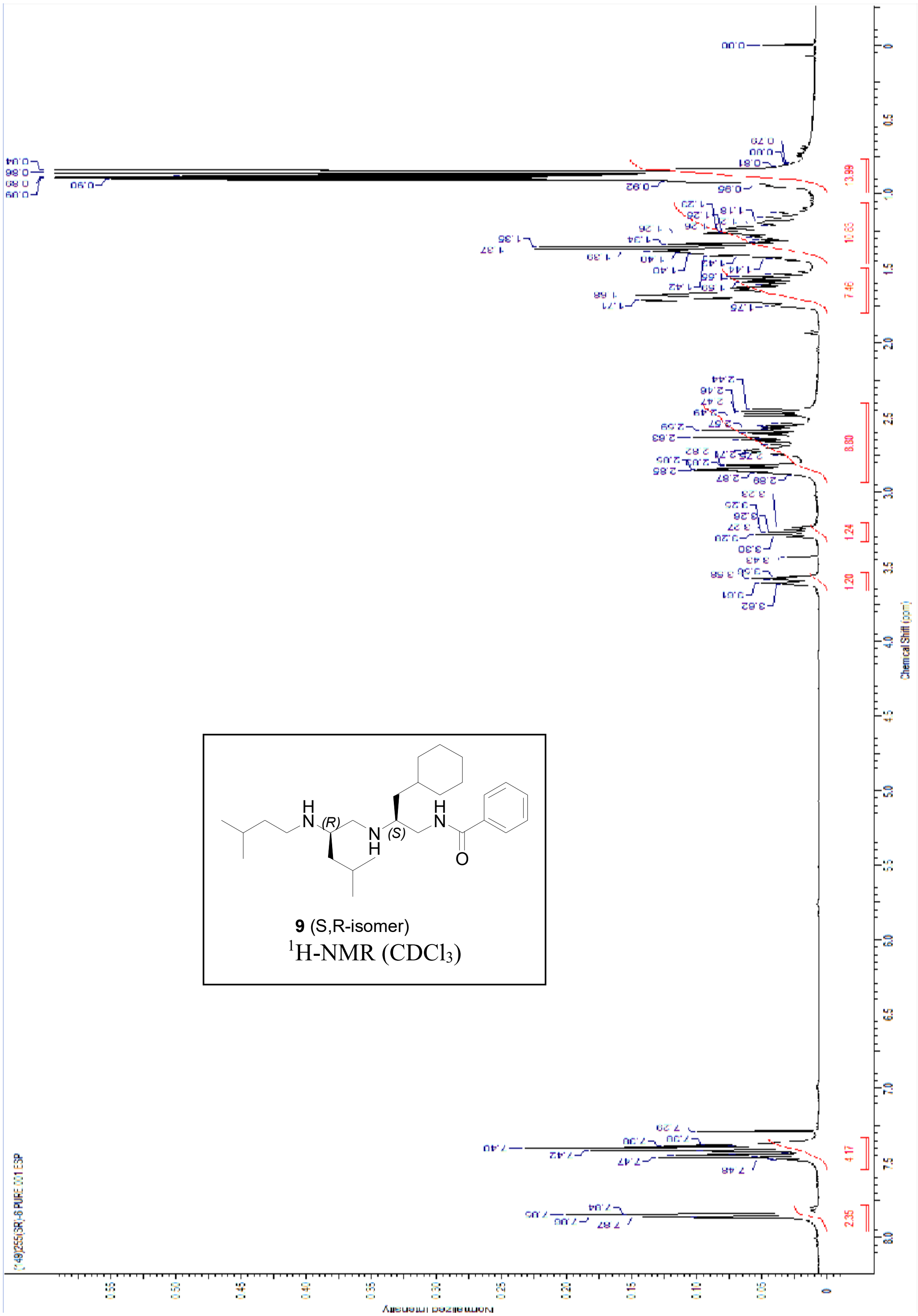

9 (S,R-isomer)

^{1}H-NMR ($CDCl_3$)

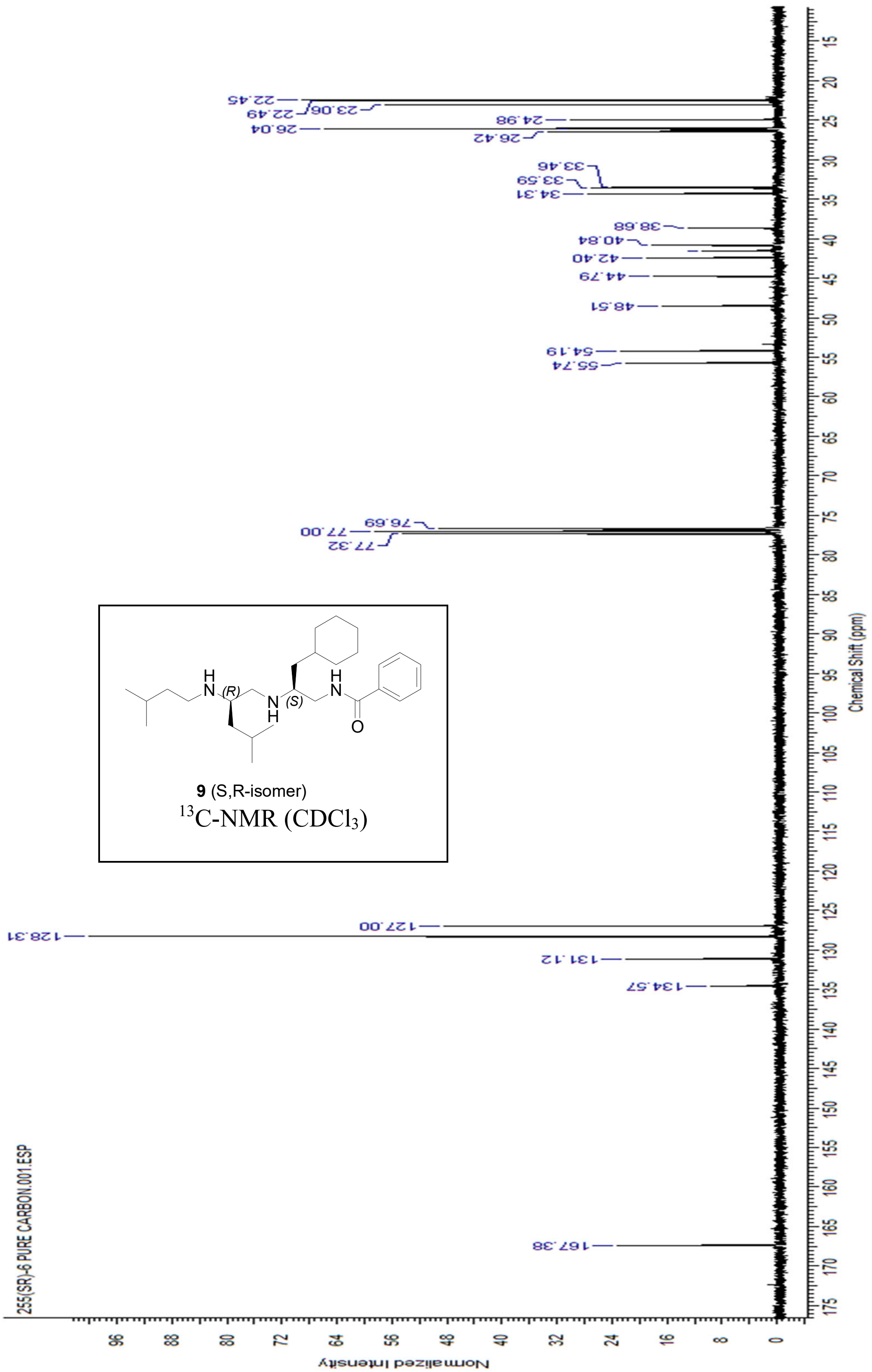
255(SR)-6 PURE CARBON.001.ESP
Chemical Shift (ppm)
Normalized Intensity
9 (S,R-isomer)
^{13}C-NMR ($CDCl_3$)

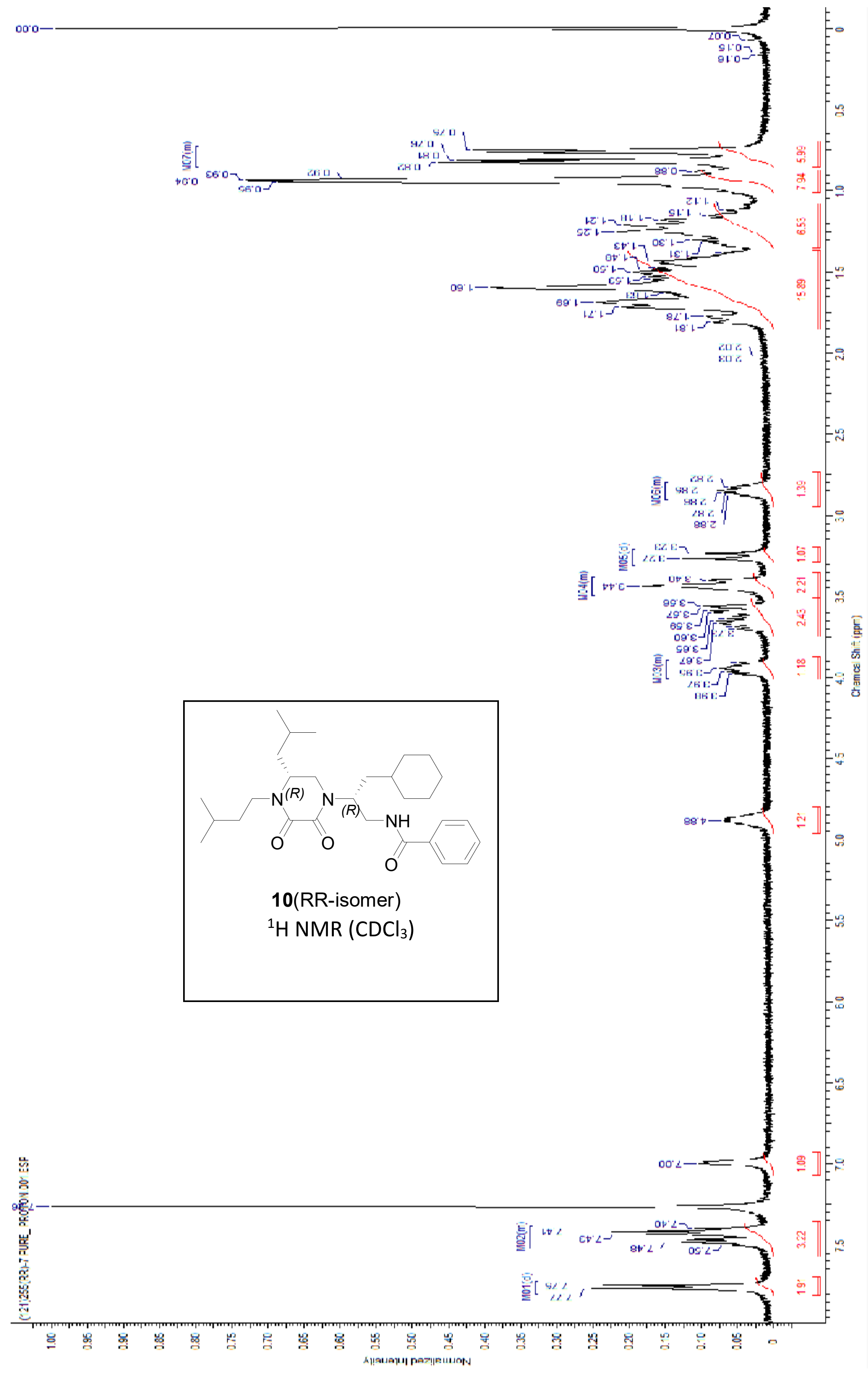

10(RR-isomer)
^{1}H NMR ($CDCl_3$)

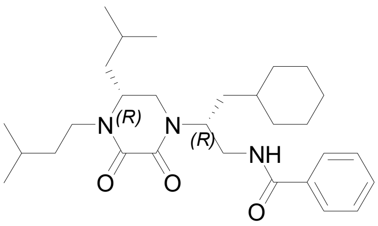

10(RR-isomer)

^{13}C NMR ($CDCl_3$)

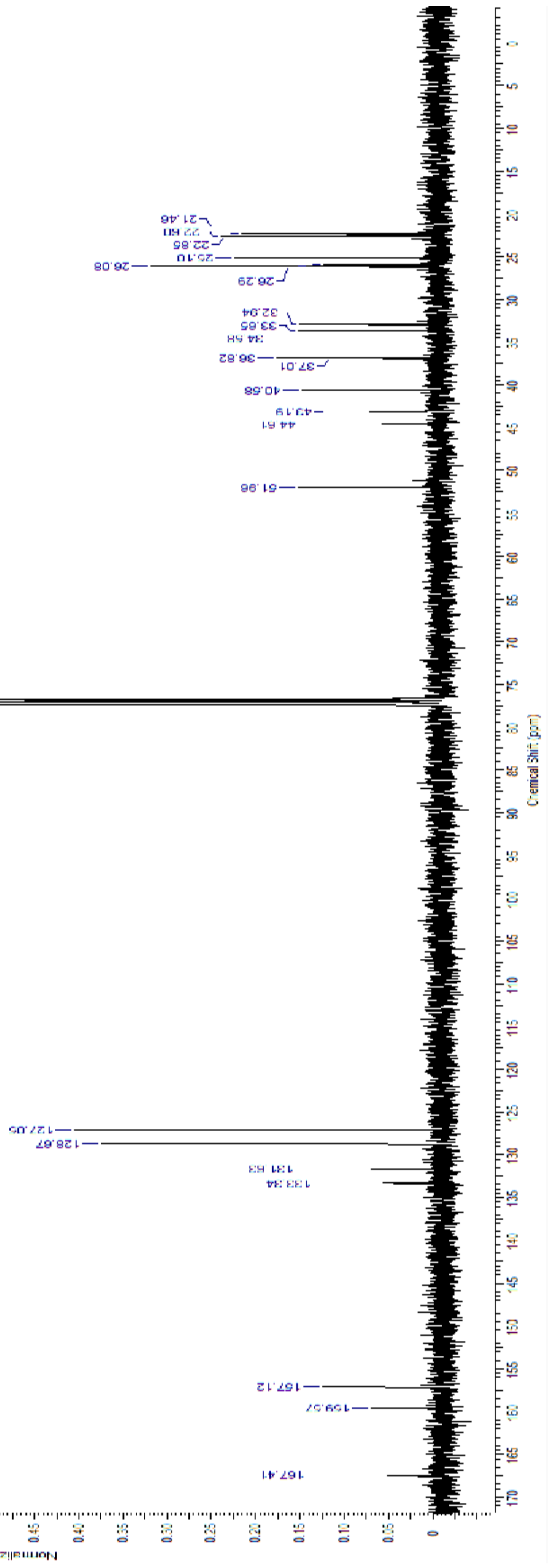

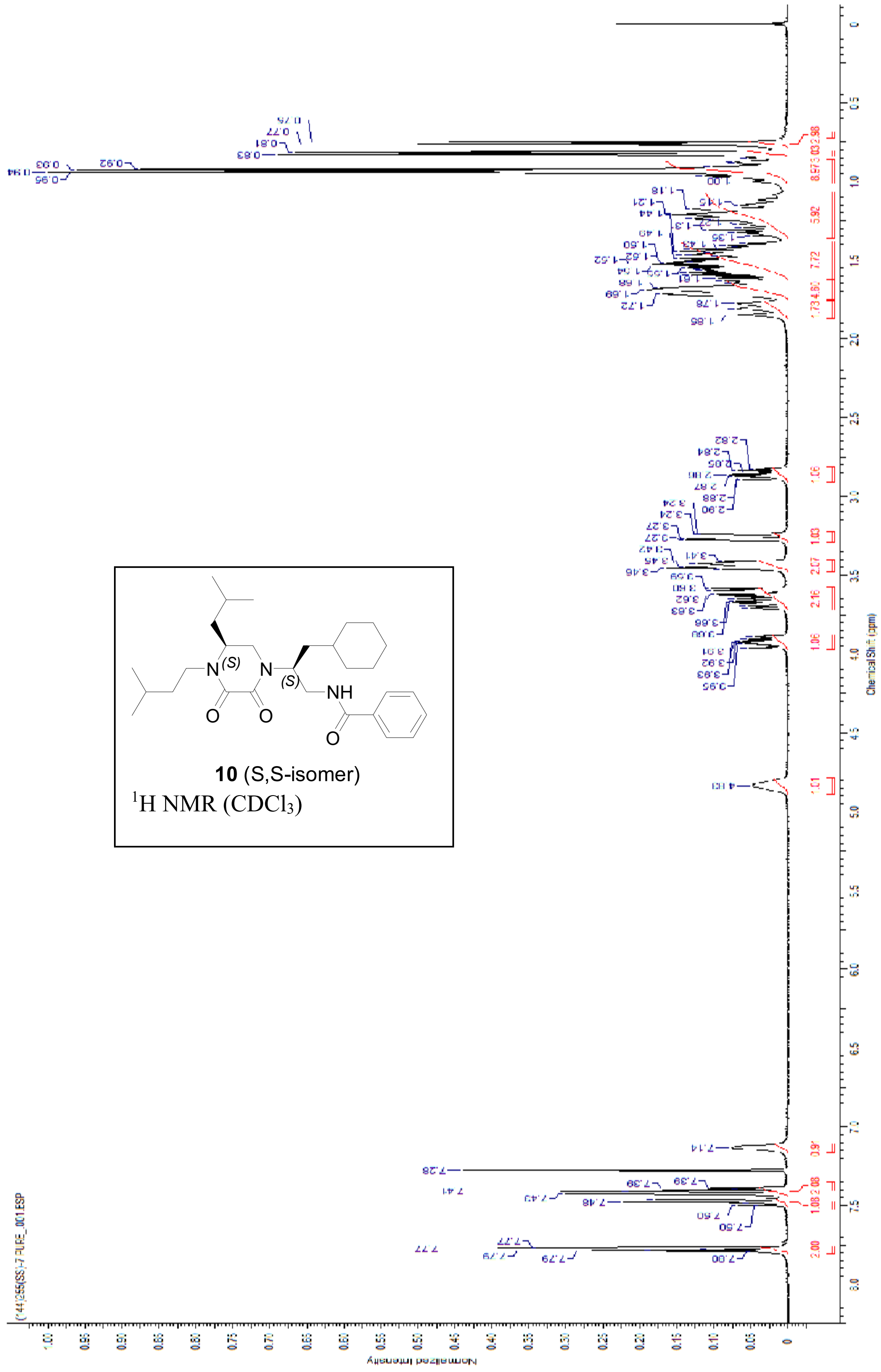

10 (S,S-isomer)
^{1}H NMR ($CDCl_3$)

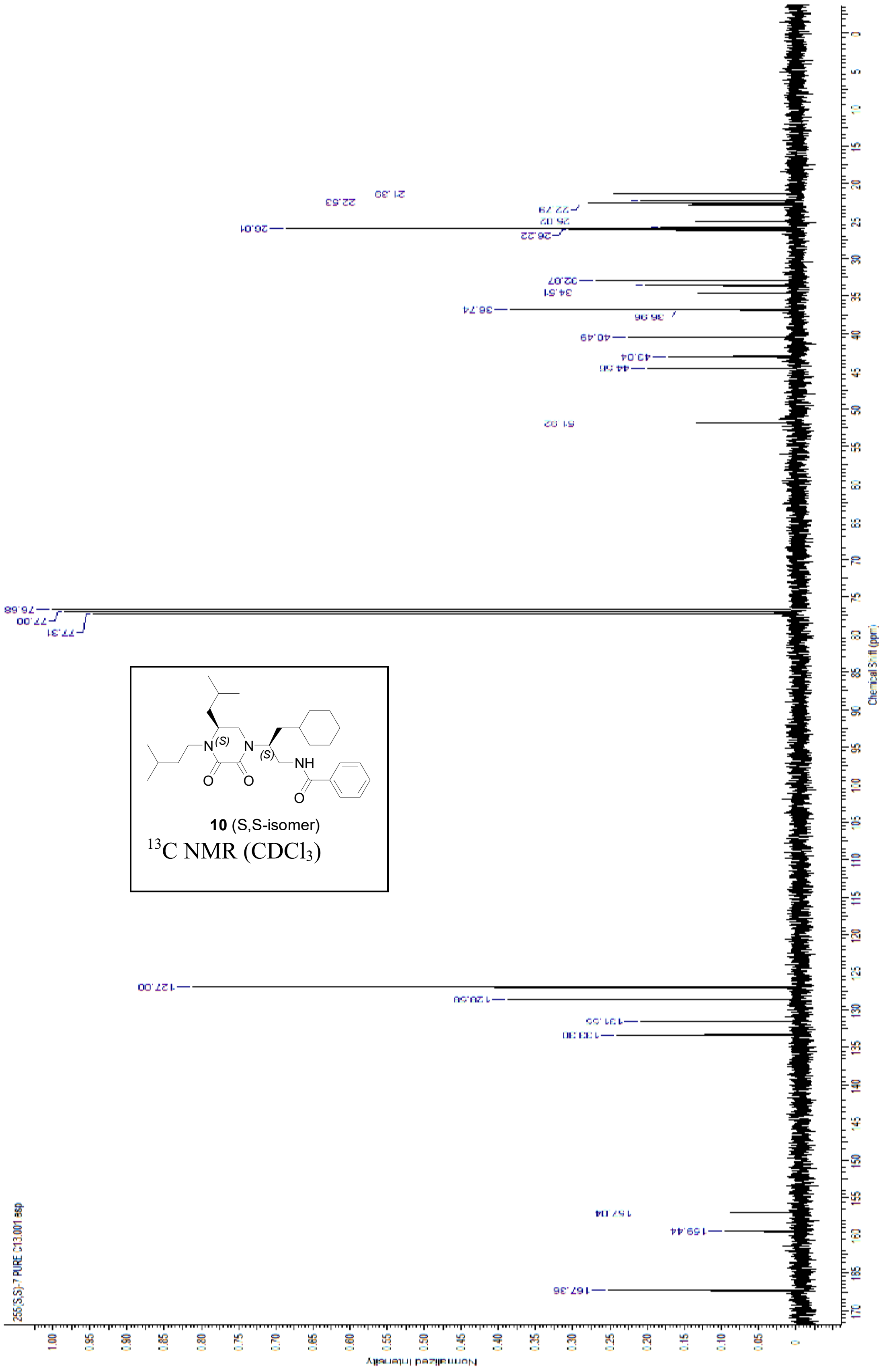
10 (S,S-isomer)
13C NMR (CDCl3)
Chemical Shift (ppm)
Normalized Intensity

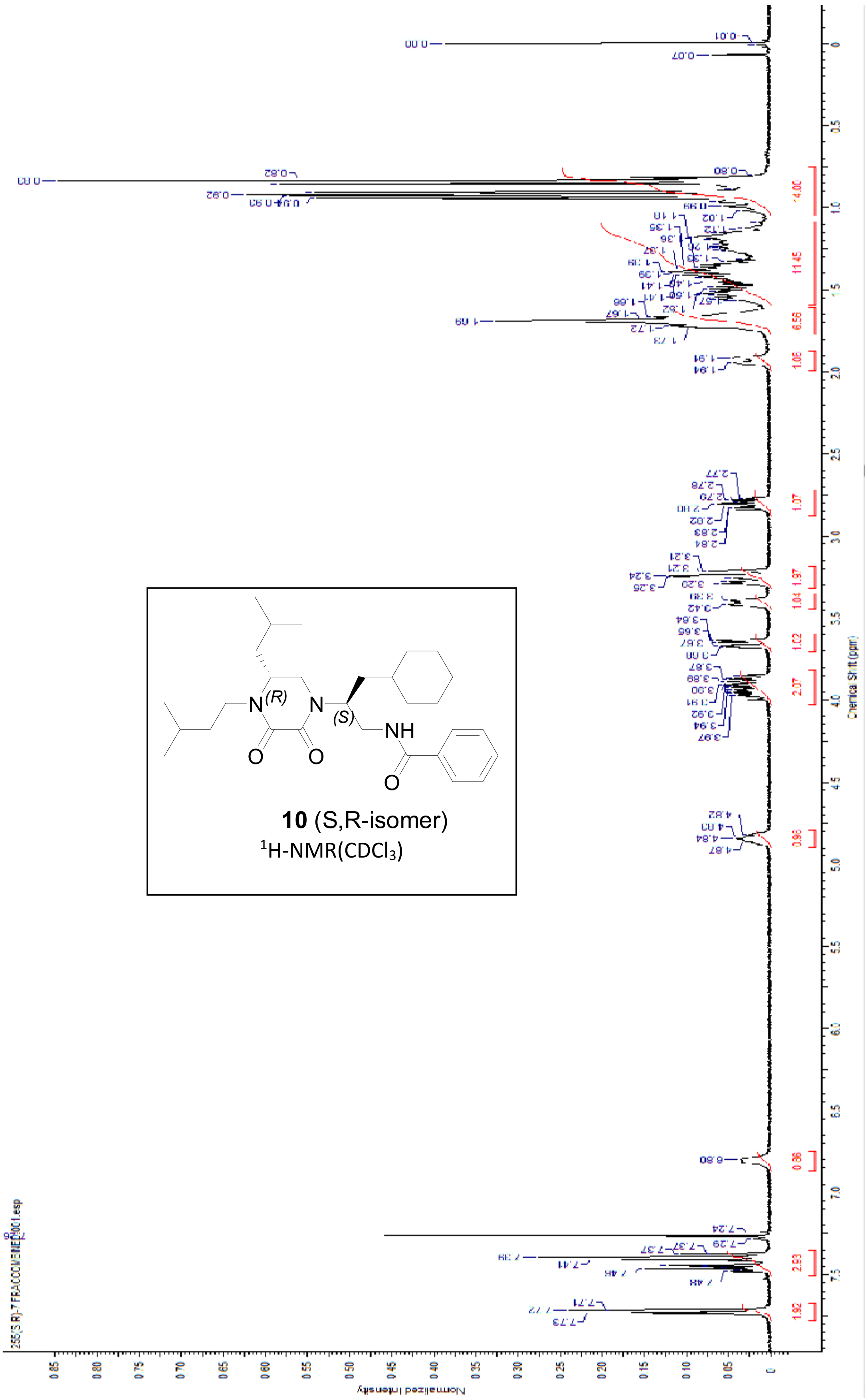
N(R)
N
(S)
NH
O
O
O
10 (S,R-isomer)
1H-NMR(CDCl3)

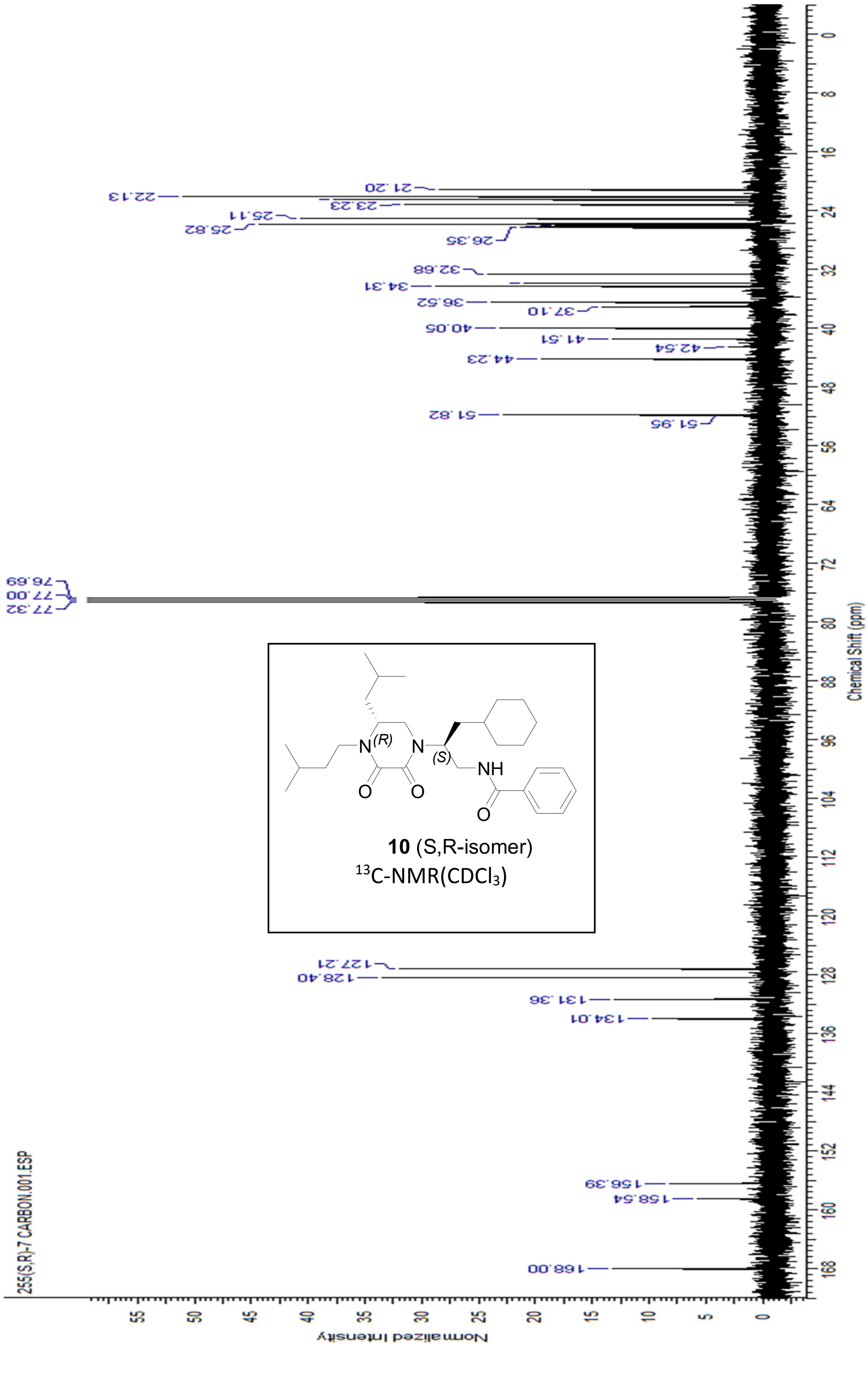

255(S,R)-7 CARBON.001.ESP
168.00
158.54
156.39
134.01
131.36
128.40
127.21
77.32
77.00
76.69
51.95
51.82
44.23
42.54
41.51
40.05
37.10
36.52
34.31
32.68
26.35
25.82
25.11
23.23
22.13
21.20
10 (S,R-isomer)
13C-NMR(CDCl3)
Chemical Shift (ppm)
Normalized Intensity

Compound	24h		48h		72h	
	MTC (µM)	IC50 (µM)	MTC (µM)	IC50 (µM)	MTC (µM)	IC50 (µM)
**1** (RS)	4.36 ± 0.34	6.54 ± 0.14	2.24 ± 0.11	4.28 ± 0.22	2.46 ± 0.28	3.50 ± 0.40
**1** (RR)	4.64 ± 0.47	6.56 ± 0.13	2.39 ± 0.14	4.07 ± 0.24	2.51 ± 0.18	3.30 ± 0.24

Table S2. Compound **1** (RS) and Compound **1** (RR) studies showing an increase in potency at longer incubation times, when plated at 2000 cells per well (24h expt), 1000 cells per well (48h expt) and 500 cells per well (72h expt). (B) Compound **1** (RS) and Compound **1** (RR) maximum tolerated concentration where growth remains ≥90% of the untreated control (MTC) values and IC_{50} values in L3.6pl cells over 24h, 48h, and 72h. At least 3 independent experiments were performed. Data are presented as mean ± SEM.

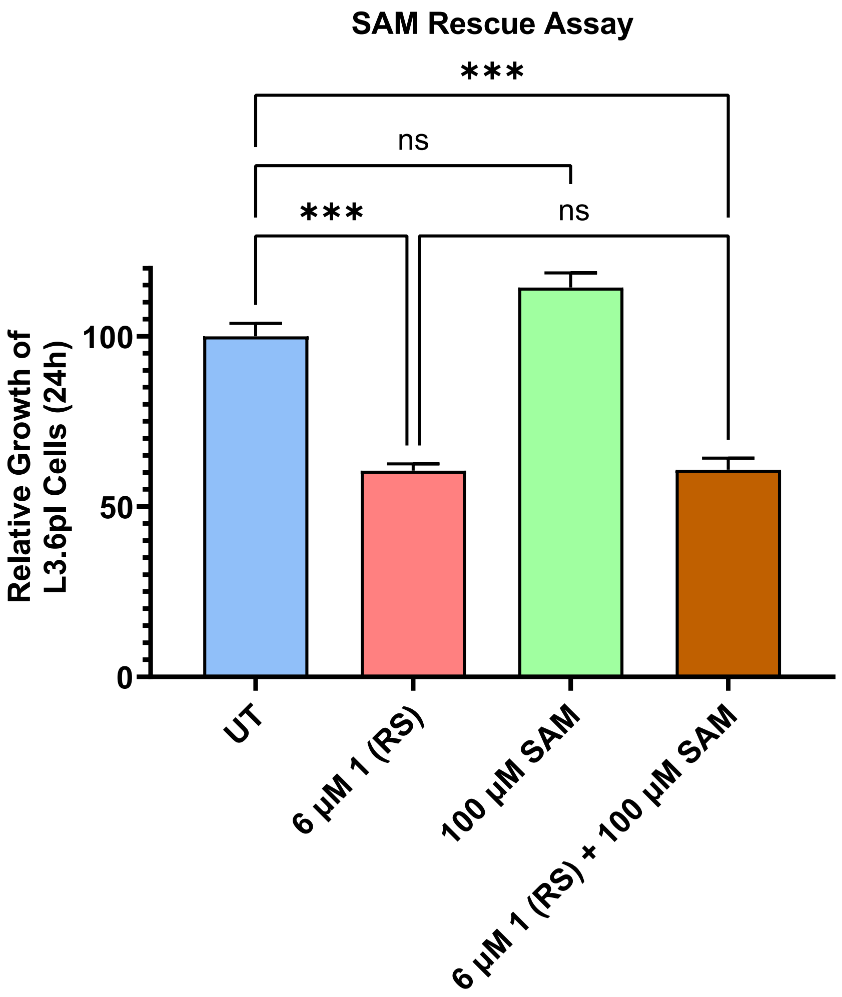

Figure S4. Investigation showing the inability of exogenous *S*-Adenosyl methionine (SAM) to rescue L3.6pl cells treated with Compound **1** (RS). (A) Relative growth of L3.6pl cells over 24h with the IC_{50} dose of Compound **1** (RS), with SAM, and in combination. At least 3 independent experiments were performed. Data are presented as mean ± SEM. ***p < 0.001.

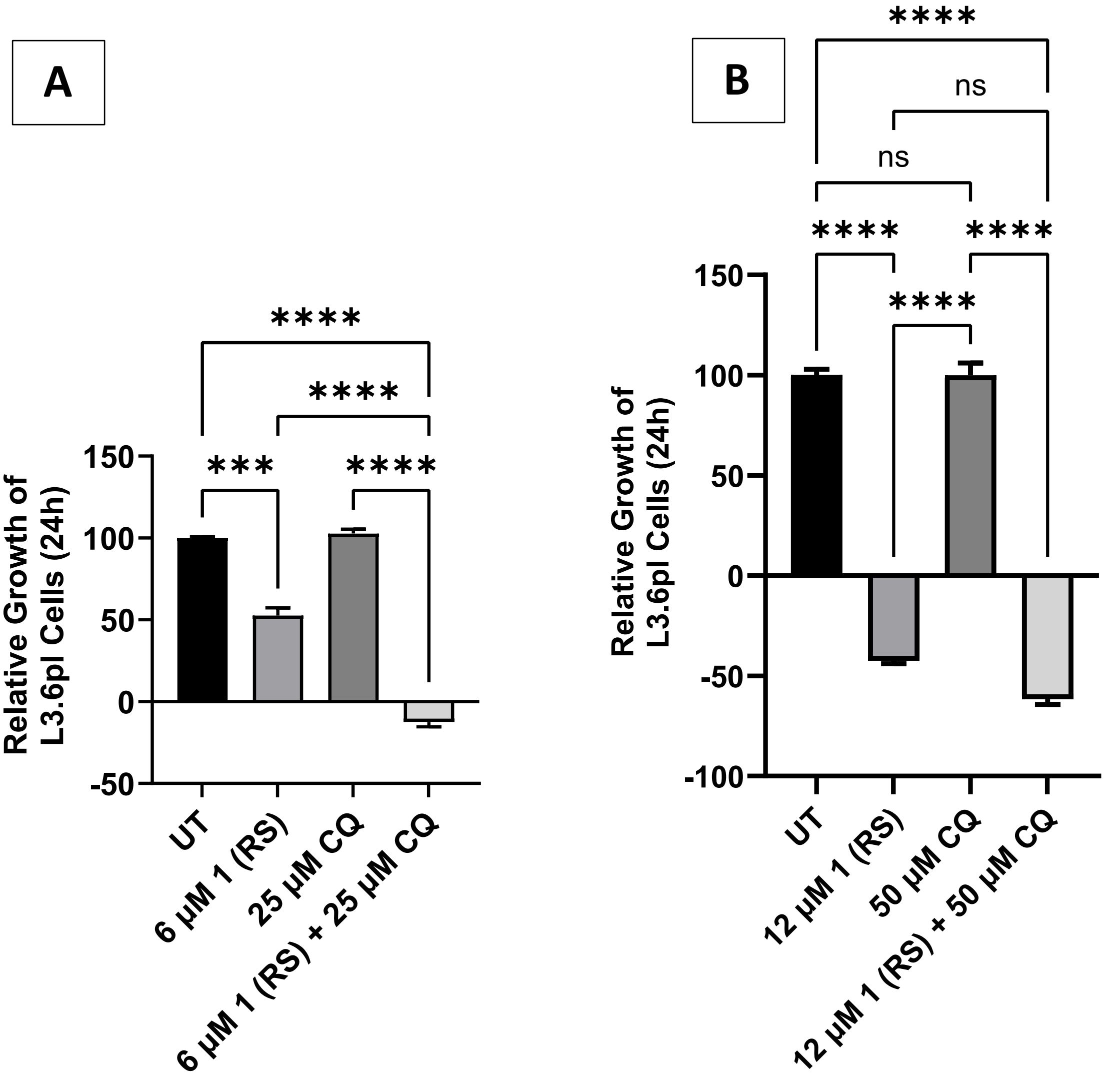

Figure S5. Investigation of Chloroquine (**CQ**) in combination with Compound **1** (RS) in L3.6pl pancreatic cancer cells. Note: L3.6pl cells plated at 2000 cells per well density. (**A**) Relative growth of L3.6pl cells over 24h in the presence of Compound **1** (RS; 6 µM), with CQ at 25 µM, and in combination. (**B**) Relative growth of L3.6pl cells over 24h in the presence of Compound **1** (RS; 12 µM), with CQ at 50 µM, and in combination. At least 3 independent experiments were performed. UT= untreated. Data are presented as mean ± SEM. ****p* < 0.001, *****p* < 0.0001.

Raw Western Blot Images. Note: 'RR', 'RS', 'SR', and 'SS' are the four isomers of compound 1.

The below two scans were used for **Figure 5**.

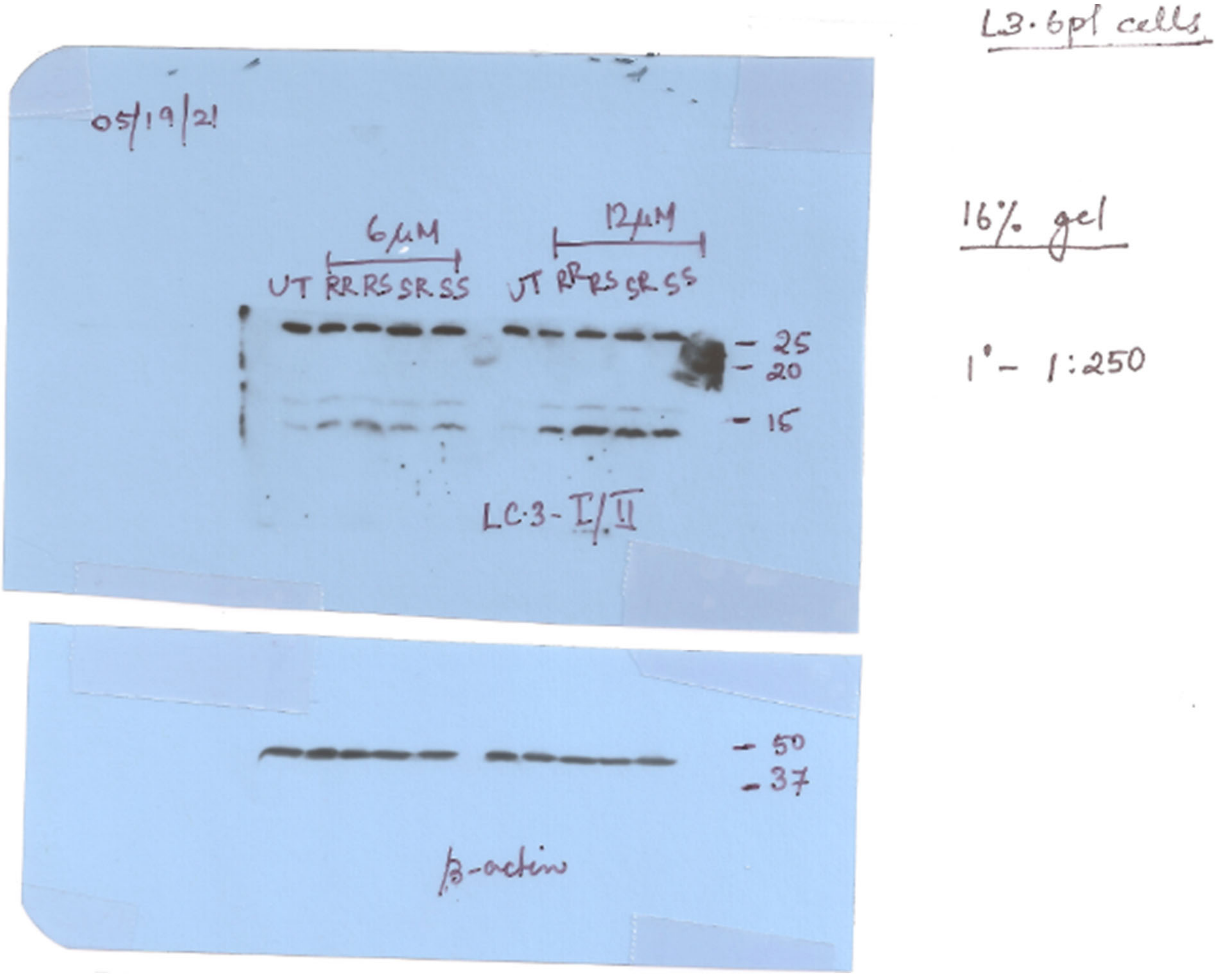

The below two scans were used for **Figure 6**.

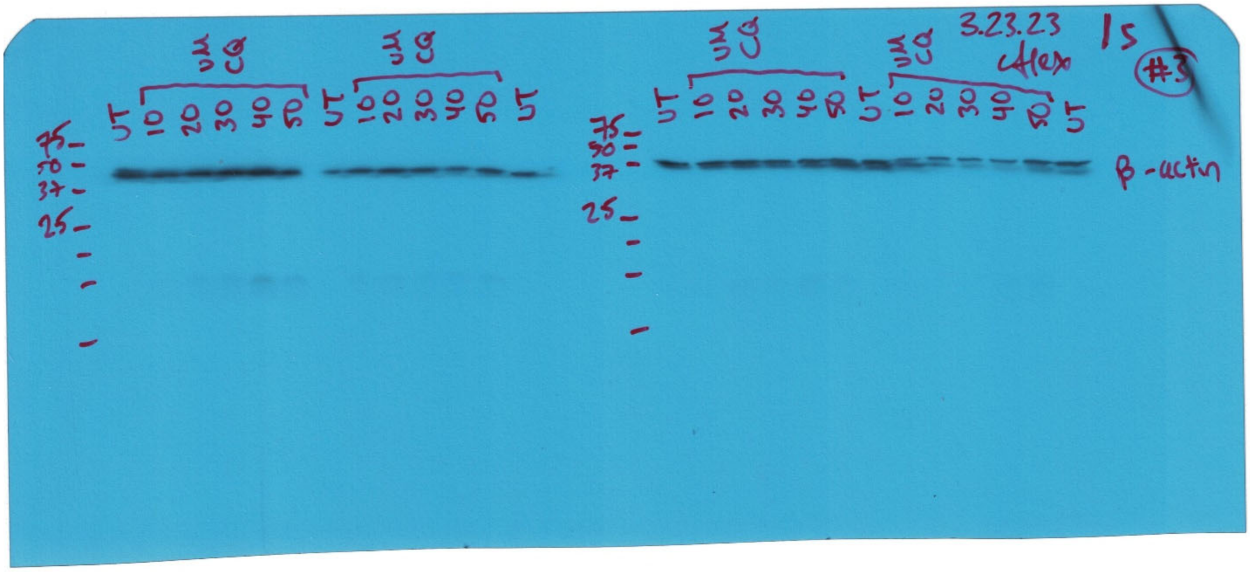

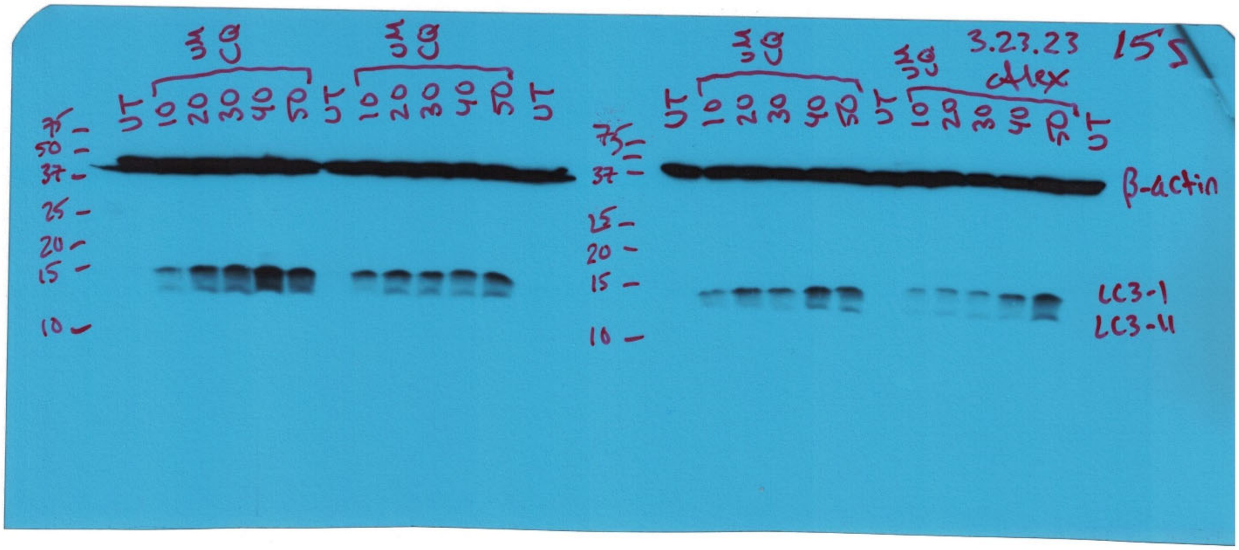

Raw Western Blot Images. Note: ‘255 RS’ is the RS isomer of compound 1 and CQ is chloroquine. Different exposure times (1 second vs 15 seconds) provided quantification of β-actin and the relevant LC3-II protein.

The below two scans were used for **Figure 7**.

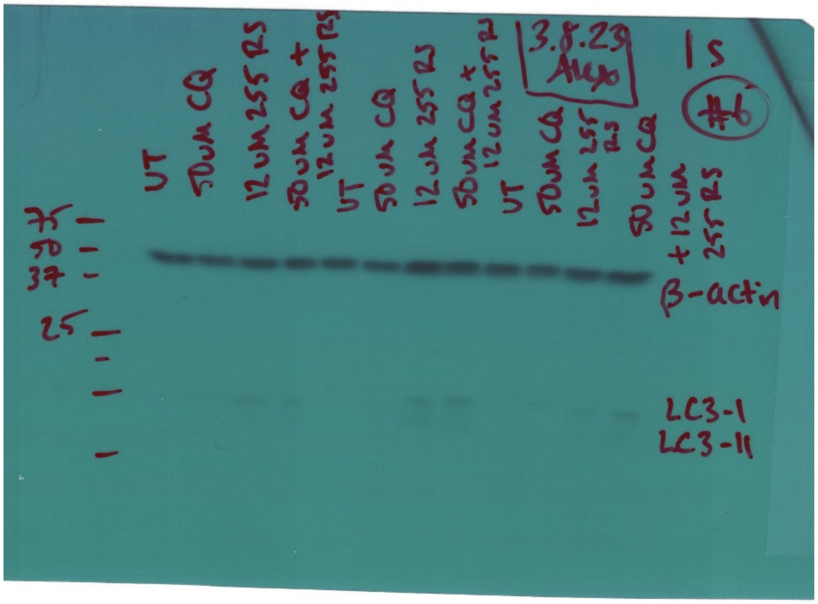

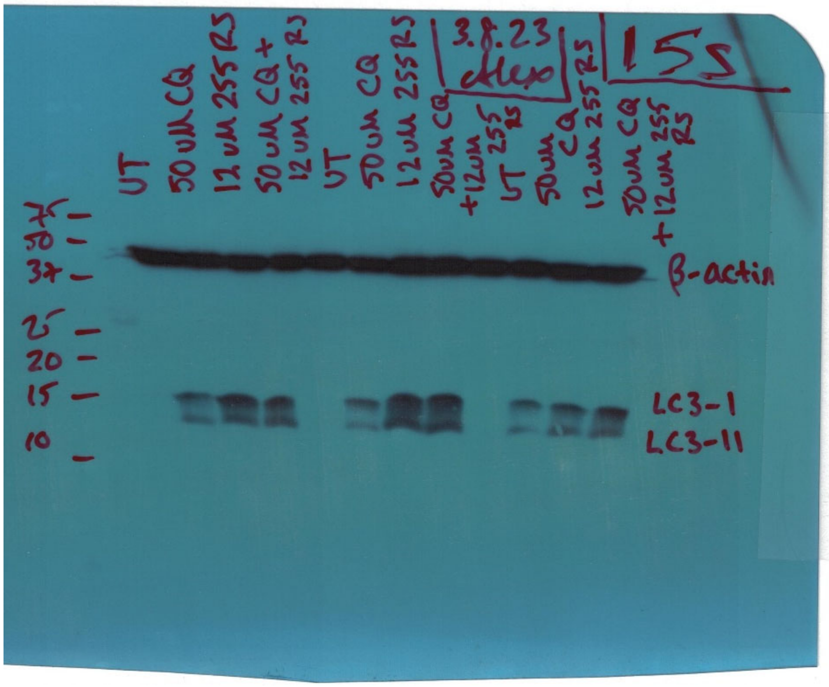

The below two scans were used for **Figure S2 (A)**.

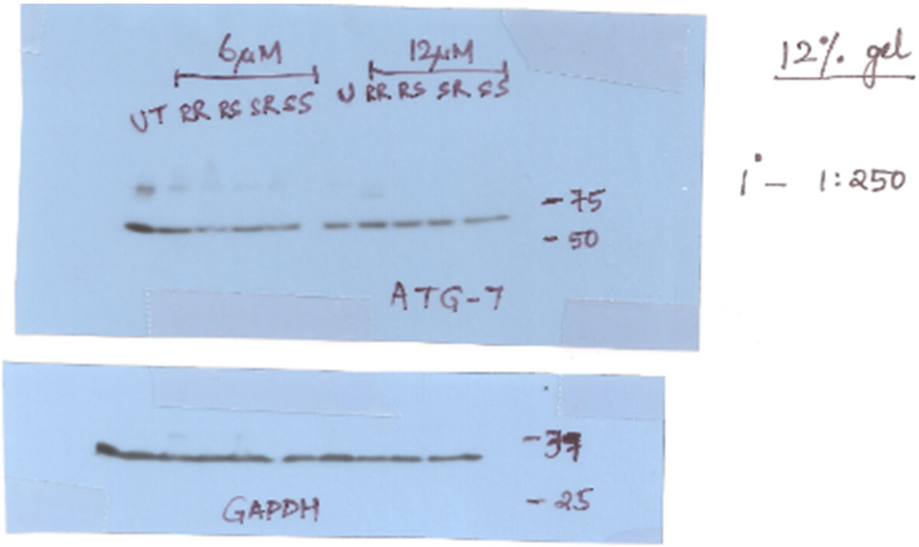

The below two scans were used for **Figure S2 (B)**.

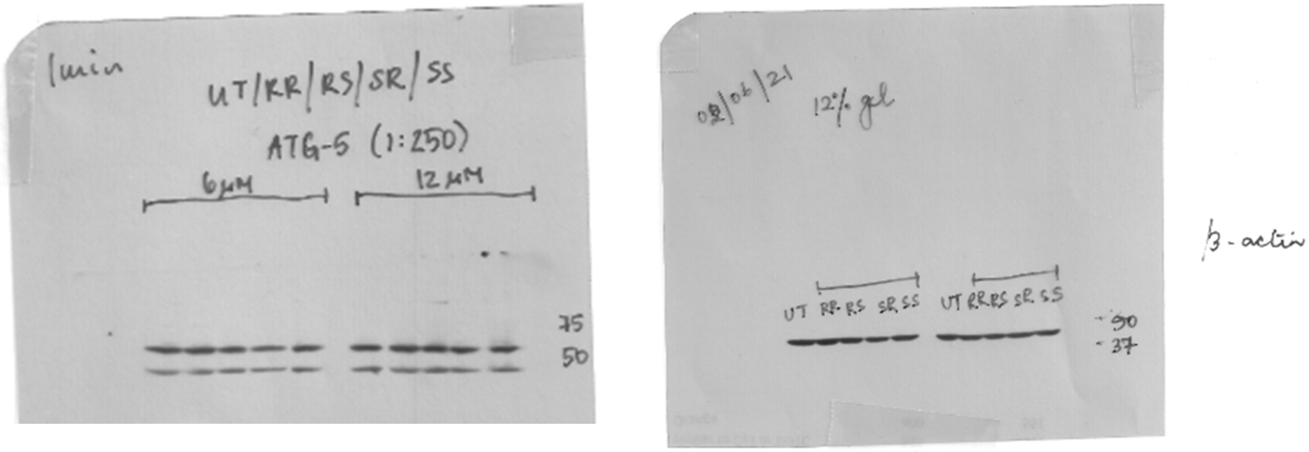

Supplement: Supplementary file 1 [file biomolecules-14-00326-s001.zip › biomolecules-2889789-supplementary.pdf]
